# Inhibition of α-Synuclein Fibrillization by Dopamine Is Mediated by Interactions with Five C-Terminal Residues and with E83 in the NAC Region

**DOI:** 10.1371/journal.pone.0003394

**Published:** 2008-10-14

**Authors:** Fernando E. Herrera, Alessandra Chesi, Katerina E. Paleologou, Adrian Schmid, Adriana Munoz, Michele Vendruscolo, Stefano Gustincich, Hilal A. Lashuel, Paolo Carloni

**Affiliations:** 1 International School for Advanced Studies (SISSA), INFM DEMOCRITOS, Trieste, Italy; 2 Italian Institute of Technology- SISSA Unit, Trieste, Italy; 3 Sector of Neurobiology, International School for Advanced Studies (SISSA), Basovizza, Trieste, Italy; 4 The Giovanni Armenise-Harvard Foundation Laboratory, Sector of Neurobiology, International School for Advanced Studies (SISSA), AREA Science Park, Trieste, Italy; 5 Laboratory of Molecular Neurobiology and Neuroproteomics, Brain Mind Institute, Ecole Polytechnique Federale de Lausanne (EPFL), Lausanne, Switzerland; 6 Department of Chemistry, University of Cambridge, Lensfield Road, Cambridge, United Kingdom; University of Cincinnati, United States of America

## Abstract

The interplay between dopamine and α-synuclein (AS) plays a central role in Parkinson's disease (PD). PD results primarily from a severe and selective devastation of dopaminergic neurons in substantia nigra pars compacta. The neuropathological hallmark of the disease is the presence of intraneuronal proteinaceous inclusions known as Lewy bodies within the surviving neurons, enriched in filamentous AS. *In vitro*, dopamine inhibits AS fibril formation, but the molecular determinants of this inhibition remain obscure. Here we use molecular dynamic (MD) simulations to investigate the binding of dopamine and several of its derivatives onto conformers representative of an NMR ensemble of AS structures in aqueous solution. Within the limitations inherent to MD simulations of unstructured proteins, our calculations suggest that the ligands bind to the ^125^YEMPS^129^ region, consistent with experimental findings. The ligands are further stabilized by long-range electrostatic interactions with glutamate 83 (E83) in the NAC region. These results suggest that by forming these interactions with AS, dopamine may affect AS aggregation and fibrillization properties. To test this hypothesis, we investigated *in vitro* the effects of dopamine on the aggregation of mutants designed to alter or abolish these interactions. We found that point mutations in the ^125^YEMPS^129^ region do not affect AS aggregation, which is consistent with the fact that dopamine interacts non-specifically with this region. In contrast, and consistent with our modeling studies, the replacement of glutamate by alanine at position 83 (E83A) abolishes the ability of dopamine to inhibit AS fibrillization.

## Introduction

Parkinson's disease (PD) is the second most common progressive neurodegenerative disorder, affecting 1–2% of the population over 65 [1], [2]. The clinical symptoms of PD include muscle rigidity, resting tremor, bradykinesia and gait disturbance with disequilibrium. Neuropathologically, PD is characterized by a selective degeneration of specific subsets of mesencephalic dopaminergic cells in the brain and the formation of cytoplasmic aggregates called Lewy bodies (LBs). The major proteinaceous building block of LBs are insoluble fibrils made up of the α-synuclein (AS) protein [3], suggesting that the aggregation of this protein may play a central role in the development and/or progression of the disease. This idea is supported by evidence from genetics, animal modeling, cell culture and biophysical studies: **1)** increased production (gene duplication and triplication [4], [5]) and/or missense mutations (A53T A30P, and E46K) [6]–[8]) in the gene encoding for AS are linked to autosomal dominant inherited forms of familial PD; **2)** several lines of transgenic mice and flies that overexpress wild-type and disease-associated variants of AS show age-dependent formation of AS-containing inclusions, loss of dopaminergic cells and motor abnormalities [9], [10]; **3)** overexpression of AS causes cell death in cultured dopaminergic neurons and in differentiated neuroblastoma cells [11]; **4)** all PD associated mutations have been shown to accelerate and enhance the oligomerization and fibrillogenesis of AS *in vitro* [12], [13]; **5)** AS toxicity and fibrillization is influenced by factors that may be relevant to PD, including post-translational modifications, oxidative stress and interaction with toxins and metals [14]–[18]. Other neurodegenerative diseases are characterized by the accumulation of fibrillar AS, including a LB variant of Alzheimer's disease, dementia with LB, multiple system atrophy and related diseases, which collectively are referred as α-synucleinopathies [19].

The AS sequence (140 amino acids) can be divided into three different regions: (i) the positively charged N-terminal region (amino acids 1–60) comprising the seven imperfect 11 amino acids repeats containing the consensus sequence KTKEGV; (ii) the non-β-amyloid component (NAC) (amino acids 61–95); (iii) the negatively charged C-terminal region (amino acids 96–140), which contains several sites of post-translational modifications and metal binding.

Structural information derived from NMR studies for the monomeric structure of AS is increasingly used to study and model AS aggregation and its interaction with other proteins [20]–[22]. In aqueous solution, AS exists as a highly heterogeneous ensemble of conformations. NMR studies based on paramagnetic relaxation enhancement (PRE) effects generated an ensemble of about 4,000 structures characterized by transient long-range interactions [22]. Upon binding to SDS micelles and negatively charged synthetic vesicles [23], AS adopts a partially α-helical conformation: two α-helices (amino acids 1–38 and 44–94) are formed in a non-polar environment, whilst the remainder of the protein is disordered, as shown by NMR spectroscopy [23]. The monomer is prone to aggregation into amyloid-like structures, particularly at high concentrations or upon exposure to various chemical and physical factors (e.g. shaking). The AS fibrillization proceeds through a series of β-sheet-rich aggregation intermediates, including early spherical protofibrils, pore-like and chain-like aggregates, which disappear once amyloid fibrils are formed [12], [24]. Although mounting evidence points towards a prefibrillar species as the toxic entity, the identity of the exact toxic species and its mode of action remain unknown and are the subjects of intense study and debate [24]–[26].

The proposed biological functions for AS include the regulation of lipid metabolism [27]–[29], vesicle-mediated transport [28], trafficking within the endoplasmic reticulum/Golgi network [30], [31] and chaperone activity [32], [33]. In addition, AS has also been shown to regulate dopamine metabolism at multiple levels including its synthesis, uptake and release [29], [34]–[36]. Several *in vitro* and cell culture studies suggest that direct interactions between dopamine and AS play a central role in the pathogenesis of PD. Chatechol derivatives including dopamine have been shown to inhibit AS fibrillogenesis causing accumulation of oligomeric species *in vitro* [37], [38] and *in vivo* [39], [40]. These studies suggest that the oxidation of dopamine may play a key role in modulating AS aggregation and toxicity and may be linked to the selective vulnerability of dopaminergic neurons in PD [41]. Therefore, investigating the structural determinants of dopamine binding may shed light on the mechanisms by which this molecule modulates AS fibrillization and toxicity, thus providing new clues for therapeutics intervention in PD and related diseases.

**Figure 1:**
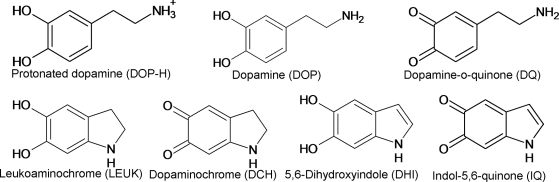
Dopamine docked onto AS: chemical formulas of the proposed dopamine forms binding to AS [42].

Here, we perform a series of molecular dynamic (MD) simulations and biophysical studies in order to identify both the residues and the nature of the interactions that mediate the binding of dopamine to structures of AS. Our modeling studies are based on structures of AS obtained by NMR spectroscopy [22] and MD simulations. Our calculations suggest that dopamine (DOP) as well as several products derived from its oxidation (Figure 1) [42], [43], bind to the C-terminal region comprising the residues ^125^YEMPS^129^, which is consistent with previous experimental findings [38], [40]. Such interactions are mostly hydrophobic in nature. In addition, our calculations indicate that AS-DOP interactions are further stabilized by long-range electrostatic interactions with glutamate 83 (E83) in the NAC region. We confirm these findings by *in vitro* fibrillization studies on AS mutants designed to either alter and/or abolish the specific interactions identified by our modeling studies.

## Materials and Methods

### Computational Chemistry

The initial models of AS•dopamine adducts are based on selected AS conformations from NMR [22] and MD calculations, performed by us here.

#### Adducts based on NMR

We considered the 3,062 conformers of ref. [22] that exhibit five or less amino acids (not Gly) in the forbidden regions of the Ramachandran plot, as defined by the “what_check” algorithm [44]. We used a method that clusters conformational ensembles into suitable similarity classes, for which 87 representative conformations were identified [45]. The method uses the root mean square distance (RMSD) distribution of the Cα carbons for each distinct pair of conformations. In our case, such distribution has a Gaussian-like shape, with its maximum at 21 Å (Figure S1a). A “proximity score” is then defined as the number of conformations within a RMSD cutoff, which is self-consistently determined during the calculations, from a particular conformer. The conformer with the largest proximity score and the all conformations within the cutoff are then selected and the remaining conformations proceed to the next clustering step. The procedure is repeated until all the conformers are selected. The conformer with the highest proximity score within its cluster is considered the representative of the cluster itself. An optimal cutoff provides few clusters with high proximity score. Based on this criterion, we used here a cutoff of 19 Å. With this choice, the first 6 clusters cover about 75% of the conformations (see the supplementary information: “Cluster analysis S1”).

The seven ligands shown in Figure 1 were docked onto these six representative conformations of AS using AUTODOCK 3.0 [46]. This procedure was used only to generate initial models for subsequent analysis (in several cases, the ligands experience a very high mobility during the dynamics; see results). The parameters for the molecules were calculated using the AUTODOCK standard parameterization procedure [46]. The Lamarckian Genetic Algorithm [46] was applied as a search method for the different docking results. Therefore, 42 complexes were considered. The potential grid map for each atom type was calculated using a cubic box of 252 grid points in each direction, with a distance of 0.5 Å between grid points. For each complex, 100 docking runs were performed resulting in a total of 4,200 calculations. The ligand's location was then identified using a cutoff distance criterion of 5 Å between the Cα carbons of each amino acid and the center of mass of each ligand. The most probable region of interaction for each complex was identified using a cluster analysis based on the RMSD distance among the ligands on each run (RMSD cutoff of 2 Å).

#### Crosschecking of the results

To crosscheck the robustness of our results, we carried out two additional calculations.

1. A different clustering analysis was performed based on a smaller number of starting structures (1,000 randomly selected out from the above ensemble of NMR structures) using a different clustering algorithm. The algorithm was taken from Kelley *et al.* [47]. It is a hierarchical clustering methodology that uses the average linkage algorithm with an automatic determination of the cutoff distance. The algorithm identified 80 clusters or families, with an average spread cutoff of 17.6 ,. Only the first 18 representative conformations (for the first 18 clusters) were taken into account for the analysis as they covered approximately the 50% of the total number of conformations.
2. To provide an additional structure which is largely different from those obtained by NMR, we used molecular dynamics (MD) simulations. This theoretical model was used to test the hypothesis that even the use of a different initial conformation would yield the same result as that obtained using the NMR structures, namely that dopamine binds to AS in a region close to the C-terminal region. Our procedure was as follows: (a) building of an ensemble of AS structural models by performing some preliminary MD simulations in implicit solvent; (b) selection of the AS structure with the largest RMSD value relative to the 6 representative NMR structures; (c) relaxation of this conformer of AS in aqueous solution by MD simulations; (d) construction of the adducts with the ligands in Figure 1 by molecular docking; (e) relaxation of the adducts in aqueous solution by MD simulations (described in the following section).
3.
  - Three 10 ns MD simulations in implicit solvent were performed starting from an extended conformation of the protein (Figure S1b). The simulations differed only in the velocities of the atoms, obtained by three different Maxwell-Boltzmann distributions. The extended conformations were generated by assigning to the φ and ψ backbone angles a value of 180° for all residues except proline (for which φ = −60° and ψ = 180° were used). The side chain geometry of the amino acids was assigned according to the AMBER8's residues templates [48]. The MD simulations in implicit solvent were carried out using the Hawkins, Cramer, Truhlar pairwise generalized Born model [49], [50], as implemented in the AMBER8 program [48], using the AMBER99 [51] force field. The time-step was set to 1 fs. Dielectric constants values of 1.0 and 78.5 were used for the protein and solvent respectively. No ionic strength was assumed. A cutoff distance of 20 Å was used for the electrostatic and van der Waals interactions. The simulations were performed at T = 300 K by coupling the systems with a Langevin thermostat [52], with a collision frequency of 2 ps^−1^. Five hundred AS structures were collected at every 0.02 ns for each of the three MD simulations.
  - These structures were subjected to the cluster analysis as described above [45]. Twenty representative structures, which represent 75% of the structure ensemble, were selected. Among these, the structure of the protein with the largest RMSD value relative to the 6 representative NMR structures was identified. The RMSD between this and the NMR structures ranged between 14 and 21 Å.
  - This structure underwent a MD simulation in water solution in the presence of counter ions. It was inserted into a box of ∼15, 000 water molecules of ∼80×100×100 Å^3^ edges. The overall charges of the system was neutralized by adding 9 Na^+^ ions. Periodic boundary conditions were applied, taking care that the minimum distance between AS and its images was larger than 12 Å. The AMBER99 [51] force field was used for the biomolecule and counter ions, and the TIP3P [53] force field was used for water molecules. Electrostatic interactions were calculated using the particle mesh Ewald method with 64 grid points on each direction and Ewald coefficient of 0.312. A cutoff distance of 10 Å for the real part of the electrostatic and van der Waals interactions was used. The time-step was set to 2 fs. The SHAKE algorithm [54] was applied to fix all bond lengths. The simulations were performed at T = 300 K and P = 1,013 bar by coupling the systems with a Langevin thermostat [52] with a coupling coefficient of 5 ps^−1^, and a Nose-Hoover Langevin barostat [55], with an oscillation period of 200 fs and the damping timescale of 100 fs. The pressure coupling allowed the system to reach a water density of about 0.98 g/cc. Three ns of MD simulation were carried out. The final AS conformation at the end of the MD simulation turned out to be structurally different from the NMR structures: the RMSD ranged again approximately between 14 and 21 Å.
  - Our AS structural model, relaxed in aqueous solution, was selected as a novel, MD-derived conformation of AS for the docking simulations. The seven ligands of Figure 1 were docked into this final MD-derived structure as described in the previous section. Thus, an additional 7 seven complexes were considered and an additional 700 docking simulations were performed (7 ligands×100 docking simulations).

#### MD of AS•dopamine complexes in aqueous solution

The MD simulations of the AS•dopamine NMR-based complexes as well as those of our theoretical models of the complexes were carried out exactly as described above and for a 6 ns time-scale; the only differences being (i) the presence of the ligands and (ii) the number of counter ions (in the case where the ligand was DOP-H the number of ions was 8). For the parameterization, the RESP atomic charges [56] for each ligand were calculated at the HF/6-31G* level of theory, using the GAUSSIAN98 suite of programs [57] (Table S1). The atom types and parameters (i.e. van der Waals, bond lengths, valence angles and dihedral angles) were assigned according to the AMBER99 force field building procedure [48]. The RMSDs and radii of gyration were calculated as described by McLachlan and Allen MP *et al.* [58], [59]. The Tanimoto coefficients for the electrostatic potential (Te) and shape (Ts) of the ligands [60] were calculated using the EON code for chemical similarity analysis [61]. All the MD simulations were performed using the NAMD program [62] and the obtained results were analyzed using Gromacs [63] and VMD programs [64].

The electrostatic interaction energies between residues of the NAC region and the ligands were calculated using two approaches which use the RESP atomic charges [56]. The first is a simple point charge (PC) model *in vacuo*, assuming a dielectric constant of 1 (see Guidoni *et al.* [65]). The second is a finite-difference method (Poisson-Boltzmann). For this, the APBS program [66] was used. The temperature was set to 298K. The solvent and protein dielectric constants were set to 78 and to 2, respectively. The SASA-based apolar coefficient [67] (surface tension) was set to 0.105 kJ/mol/Å and the ionic strength to 0.1.

In both methods, all charges other than the ligands atoms and the atoms in the specific residue in the NAC region were turned off. The electrostatic interaction energies were calculated during the dynamics at every 60 ps. We considered only the residues which were found within 12 Å from the ligands (the distance is measured between the center of mass of each residue in the NAC region and the center of mass of the ligands) for at least 80% of the dynamics. The averages values were then calculated. These methodologies were used here only to provide qualitative results.

### In vitro experiments

#### Cloning, expression and purification of α-synuclein and its mutants

Human wild-type (WT) AS cDNA was cloned into the bacterial expression vector pET-11a at the NdeI site. The mutants were generated using site-directed mutagenesis employing mutagenic primers and two-steps PCR. All constructs were confirmed by DNA sequencing. *E. coli* BL21 cells were transformed with the WT and mutant AS constructs. One bacterial colony was inoculated into 5 ml SOC broth containing 70 µg/ml ampicillin (Q-Biogene, Serva) and incubated overnight at 37°C with continuous shaking. Overexpression of the protein was achieved by transferring 2.5 ml of the pre-culture to 500 ml LB medium supplemented with 70 µg/ml ampicillin. The cells were grown at 37°C, with continuous shaking to an OD at 600 nm of about 0.4–0.6 followed by induction with 1 mM isopropyl-β-thiogalactopyranoside (IPTG) for 3 hrs. After induction, the cells were harvested by centrifugation at 5000 g for 10 min and stored at −20°C. The cell pellet was re-dissolved in 50 mM Tris (Applichem), 50 mM KCl (Applichem), 5 mM MgAc (Applichem), 0.1% Sodium Azide (Applichem), pH 8.5 (1 ml buffer/200 mg pellet). The cell suspension was sonicated for 10 minutes, and the lysate was centrifuged at 8000 g for 30 min. The supernatant was separated from the pellet and the former was first boiled for 20 min, then centrifuged at 8000 g for 30 min, and finally filtered through a 0.22 m filter (Millipore). The protein was firstly purified through anion exchange chromatography (HiPrep Q FF column, Amersham) in 20 mM Tris, pH 8.0/ 20 mM Tris, 1 M NaCl, pH 8.0 followed by injection onto a size exclusion chromatography column (Superdex 200 10/300 or Superdex 200 16/60, Amersham) in 50 mM Tris/HCl, pH 7.5. Purified preparations were dialyzed against water for approximately 24 hrs, then lyophilized and stored at −20°C until use.

#### Fibrillization studies of WT and mutant α-synuclein

To characterize the aggregation properties of WT and mutant AS, proteins were dissolved in 20 mM Tris (Aldrich)/150 mM sodium chloride (Aldrich) pH 7.4, at a concentration of 100 µM. The concentration of AS was determined using its molar extinction coefficient at 280 nm (i.e. ε_280_ = 5120) on a Cary 100 Bio spectrophotometer. The purified proteins were then subjected to fibrillization conditions in absence or presence of an equimolar quantity of dopamine hydrochloride (Fluka) at 37 °C with continuous shaking for the indicated time points.

#### Thioflavin-T (ThT) assay

Fibril formation was monitored by the ThT assay, which was performed by combining 10 µl of aggregated AS with 80 µl Glycine-NaOH (Fluka) pH 8.5, and 10 µl of 100 µM Thioflavin-T (Sigma) in water. Fluorescence measurements were recorded in an Analyst Fluorescence instrument (LJL Biosystems). The excitation and emission wavelengths were set at 450 nm and 485 nm, respectively. The relative fluorescence at 485 nm was used as a measure of the amount of fibrillar aggregates formed in solution.

#### Circular Dichroism (CD)

The average secondary structure of WT and mutant AS was determined by CD spectroscopy using a Jasco J-815 Spectrometer (Omnilab). The Far UV-CD spectra (195–250 nm, integration time of 2 seconds for 0.2 nm) were collected at RT in a 1 mm path length quartz cuvette containing a 1∶5 dilution in water of the samples (WT or mutant AS, concentration 100 µM at time 0) subjected to assembly conditions (72 hrs at 37°C with shaking) in absence or presence of 100 µM DOP.

#### Gel Electrophoresis (SDS-PAGE)

The AS samples were filtered through 0.22 µm PVDF filters, diluted in loading buffer [4% (w/v) Sodium dodecyl sulphate (Fluka), 60 mM Tris, pH 6.8, 10% (v/v) Glycerol (Fluka), 5% (v/v) β-mercaptoethanol (Fluka), 8% (w/v) bromophenol blue, 45.8% (v/v) distilled water] and separated on 12% SDS [(31.3% (v/v) Acrylamide N-N′-methylenebisacrylamide 37,5∶1 solution (Fluka), 25% (v/v) 1.5 M Tris, pH 8.8 (Sigma), with 0.4% (v/v) SDS (Sigma), 42.7% (v/v) distilled water, 0.3% (v/v) ammonium persulfate (APS) and 0.12% (v/v) N,N,N′,N′-tetramethylenediamine, (TEMED)], 1 mm gel. Gels were stained with Simply Blue safe stain (Invitrogen) according to manufacturer's instructions.

#### Transmission Electron Microscopy (TEM)

For EM studies WT and mutant AS samples (35 µM) were deposited on Formvar-coated 200 mesh copper grids (Electron Microscopy Sciences). Grids were washed with two drops of water and stained with two drops of freshly prepared 0.75% (w/v) uranyl acetate (Electron microscopy sciences). Specimens were inspected on a Philip CIME 12 electron microscope, operated at 80 kV. Digitized photographs were recorded with a slow scan CCD camera (Gatan, Model 679).

## Results

### Dopamine•AS structural models

A set of 6 structures representing about 75% of the total number of conformers was selected from the NMR ensemble of ∼3000 structures using a cluster methodology (see Materials and Methods). The adducts with dopamine (DOP) and its derivatives (Figure 1) were constructed by molecular docking (4,200 complexes). The ligands, which were allowed to be flexible, were docked onto the entire protein (considered rigid) and then, for individual conformations with each ligand, the adduct with the ligand in the most probable region of interaction was selected for subsequent analysis (see Materials and Methods for details). Due to the limitations of this procedure for an unstructured protein [68], these calculations are meant only to build up initial structural models for the subsequent MD simulations. Crosschecks were made to ensure that the choice of the initial structure is not critical for the results (see next Section). The ligands' positions turned out to be rather spread along the protein (Table S2 and Figure S2).

In the MD simulations of the resulting 42 adducts in aqueous solution, the ligands formed stable contacts with AS in 60% of the cases (Table S3). We refer to these as ‘stable’ adducts. Their RMSD and radius of gyrations (Rg) appeared to fluctuate around an average value after 3 ns, albeit with rather large values (Table S4). This suggested that the complexes are reasonably equilibrated in spite of the large conformational flexibility.

**Figure 2:**
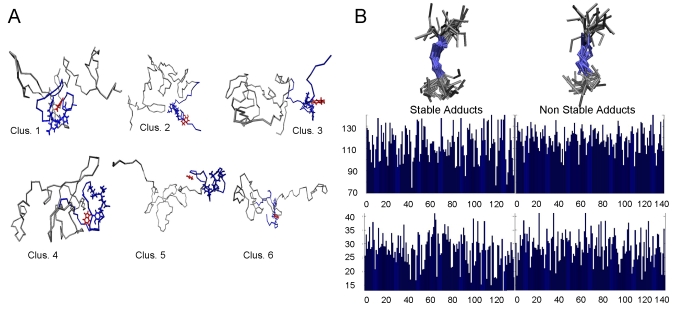
Molecular dynamics simulations. (A) The six most representative conformations of AS, as obtained by the cluster procedure (see Material and Methods) in complex with some of the ligands (in red color). The picture has been obtained after 6 ns MD simulations. In all circumstances, the 125–129 residues (blue licorice) are in contact with the ligands (all the resulting complexes are shown in Figure S3). (B) The 125-126-129 Cα angles show the smaller spread around its average value on all the stable complexes. *Top*. Conformation of the C-terminal region in the stable and unstable adducts. *Middle:* Average values of angles formed by Cα (n−n+1−n+4) on stable (left) and unstable (right) adducts. *Bottom:* standard deviation of those angles (the average is 28° for the stable adducts and 29° for the unstable adducts).

In as much as 73% of the ensemble of the stable adducts, the binding region included the ^125^YEMPS^129^ residues in the C-terminal region (Figure 2A and Table 1); specifically, it involved hydrophobic interactions between the aromatic ring of the ligand and hydrophobic side chains of AS (Table 1 shows the interactions and additional information is presented in Table S5 and Figures S3–S4). In addition, the O and N groups of the ligands formed, in some cases, hydrogen bonds (H-bonds) with polar groups in the same region of AS. The similar binding mode is paralleled by the remarkable structural and electrostatic similarity between the ligands, which is pointed out by the calculated Tanimoto coefficients for the electrostatic potential (Te) and shape (Ts) [60] between each of them (values ranging between 0.7±0.3 and 0.8±0.1 for Te and Ts, respectively, see Table S6). In conclusion, the ligands shared similar binding modes in which the most frequent contacts involve the ligands' aromatic ring moieties.

**Table 1:** H-bond and hydrophobic contacts in Dopamine•AS ‘stable’ adducts.

		Hydrogen Bonds	Hydrophobic contacts
Repr. Cluster 1 (39%)	DCH	Met127(O)-DCH(N1), (D: 3.7±0.8 Å)	Tyr125, (D: 4.7±0.6 Å)
		Ser129(O)-DCH(O1), (D: 4.2±0.5 Å)	Tyr136, (D: 6.4±0.6 Å)
	DHI	Glu137(OE2)-DHI(N1), (D: 2.8±0.2 Å)	Lys80, (D: 4.0±0.3 Å)
			Asp135, (D: 7.0±0.3 Å)
			Tyr136, (D: 9.9±0.5 Å)
	DOP		Lys96, (D: 6.2±0.7 Å)
			Val118, (D: 6.0±09 Å)
			Pro120, (D: 5.4±0.5 Å)
	DOP-H	Glu123(OE2)-DOP-H(O1), (D: 3.5±1.2 Å)	
		Glu123(OE2)-DOP-H(O2), (D: 3.0±0.6 Å)	
	DQ	Asp135(N)-DQ(O2), (D: 3.3±0.4 Å)	Ile112, (D: 6.6±0.7 Å)
		Gly111(O)-DQ(N1), (D: 3.9±1.0 Å)	Asp135, (D: 5.4±1.0 Å)
	IQ		Thr81, (D: 4.0±0.2 Å)
	LEUK		Glu131, (D: 7.4±2.2 Å)
			Gly132, (D: 5.8±2.1 Å)
Repr. Cluster 2 (15%)	DOP-H	Ser129(O)-DOP-H(N1), (D: 3.7±0.9 Å)	Ser129, (D: 5.4±0.7 Å)
		Glu131(O)-DOP-H(N1), (D: 2.8±0.2 Å)	
Repr. Cluster 3 (7%)	DOP		Met127, (D: 7.8±2.4 Å)
	DQ	Thr81(N)-DQ(O2), (D: 4.6±0.5 Å)	Lys34, (D: 6.9±0.7 Å)
	IQ		Lys34, (D: 5.9±0.8 Å)
Repr. Cluster 4 (6%)	DCH	Ala90(N)-DCH(O1), (D: 3.3±0.7 Å)	Phe94, (D: 4.5±0.3 Å)
		Lys97(NZ)-DCH(O2), (D: 3.0±0.5 Å)	Val118, (D: 5.7±0.6 Å)
			Tyr136 (D: 6.3±0.5 Å)
	DHI		Ala90, (D: 4.8±0.3 Å)
			Phe94, (D: 6.5±0.5 Å)
			Lys97, (D: 4.9±0.3 Å)
	DOP	Gly68(N)-DOP(O1), (D: 3.4±0.4 Å)	Gly67, (D: 4.8±0.6 Å)
			His50, (D: 5.0±0.5 Å)
			Val66, (D: 6.2±0.5 Å)
	DOP-H	Thr92(O)-DOP-H(N1), (D: 3.5±0.8 Å)	Tyr125, (D: 5.5±0.7 Å)
	DQ	Gln134(NE2)-DQ(O2), (D: 3.5±0.5 Å)	Tyr39, (D: 4.7±0.3 Å)
			Val49, (D: 6.3±0.5 Å)
	IQ	Glu123(OE2)-IQ(N1), (D: 3.2±0.8 Å)	Phe94, (D: 5.4±0.6 Å)
			Met116, (D: 5.5±0.7 Å)
Repr. Cluster 5 (4%)	DCH	Glu105(OE2)-DCH(N1), (D: 4.0±0.7 Å)	Asp115, (D: 4.8±0.4 Å)
		Met116(O)-DCH(N1), (D: 3.5±0.4 Å)	Pro117, (D: 6.6±0.5 Å)
	DOP-H	Glu105(OE2)-DOP-H(O1), (D: 2.5±0.1 Å)	Val118, (D: 6.0±0.6 Å)
	IQ		Tyr125, (D: 6.2±1.9 Å)
Repr. Cluster 6 (4%)	DCH		Ser129, (D: 6.3±1.2 Å)
			Tyr133, (D: 5.7±1.4 Å)
	DHI		Gly41, (D: 6.4±0.8 Å)
			Pro128, (D: 4.8±0.6 Å)
	DOP	Ala89(O)-DOP(O1), (D: 3.5±0.9 Å)	Leu113, (D: 6.0±0.7 Å)
			Asp135, (D: 6.3±0.4 Å)
	DOP-H	Asp98(OD1)-DOP-H(N1), (D: 2.9±0.4 Å)	Asn65, (D: 5.4±0.6 Å)
			Glu61, (D: 5.5±0.2 Å)
	DQ	Ala89(O)-DQ(N1), (D: 3.3±0.5 Å)	Ala90, (D: 5.8±0.5 Å)
			Ser129, (D: 6.3±1.4 Å)
			Tyr133, (D: 6.1±1.3 Å)
	IQ		Ala56, (D: 5.5±1.0 Å)
			Glu57, (D: 5.5±0.9 Å
MD Derived	DCH		Ala90, (D: 6.7±1.8 Å)
	DHI		Glu83, (D: 12.1±1.5 Å)
			Ile88, (D: 5.1±0.5 Å)
	DOP	Glu131(OE2)-DOP(O1), (D: 3.7±0.9 Å)	Gln134, (D: 8.4±1.5 Å)
	DOP-H	Glu110(OE2)-DOPH(O1), (D: 2.6±0.4 Å)	Gly111, (D: 4.9±1.8 Å)
		Glu114(OE2)-DOPH(N1), (D: 2.9±0.5 Å)	
	DQ	Asn122(ND2)-DQ(O1), (D: 3.9±0.8 Å)	Asp121, (D: 6.5±1.1 Å)

Several of then shown interactions with the C-terminal region, including the ^125^YEMPS^129^ region.

The distance (D) in the hydrogen bond column was measured between the heavy atoms. The hydrophobic contacts were measured as the distance (D) between the center of mass of the ligand and the specific amino acid.

A visual inspection of MD snapshots suggested that the C-terminal binding region assumes a relatively ordered conformation upon binding of the ligands (Figure 2B). We quantified this property by calculating the root mean square fluctuations (RMSF's) and the standard deviation (SD's) of the angles involving Cα carbon atoms. We found it convenient, in particular, to focus on the n, n+1, n+4 angles. In all of the stable complexes, (i) the RSMF's of the C-terminal residues are smaller than those of the N-terminal residues (Figure S5); (ii) the smallest values of the SD's of the angles (n, n+1, n+4) were those of the C-terminal binding region: the SD of the angle 125-126-129 being 13°; compared with the value of 28° averaged over all the other angles (Figure 2B) and with those of the correspondent angles in the unstable complexes (average 29°, Figure 2B). For the rest of the protein, we noticed that the ligands spent a significant amount of the simulated time in proximity to the NAC residues (Figure S6), although they never formed a direct contact with them. According to electrostatic calculations based on a simple point charge model, E83 within the NAC region contributed the most to the long-range electrostatic stabilization of the ligands (at least 30% larger than any other residue in the region, Figure S6). Poisson-Boltzmann calculations provided qualitatively the same results, namely that E83 is the residue in the NAC region forming the largest electrostatic interactions with the ligand (Figure S6) (we further notice that the point charge model provides larger values than those obtained for Poisson-Boltzmann calculations; one has to keep in mind that these calculations are very approximate and cannot be used for quantitative predictions). Test calculations suggested that nullifying E83 charge results in a large decrease of such interactions (∼60%). Within the limitations of our simple analysis, which is used here only for qualitative comparisons, we suggest that additional stabilization of dopamine in its adducts with AS may arise from electrostatic interactions with E83.

In all the remaining simulations, the ligands did not bind in a stable manner with the protein (Table S3) and the structures appeared not to be equilibrated in the timescale investigated. For example, in one case the DOP ligand moved from its starting binding region (residues 92, 93 and 103) towards residues 99, 105 and 106 after 3 ns, and did not form stable interactions with the protein during the time scale of the MD simulation (animation available at http://people.sissa.it/herrera/AS/animations). This was also the case for unbound protein, preventing comparisons between this and the ‘stable’ complex.

### Crosscheck of our results

Although our conformers are representative of most of the AS structural ensemble emerging from NMR, our results may be dependent on the fact that we use a large, but certainly not exhaustive, ensemble of adducts. In this section, we address this issue by performing two additional calculations, as described in the Materials and Methods section.

1. We tested whether starting from a very different structure from those based on NMR would yield similar results. To obtain such a structure, we performed MD calculations *without* any input from experimental data. We selected a model with the largest RMSD relative to the NMR structure (see Materials and Methods for details). Docking the ligands to this structure provided highly different models from those obtained starting from the NMR structures (RMSD ranging from 14 to 20 Å). However, the ligands did bind again to the C-terminal region, in a manner similar to how they did to the NMR structures (see Table 1 and Table S5). Moreover, they were stabilized, also in this case, by electrostatic interactions with E83 (Figure S6). The RMSD and the Rg fluctuated also in this case around an average value after 3 ns (Table S4). Thus, also in these models, the ligands do interact close to the C-terminal region (including residues ^125^YEMPS^129^) and they form long-range electrostatic interactions with E83, which is consistent with what we observed using structures based on NMR studies.
  We notice that the theoretical adducts are not representatives of the entire ensemble of structures that could be obtained by MD simulations. In particularly, they will be different if we change the initial structure and/or the simulation time length. However, our goal here is to use molecular dynamics to provide one additional structure which is largely different from those obtained by NMR representatives, rather than providing an additional ensemble of theoretically built (and therefore less reliable) models. The MD simulations are therefore used here to test whether the results obtained using the NMR representative structures can be obtained using dramatically different structures derived from MD simulations.
2. Next, we repeated the entire computational procedure on the NMR structure using a different clustering algorithm [47] on a different number of initial conformers (1,000 conformers randomly chosen among the 3,062 initial ones). For this cluster analysis, a set of 18 representative conformations representing about 50% of the total number of chosen conformers were selected (see the supplementary information: “Cluster analysis S2”). The ligands were docked onto the representative conformations as in the previous analysis. The MD simulations of the AS*•*ligands complexes were also carried out as previously described. The results of these simulations showed that also for these conformations, the ligands bound mostly to the C-terminal region (Figures S7, S8 and S9 and Tables S7–S8) which assumed a relatively ordered conformation upon binding of the ligands (Figure S10). The complexes also reveal an additional electrostatic stabilization mediated by the interactions between the ligands and Glu83 in the NAC region (Figure S11).

These results demonstrate that our model for AS-dopamine interactions is robust and does not depend significantly on the chosen clustering analysis and/or the chosen number of NMR conformations.

We conclude that, no matter from which structure we start from, the ligands bind mostly to the C-terminal part of the protein which includes the ^125^YEMPS^129^ region. This result is consistent with the experimental observation that dopamine binds *in vitro* to this region [38], [40]. In spite of the limitations of the method used here to investigate dopamine binding to AS (especially the timescale and the large, yet surely not exhaustive ensemble of structures investigated here) the ligands appear to recognize the C-terminal region rather independently from its initial conformation. A possible explanation for this fact is offered by the observation that this region contains as many as five proline residues (in contrast to the rest of the protein, which does not contains prolines). In fact, this can impose local restrictions stabilizing the structure of the backbone of this region as it was shown for another member of AS family, β-synuclein, which shares 60% of sequence identity and contains 8 prolines in its C-terminal region [69].

### Testing the structural predictions by in vitro fibrillization studies

On the basis of the above results, we conclude that the ligands form nonspecific hydrophobic interactions with all of the five residues in the ^125^YEMPS^129^ region (Table 1) and form H-bond to E126 and S129 in some cases. In all cases, AS always assumes a similar, kinked conformation in its binding region (Figure 2B). In addition, the ligands may be significantly stabilized by electrostatic interactions with E83. To test the validity of these conclusions, obtained within the limitation of the computational protocol outlined above, we investigated *in vitro* the aggregation properties in the presence and absence of dopamine of four alanine mutants of AS, which involve the ^125^YEMPS^129^ residues in the C-terminal region, as well as E83 in the NAC region (Table 1): E83A, E126A, S129A and E83A/E126A/S19A. Because our calculations suggest that ligand-AS interactions at the C-terminus are dominated by nonspecific hydrophobic interactions, we predict that the E126A and S129A mutations should not significantly alter the ligand-AS interaction. In addition, in the case of S129A, the Ser to Ala mutation might not affect H-bonding with the ligand as it involves the backbone. In contrast, the E83A mutation is expected to affect dopamine affinity for AS (and therefore it might affect the fibrillization process).

**Figure 3:**
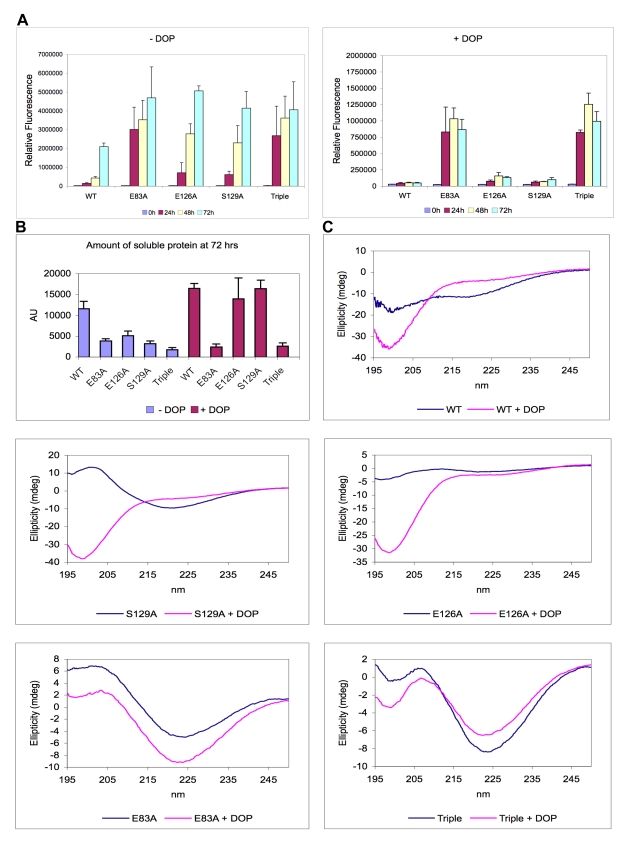
*In vitro* fibrillization of α-synuclein. (A) Kinetics of fibrillization of WT and mutant (E83A, E126A, S129A and Triple) α-synuclein under assembly conditions in absence or presence of an equimolar quantity of dopamine (DOP) as monitored by the enhancement in Thioflavin-T (ThT) fluorescence intensity over time. Data are expressed as the mean±SEM (Standard Error of the Mean) of 2 or 3 independent experiments. (B) Amount of soluble WT and mutant α-synuclein protein remaining in solution after 72 hrs incubation under assembly conditions in absence or presence of an equimolar quantity of DOP monitored by SDS-PAGE. (C) CD spectra of the soluble WT and mutant α-synuclein proteins remaining in solution after 72 hrs incubation under assembly conditions in absence (blue line) or presence (red line) of an equimolar quantity of DOP.

Recombinant human WT, E83A, E126A, S129A and E83A/E126A/S129A AS proteins were expressed and purified as described in the “Materials and Methods” section. The aggregation properties of the five proteins were determined by incubating 100 µM of protein in TRIS buffer (20 mM Tris, 150 mM NaCl, total volume = 500 µl in 1.5 ml plastic tubes) at 37 °C with continuous shaking for 72 hrs in the absence or presence of equimolar dopamine. At regular intervals, aliquots were removed and subjected to analysis by ThT fluorescence assay, SDS-PAGE gel, circular dichroism (CD) and electron microscopy (EM). All the mutants showed increased aggregation relative to the WT (Figure 3A). Interestingly, the two AS variants containing the E83A mutation retained the ability to form fibrils in the presence of an equimolar quantity of dopamine (Figure 3A) whereas fibril formation by the WT, E126A and S129A variants was abolished in the presence of dopamine. This result clearly suggests that the nature of dopamine•AS interactions in the C-terminal region is distinct from that of the interactions in the NAC region, consistent with our predictions. A consistent picture was obtained by monitoring the loss of soluble (monomers, oligomers and protofibrils) protein during the fibrillization reaction by SDS-PAGE. Aliquots of the samples at various time points were diluted in buffer (factor 1∶10) and filtered through 0.22 µm PVDF filters to eliminate fibrillar and insoluble aggregates, before analysis in 12% PAA gels with Coomassie Blue staining. The signal corresponding to the protein in the flow-through (monomer and soluble oligomers) was quantified using the software ImageJ. In agreement with the ThT data, we saw a decrease of soluble content that was proportional to the degree of aggregation of each protein (Figure 3B). In the presence of dopamine, the majority of WT, S129A, and E126A protein remained in solution, whereas very little soluble protein remained in the case of the two E83A-containing mutants (Figure 3B, red rectangles).

The CD spectra showed that all proteins adopt predominantly random coil conformation in solution (data not shown) but form β-sheet rich structures after incubation for 72 hrs at 37°C. In the absence of dopamine and after incubation for 72 hrs, the majority of WT, E126A, and S129A precipitated out of solution and the CD spectra of the remaining material exhibited a predominantly random coil structure, except for S129A which exhibited a spectra consistent with species (soluble aggregates) rich in β-sheet structure (Figure 3C). Co-incubation with dopamine prevented the transition from random coil to β-sheet in the case of WT, S129A, E126A, but not in the case of E83A or E83A/E126A/S129A, further confirming that dopamine is able to prevent the fibrillization of the WT, E126A and S129A mutants but not of the E83A and E83A/E126A/S129A mutants (Figure 3C).

**Figure 4:**
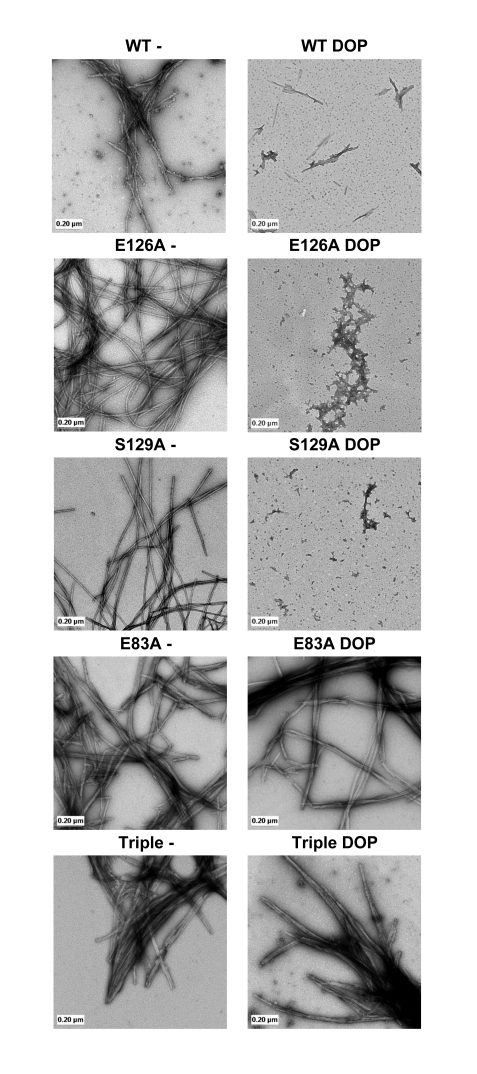
TEM analysis: WT and mutant α-synuclein (E83A, E126A, S129A and Triple) filament assembly in absence or presence of an equimolar quantity of dopamine (DOP). Proteins were incubated under assembly conditions for 72 hrs and analyzed by negative staining EM as described in Materials and Methods.

To characterize the extent of aggregation and the effect of dopamine on the structural properties of the AS aggregates, we performed EM studies on AS samples incubated for 72 hrs in the presence or absence of dopamine. In the absence of dopamine, after 48–72 hrs the mutant proteins showed abundant fibrils resembling those formed by WT AS (Figure 4, left panels). In the presence of dopamine, WT, E126A and S129A formed predominantly soluble aggregates and no mature fibrils could be detected in these samples, which is consistent with previously reported data on the WT protein. On the contrary, addition of dopamine did not inhibit fibril formation or change the structure of the fibrils formed by both E83A containing mutants (Figure 4, right panels).

## Discussion

We have presented both molecular dynamics and *in vitro* biophysical investigations of complexes formed by AS and dopamine and several of its derivatives in aqueous solution. These calculations, which were based on structures representing about 75% of the conformations obtained by NMR spectroscopy [22], suggest that the ligands bind non-covalently to the C-terminal region including the residues ^125^YEMPS^129^. This result is consistent with data obtained by *in vitro* fibrillization studies [38], [40] and the proposed role of the C-terminal region encompassing these residues in inhibiting the aggregation of AS. In all the cases that we investigated, the dopamine-binding region assumed a similar structure (Figure 2B). Moreover, the same results were obtained by applying our computational protocol to theoretical models based on MD simulations of AS in aqueous solution as well as by performing a rather different clustering analysis on the NMR structures. We therefore suggest that, in spite of the limitations of the computational methodology presented, our calculations provide a consistent picture for the structural determinants of the binding region for the non-covalent complexes: the fingerprint of dopamine•AS non-covalent complexes is the formation of nonspecific hydrophobic contacts between the ligands' aromatic ring and the ^125^YEMPS^129^ region in the C-terminus (Table 1), which is aided by the particular conformation adopted by the binding region to accommodate the ligand (Figure 2B). These interactions are also complemented by nonspecific H-bonding interactions. In addition, dopamine and its derivatives are stabilized by significant electrostatic interactions with E83 in the NAC region (Figure S6).

Thus, based on our computational findings, we hypothesized that the ^125^YEMPS^129^-DOP, nonspecific hydrophobic interactions may affect the AS-DOP binding and hence DOP's ability to modulate AS fibrillization. In addition, replacement of E83 with alanine in the NAC region should abolish the favorable long range electrostatic interactions with the ligands upon binding to the C-terminal region. To test this hypothesis, we mutated selected residues involved in dopamine interactions and investigated the *in vitro* aggregation properties of these mutants (E83A, E126A, S129A and the triple mutant E83A/S129A/E126A) using an array of biophysical methods.

Our calculations suggest that hydrophobic interactions with the ^125^YEMPS^129^ C-terminal region play a critical role in the interaction of AS with DOP and its derivatives. We found that single amino acid substitutions (E126A and S129A) in this region do not abolish DOP inhibition of AS fibrillization *in vitro*. Indeed, previous studies have shown that abolishment of DOP inhibition of AS aggregation requires substitution of all the 5 amino acids in the ^125^YEMPS^129^ region (YEMPS to FAAFA) or deletion of amino acids 125–140 [38], [40].

These results suggest, in agreement with our calculations showing nonspecific hydrophobic interactions between the aromatic ring of the ligand and hydrophobic side chains of the C-terminus, that the entire region is important for DOP binding. Interestingly, the E83A mutation in the NAC region strongly impairs the ability of dopamine to inhibit AS aggregation. This mutation may either prevent DOP binding to ^125^YEMPS^129^, which is consistent with our conclusion that dopamine affinity is stabilized by E83 long range electrostatic interactions, or alter some property of the NAC region, which is required for DOP inhibition of AS fibrillization. Therefore, our findings suggest that both the C-terminus and the NAC region are important for the inhibition of AS fibrillization by DOP.

The protocol that we have adopted here, which combines *in silico* and *in vitro* methods, may help devise novel ligands that mimic interactions between dopamine and AS. These molecules may act as potential inhibitors of AS aggregation and provide initial lead structure in developing small molecule therapeutics for PD and related synucleinopathies. In order to explore these opportunities, however, further studies will need to establish whether DOP inhibition of AS aggregation is toxic or protective in neurodegeneration. Further, this type of approach may be extended to other disease-related naturally unfolded proteins.

## Supporting Information

Figure S1 Structural models: A. Clustering of the 3,062 NMR structure selected from NMR experiments: Histogram of the RMSD pair distance matrix. B. MD simulation: Cartoon of the AS conformation obtained by setting the backbone dihedral angles ϕ, ψ = 180° of all residues except proline and ϕ = −60°, ψ = 180° for proline. This structure was used for the MD simulations in implicit solvent. For the sake of clarity, only the backbone atoms are shown.
(0.20 MB TIF)

Figure S2 Molecular docking: Results for the 100 runs for each of the 6 AS representatives conformations with DCH (yellow), DHI (pink), DOP (light blue), DOP-H (blue), DQ (green), IQ (red) and LEUK (black) (54 complexes). Clustering of the doking results, as implemented in AUTODOCK, plotted as a function of the AUTODOCK scoring function (in Kcal/mol). Inset: The number of hits (defined in Table S2) between AS and the respective ligand.
(0.60 MB PDF)

Figure S3 MD simulations of the “stable” DOP/AS complexes. Structures and Cα contacts maps for the last MD snapshots. In the structures, residues 125–129 and E83 are colored in red and blue, respectively. In the contact maps, the x and y axis indicate the residues number in the AS sequence. The contact maps were calculated based on the Cα-Cα distance (a graph square is colored black at 0.0 A distance, to a linear gray scale between 0.0 and 10.0 A, and white when equal to or greater than 10.0 A).
(0.81 MB PDF)

Figure S4 MD simulations of the stable complexes. Ligand/protein interactions are represented using Ligplot program.
(0.92 MB DOC)

Figure S5 Structural fluctuations. Molecular dynamics of the NMR-derived conformations with the ligands. The Root mean square fluctuations (RMSF's, in A) are reported for the 26 stable complexes.
(0.04 MB PDF)

Figure S6 Electrostatic interactions: interactions energies between the ligands and residues in the NAC region, as obtained by a simple point charge model and by Poisson-Boltzmann calculations. These interactions are averaged along our molecular dynamics of the e NMR derived and MD-derived AS•dopamine adducts. Top: Number contacts (defined in Materials and Methods) between NAC residues and the ligands. The residues selected for the electrostatic analysis (see Materials and Methods) are marked in black. Bottom. Averaged energies values for the selected residues normalized to the largest values, as in the work of Guidoni et al. For the point charge model and Poisson Boltzmann calculations, Av = −2.7 Kcal/mol and −0.3 Kcal/mol.
(0.42 MB TIF)

Figure S7 Molecular docking and MD simulations of dopamine and its derivatives onto AS: A) Number of hits (defined in Table S2) between AS and DOP, DOP-H and DCH, as obtained by 5,400 docking runs. B) In 11 simulations out of 18, the ligands bind to the ^125^YEMPS^129^ region. Here we show six of those conformations where the 125–129 residues and E83 are colored in blue, the ligand is colored in red.
(1.39 MB TIF)

Figure S8 MD simulations of the stable DOP-, DOP-H- and DCH-AS complexes. Final structures and contact maps for the last MD snapshots. Black to white scale as in Figure S3. Residues 125–129 and Glu 83 are colored in blue and the ligands are colored in red.
(0.39 MB DOC)

Figure S9 MD simulations in the “stable” complexes from the second cluster analysis. The ligands and the residues involved in the interactions are shown in sticks. Hydrogen bonding and hydrophobic interactions are shown as dashed lines. Snapshots taken from the last frame of the MD simulations.
(0.26 MB PDF)

Figure S10 MD simulations of the NMR-derived conformations from the second cluster analysis. Top: Average values of angles formed by Cα (n−n+1−n+4) on stable (left) and unstable (right) adducts. Bottom: standard deviation of those angles (the average is 30° for the stable adducts and 28° for the unstable adducts).
(5.49 MB TIF)

Figure S11 MD simulations of the NMR-derived conformations from the second cluster analysis.: Ligand/NAC interactions. Left:Number of times that NAC aminoacids are found within a 12 A from the ligands of Figure 1.The residues selected for the electrostatic analysis are marked in black. Right. Averaged energies values (calculated using a point charge model), for the selected residues (Res), normalized to the largest value. The average interaction is −1.4 Kcal/mol.
(0.23 MB TIF)

Table S1 MD simulations. Atoms labeling and RESP atomic charges of the ligands in Figure 1.
(0.12 MB DOC)

Table S2 Molecular Docking: Top) Number of hits between α-synuclein (AS) and the seven ligands as obtained by 4,200 docking runs of Autodock. The hits are defined here when the distance between at least one AS's Cα atom and the ligands' center of mass is lower than 5 A. Bottom) Relative contribution for the binding of the C-terminal regions, calculated as percentages of the total number of ligand-protein contacts
(0.11 MB DOC)

Table S3 Stabilities. The stabilities of the local interactions between the ligand and AS for all MD simulations are reported here.
(0.03 MB DOC)

Table S4 MD simulations. RMSD (A) and radius of gyration (A) of the so-called ‘stable’ adducts.
(0.08 MB DOC)

Table S5 MD simulations of dopamine and its derivatives in complex with AS (49 complexes). Distance between the center of mass of dopamine (and its derivatives reported in Figure 1) and that of residues E83, 110–140. The average values (Av.), along with their standard deviations (SD), are reported.
(0.57 MB DOC)

Table S6 Structural and electrostatic similarity across the ligands reported Figure 1. The Tanimoto coefficients characterizing the shape (Ts) and the electrostatic potential (Te) of the ligands reported in Figure 1 are presented. DOP-H is not shown because it is charged, unlike all of the other ligands.
(0.04 MB DOC)

Table S7 MD simulations of NMR-derived conformations from the cluster analysis of Kelley et al. Hydrogen bonds and hydrophobic contacts for 11 out of the 18 analyzed complexes forming interactions with the protein. Several of then shown interactions with the C-terminal region, including the ^125^YEMPS^129^ region. The distance (D) in the hydrogen bond column was measured between the heavy atoms. The hydrophobic contacts were measured as the distance (D) between the center of mass of the ligand and the specific amino acid.
(0.04 MB DOC)

Table S8 MD simulations of the dopamine forms/AS adducts. The average values (Av.) with their standard deviations (SD) of the distance between the center of mass of E83 along with the C-Terminal residues (from 110 to 140) and dopamine, along the trajectory, are reported here.
(0.25 MB DOC)

Cluster Analysis S1 Cluster Analysis of Micheletti et al.
(0.04 MB DOC)

Cluster Analysis S2 Cluster analysis of Kelley et al.
(0.04 MB DOC)
