# Supplementary figures and images for: Inhibition of α-Synuclein Fibrillization by Dopamine Is Mediated by Interactions with Five C-Terminal Residues and with E83 in the NAC Region
Source: PLoS One. 2008 Oct 14;3(10):e3394. doi: 10.1371/journal.pone.0003394 (PMC2566601; doi:10.1371/journal.pone.0003394)

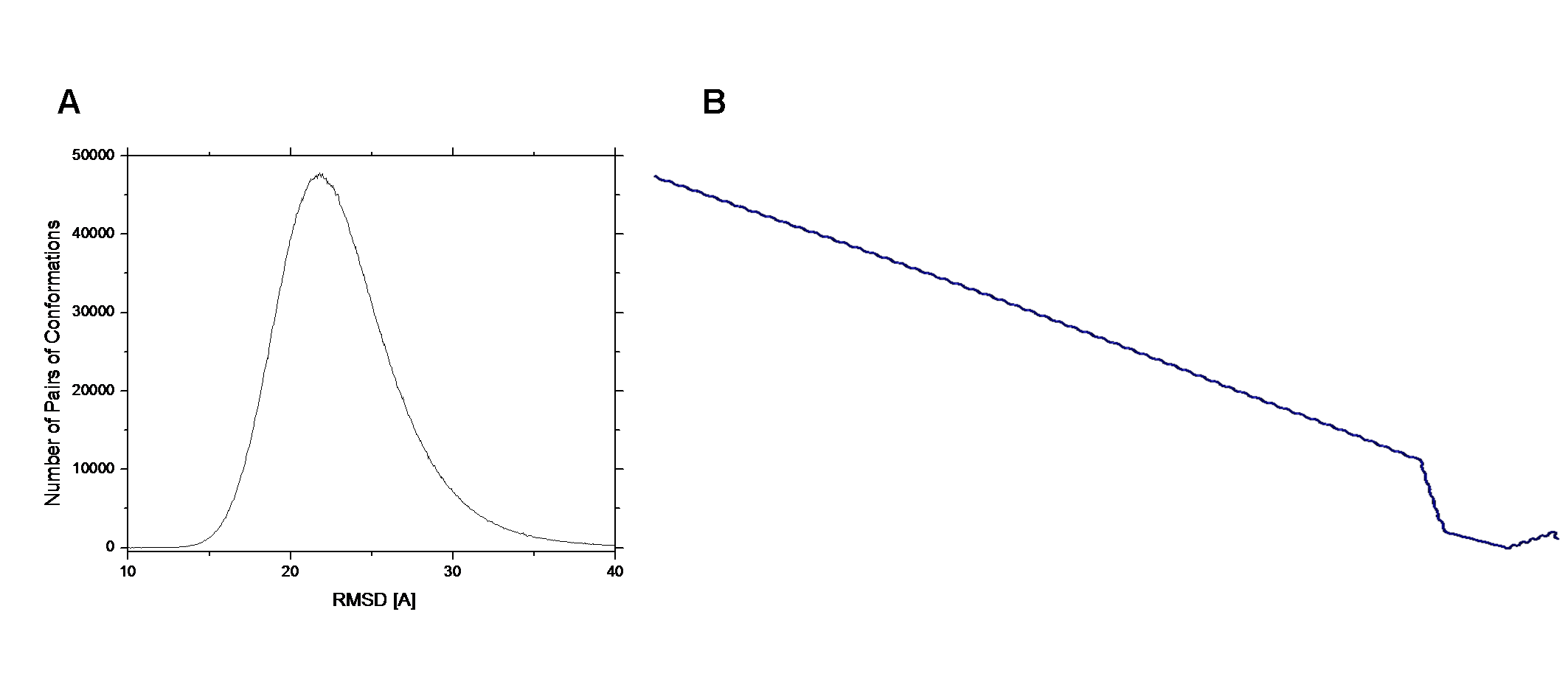

Supplement: Figure S1 — Structural models: A. Clustering of the 3,062 NMR structure selected from NMR experiments: Histogram of the RMSD pair distance matrix. B. MD simulation: Cartoon of the AS conformation obtained by setting the backbone dihedral angles ϕ, ψ = 180° of all residues except proline and ϕ = −60°, ψ = 180° for proline. This structure was used for the MD simulations in implicit solvent. For the sake of clarity, only the backbone atoms are shown. (0.20 MB TIF) [file pone.0003394.s001.tif]

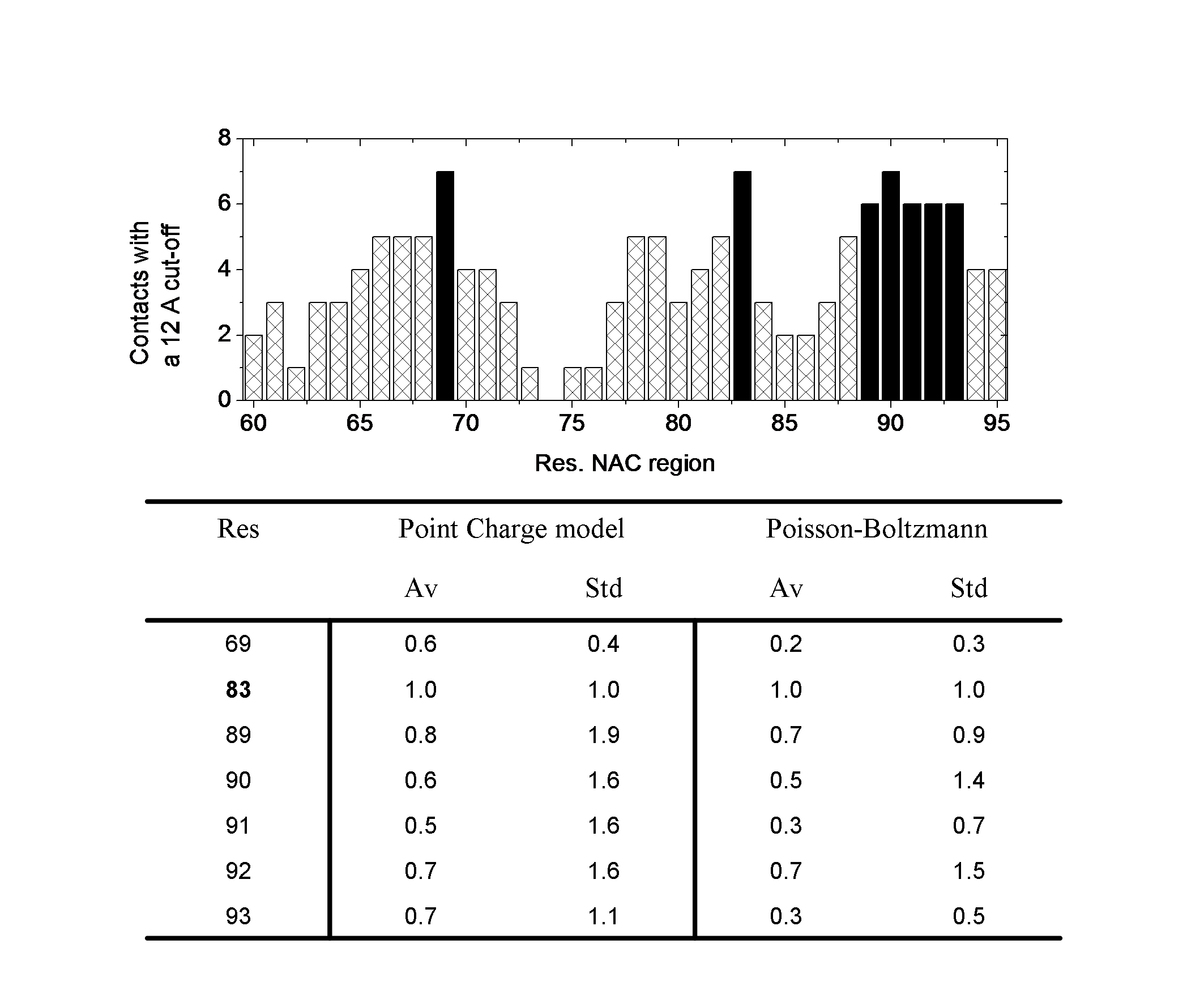

Supplement: Figure S6 — Electrostatic interactions: interactions energies between the ligands and residues in the NAC region, as obtained by a simple point charge model and by Poisson-Boltzmann calculations. These interactions are averaged along our molecular dynamics of the e NMR derived and MD-derived AS•dopamine adducts. Top: Number contacts (defined in Materials and Methods) between NAC residues and the ligands. The residues selected for the electrostatic analysis (see Materials and Methods) are marked in black. Bottom. Averaged energies values for the selected residues normalized to the largest values, as in the work of Guidoni et al. For the point charge model and Poisson Boltzmann calculations, Av = −2.7 Kcal/mol and −0.3 Kcal/mol. (0.42 MB TIF) [file pone.0003394.s006.tif]

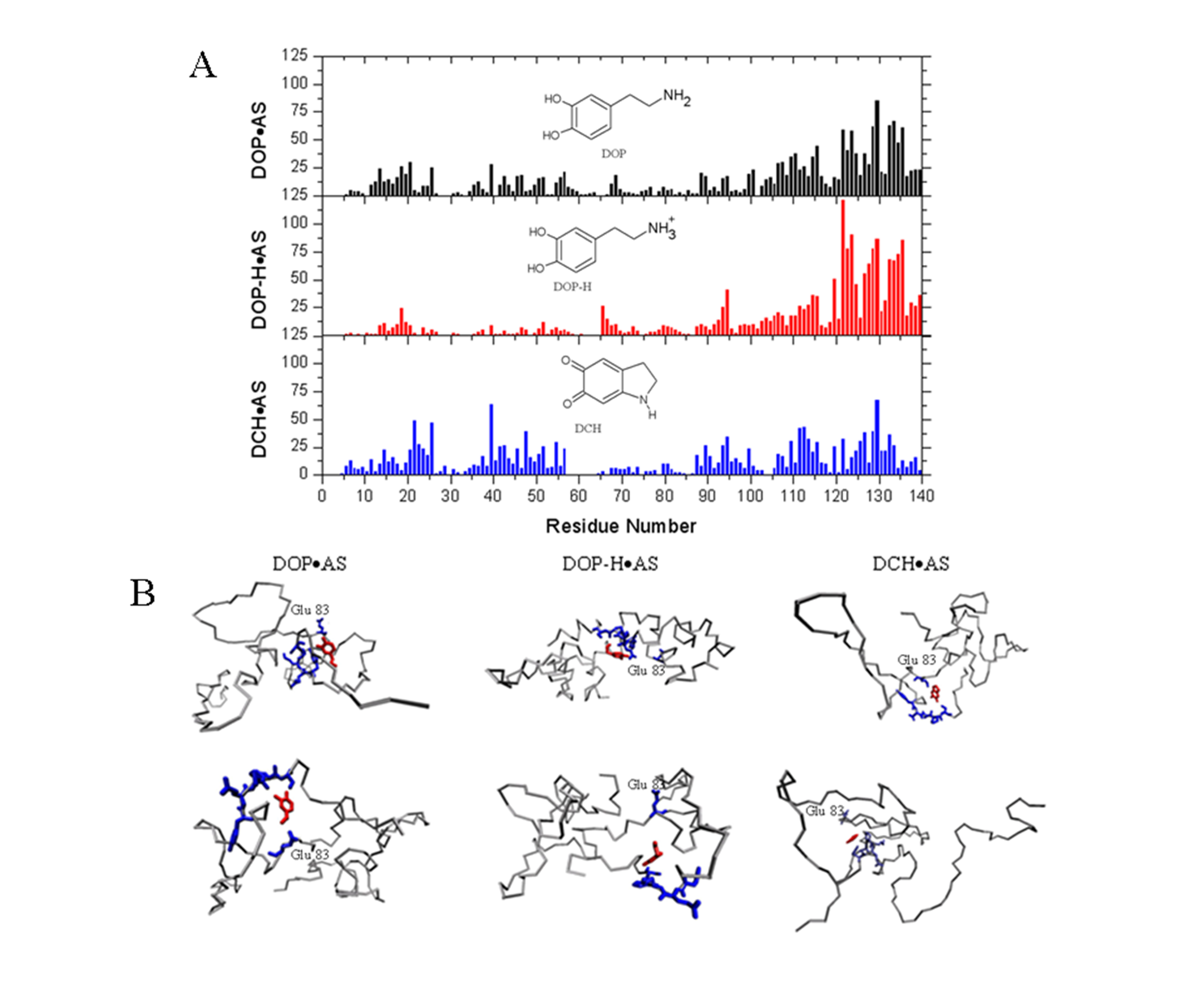

Supplement: Figure S7 — Molecular docking and MD simulations of dopamine and its derivatives onto AS: A) Number of hits (defined in Table S2) between AS and DOP, DOP-H and DCH, as obtained by 5,400 docking runs. B) In 11 simulations out of 18, the ligands bind to the 125YEMPS129 region. Here we show six of those conformations where the 125–129 residues and E83 are colored in blue, the ligand is colored in red. (1.39 MB TIF) [file pone.0003394.s007.tif]

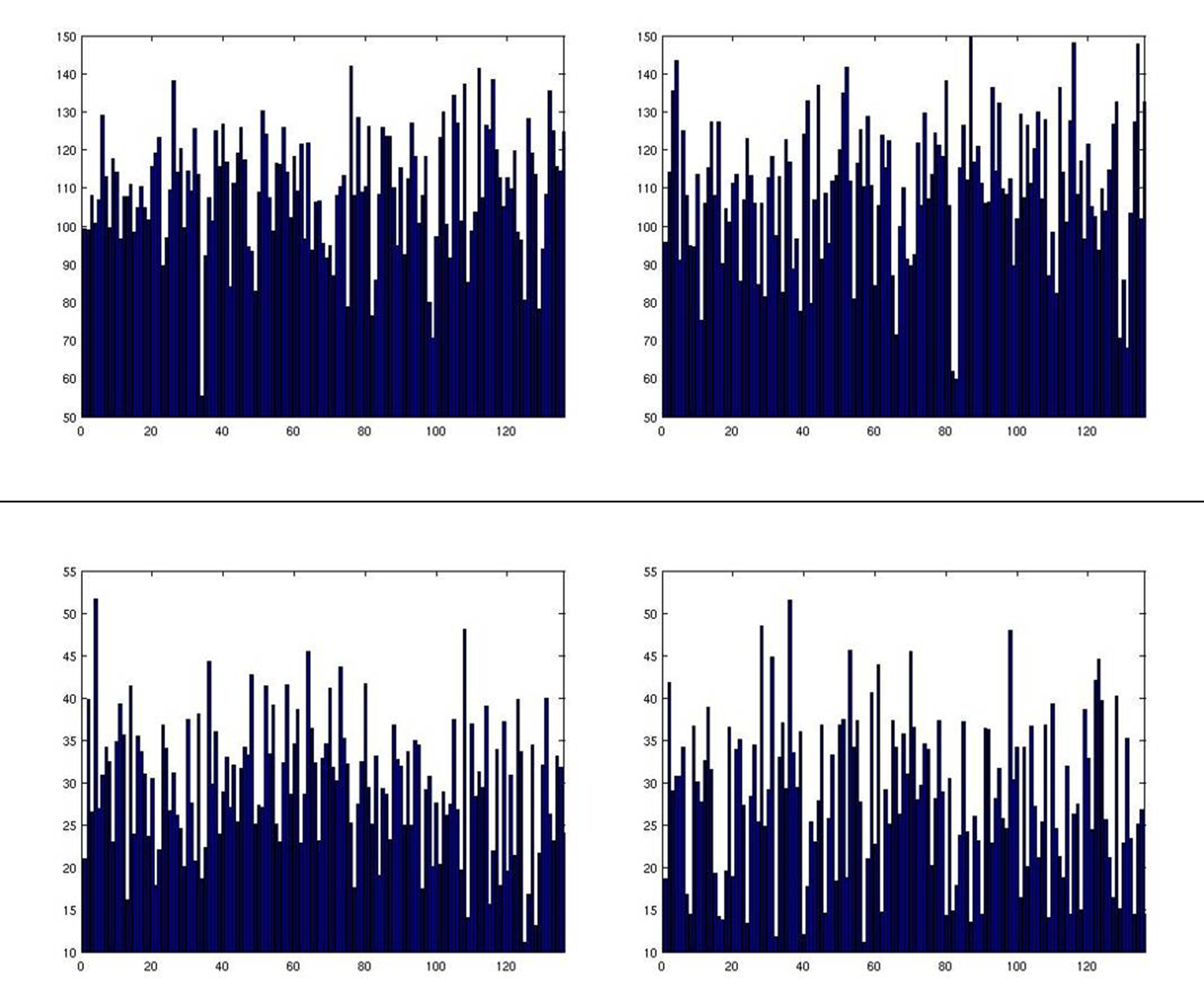

Supplement: Figure S10 — MD simulations of the NMR-derived conformations from the second cluster analysis. Top: Average values of angles formed by Cα (n−n+1−n+4) on stable (left) and unstable (right) adducts. Bottom: standard deviation of those angles (the average is 30° for the stable adducts and 28° for the unstable adducts). (5.49 MB TIF) [file pone.0003394.s010.tif]

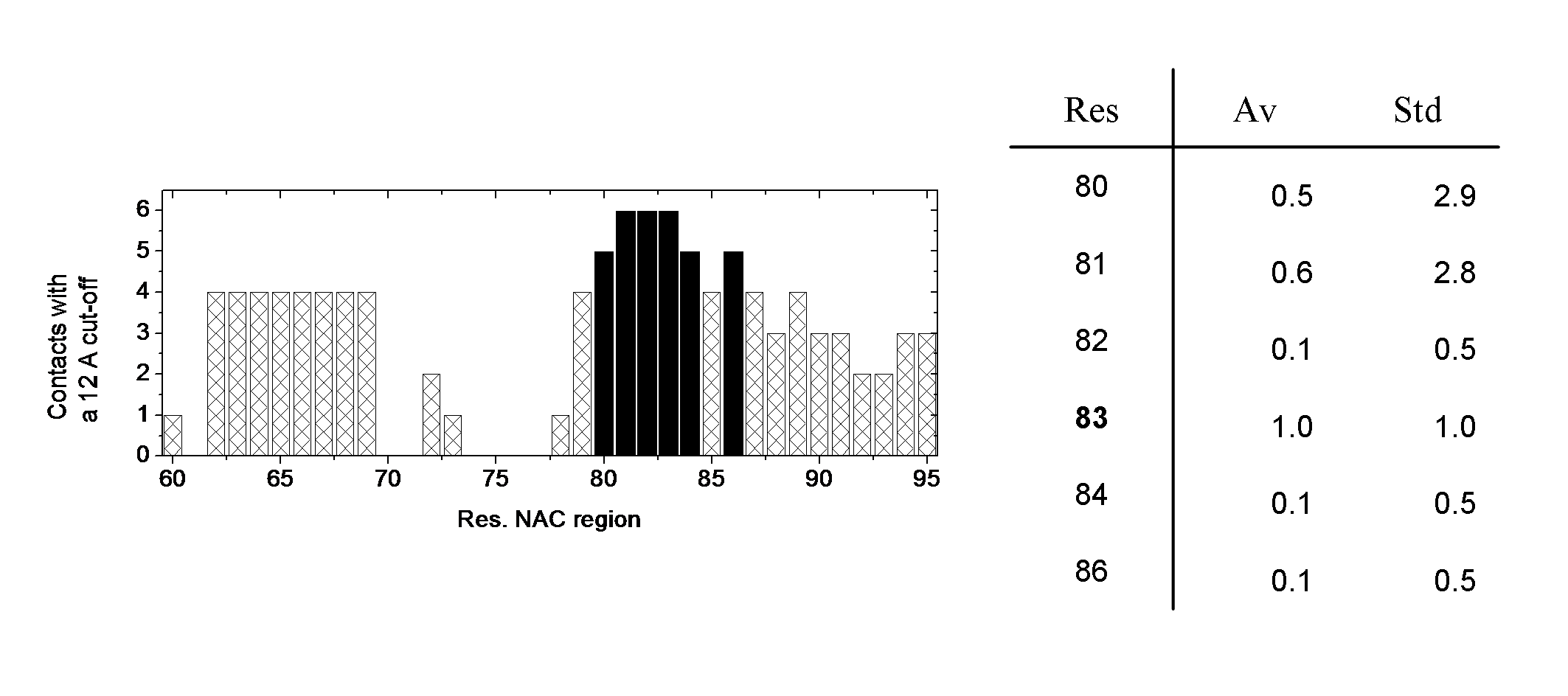

Supplement: Figure S11 — MD simulations of the NMR-derived conformations from the second cluster analysis.: Ligand/NAC interactions. Left:Number of times that NAC aminoacids are found within a 12 A from the ligands of Figure 1.The residues selected for the electrostatic analysis are marked in black. Right. Averaged energies values (calculated using a point charge model), for the selected residues (Res), normalized to the largest value. The average interaction is −1.4 Kcal/mol. (0.23 MB TIF) [file pone.0003394.s011.tif]
