# Supplementary material for: Inhibition of α-Synuclein Fibrillization by Dopamine Is Mediated by Interactions with Five C-Terminal Residues and with E83 in the NAC Region
Source: PLoS One. 2008 Oct 14;3(10):e3394. doi: 10.1371/journal.pone.0003394 (PMC2566601; doi:10.1371/journal.pone.0003394)
Supplement: Figure S2 — Molecular docking: Results for the 100 runs for each of the 6 AS representatives conformations with DCH (yellow), DHI (pink), DOP (light blue), DOP-H (blue), DQ (green), IQ (red) and LEUK (black) (54 complexes). Clustering of the doking results, as implemented in AUTODOCK, plotted as a function of the AUTODOCK scoring function (in Kcal/mol). Inset: The number of hits (defined in Table S2) between AS and the respective ligand. (0.60 MB PDF) [file pone.0003394.s002.pdf]

Cluster 1

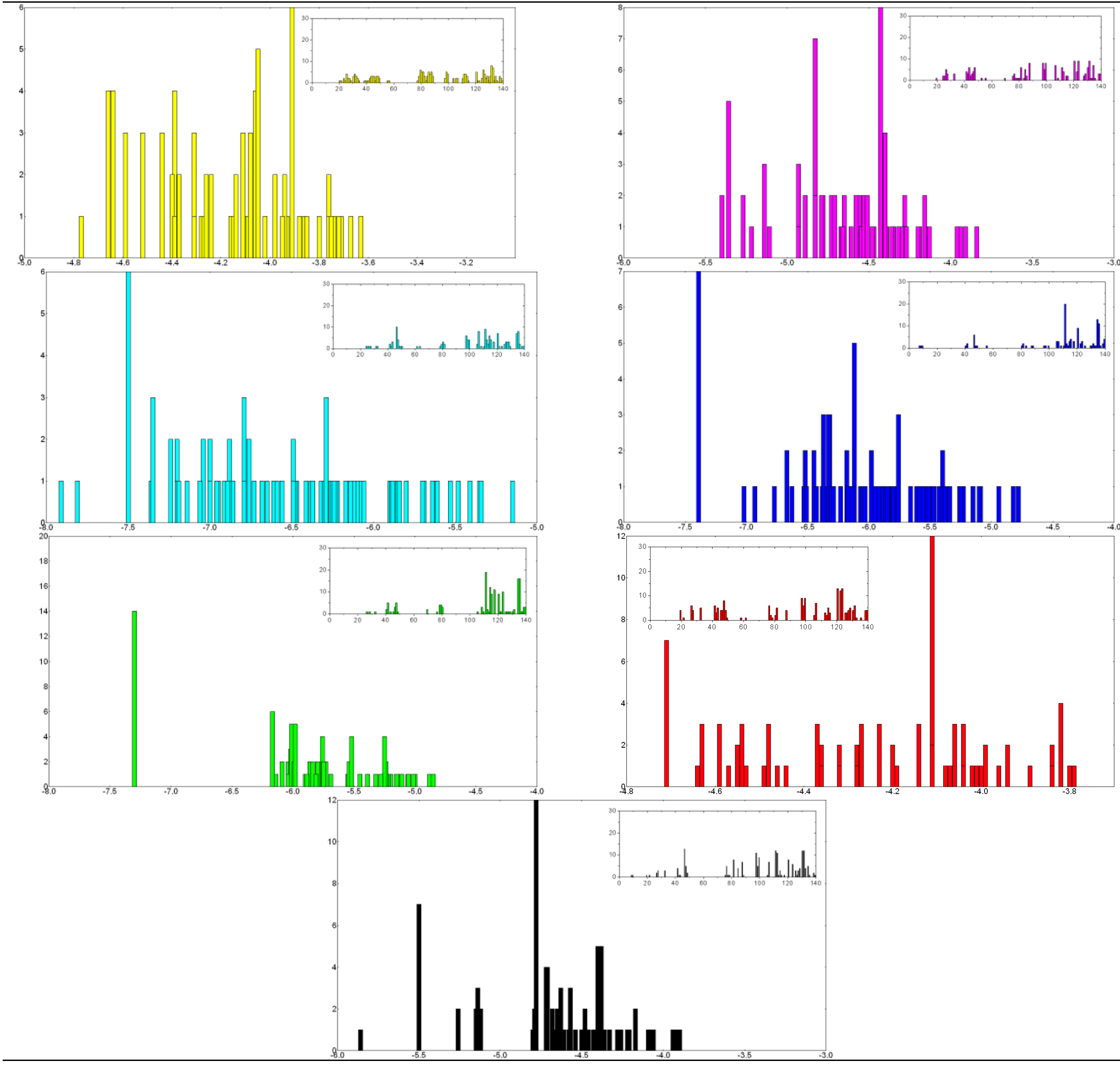

Cluster 2

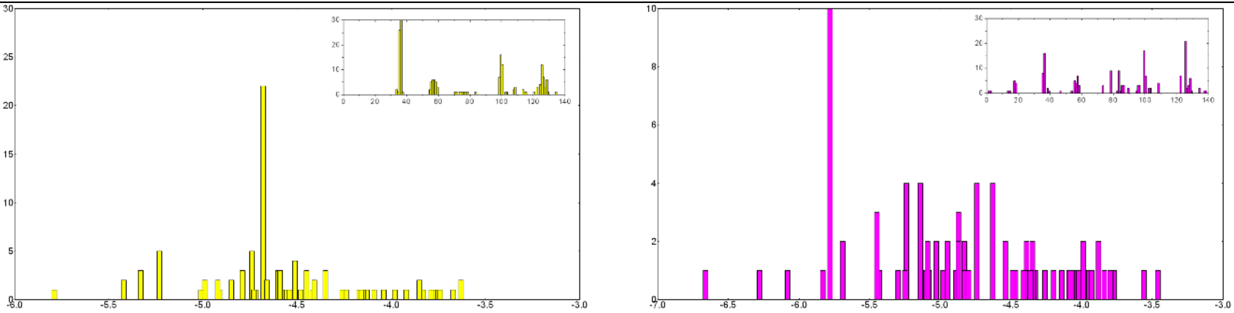

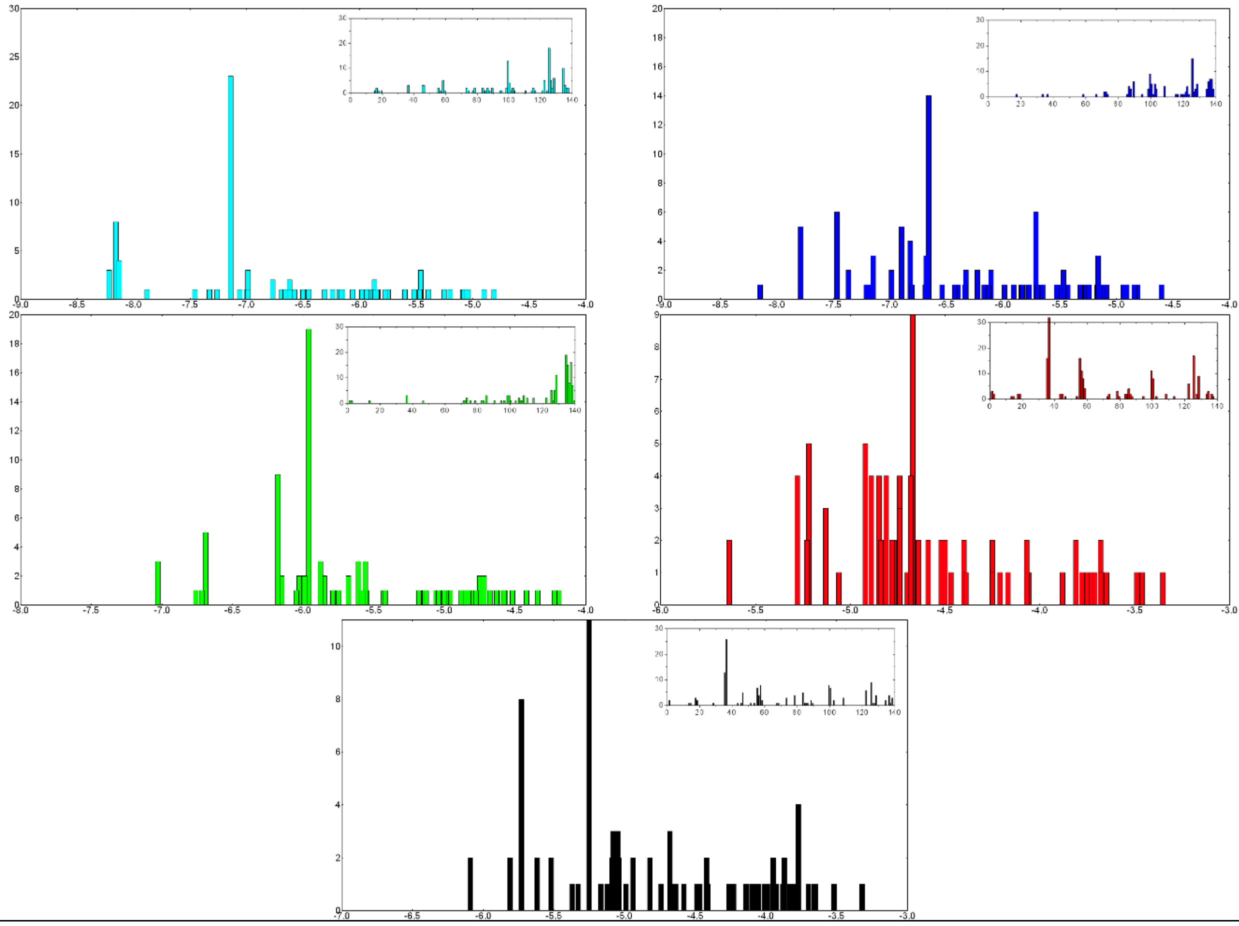

Cluster 3

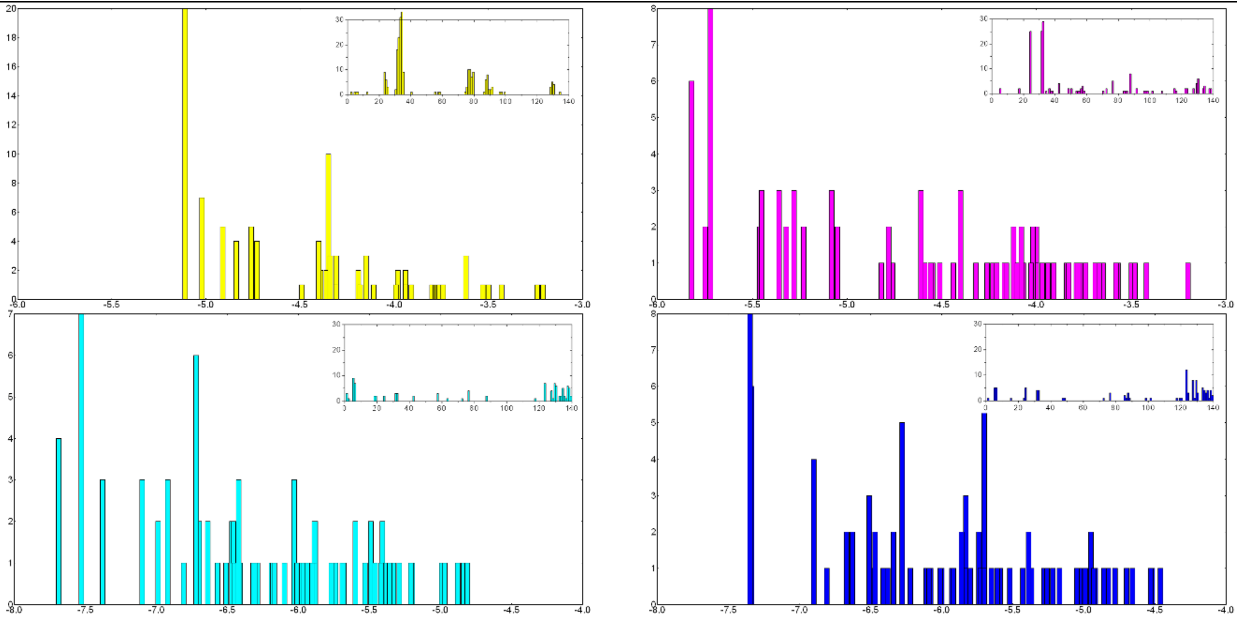

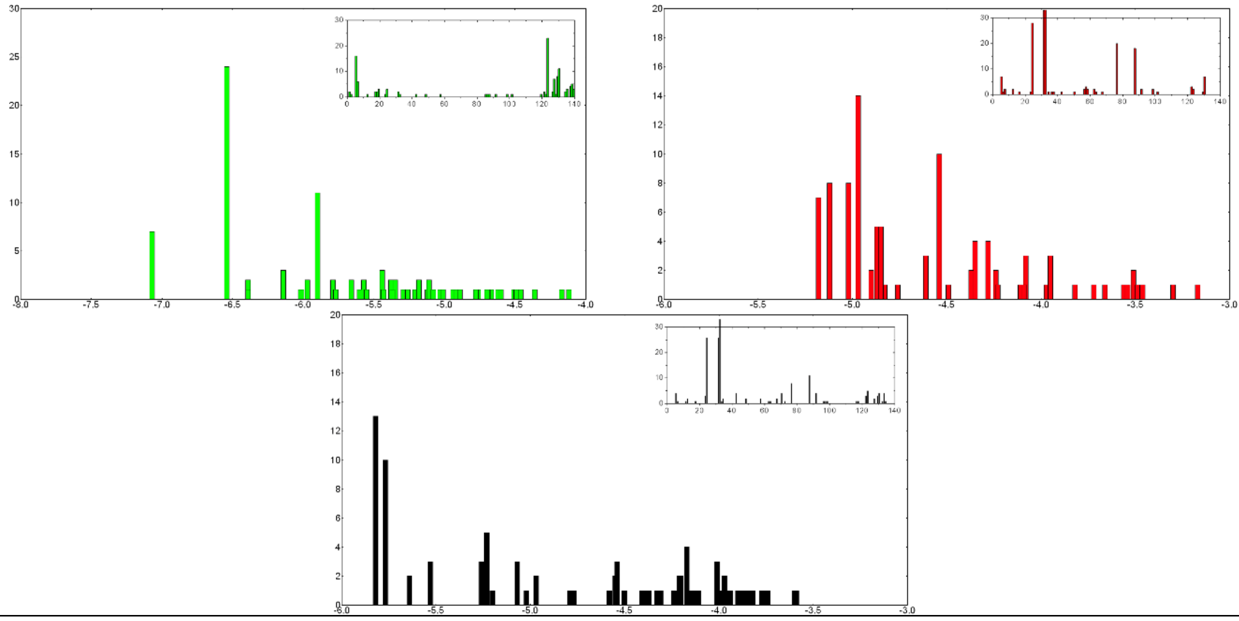

Cluster 4

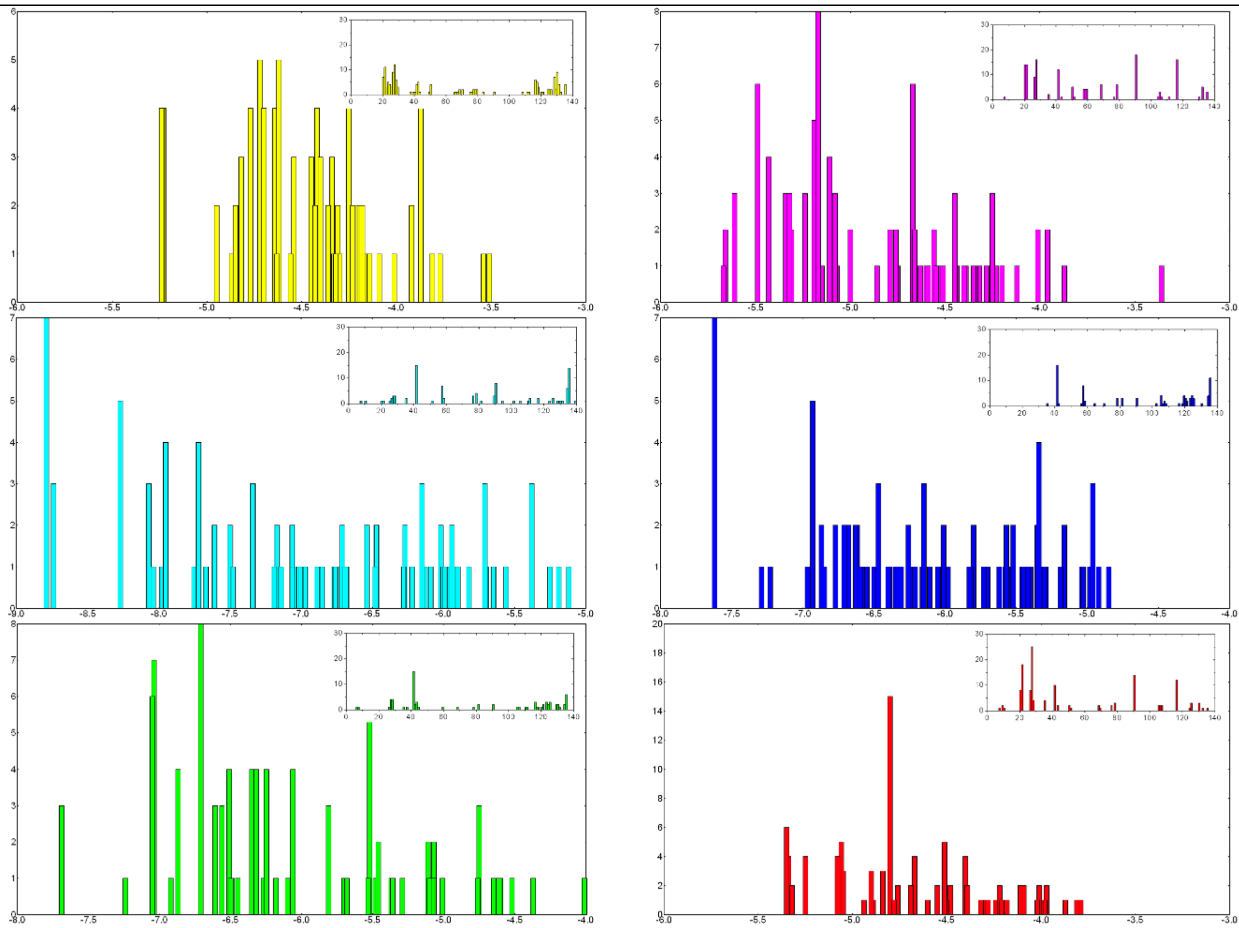

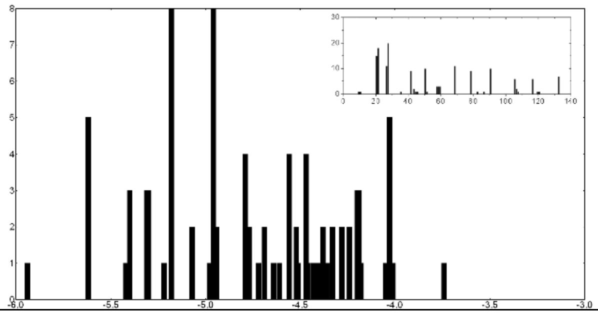

Cluster 5

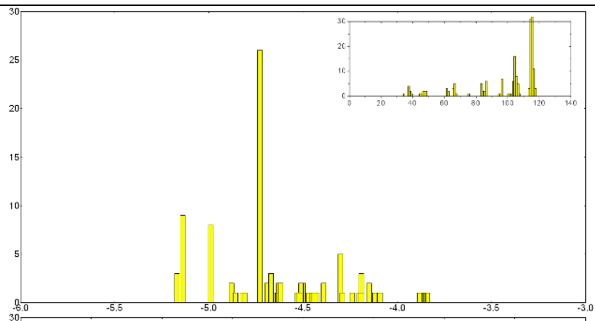

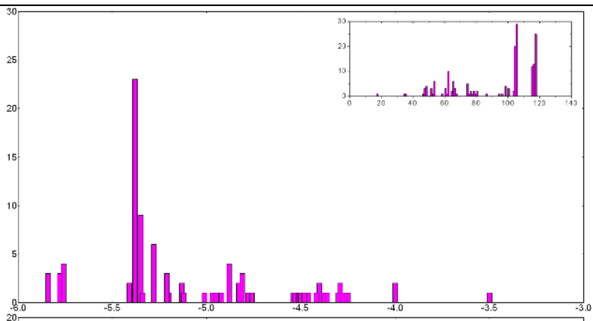

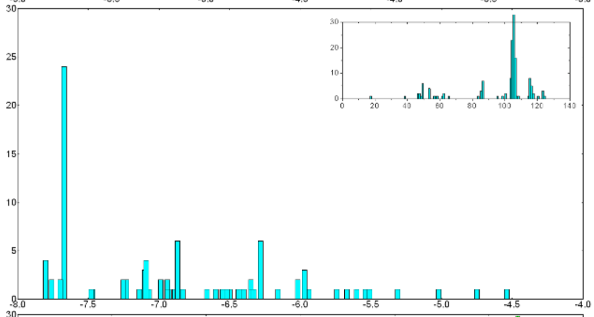

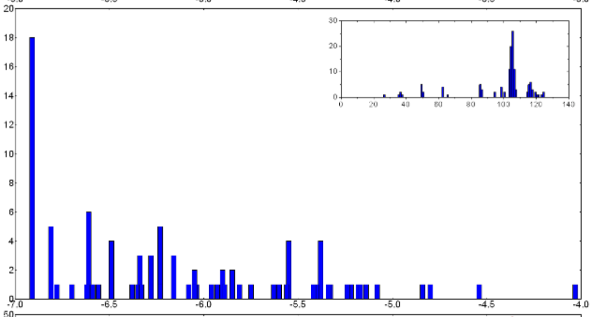

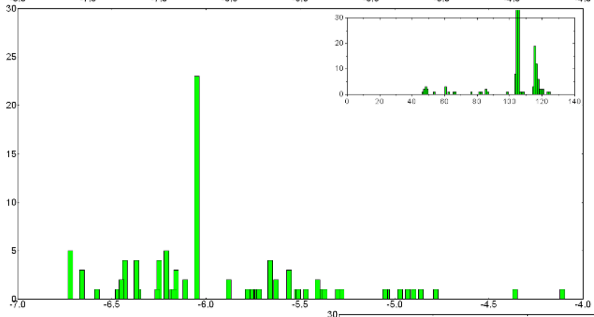

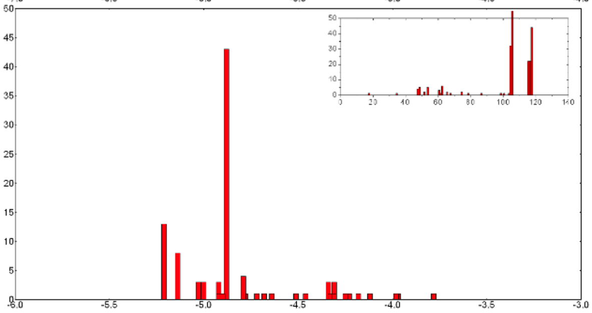

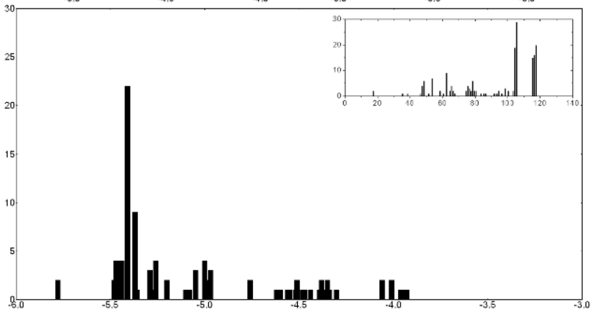

Cluster 6

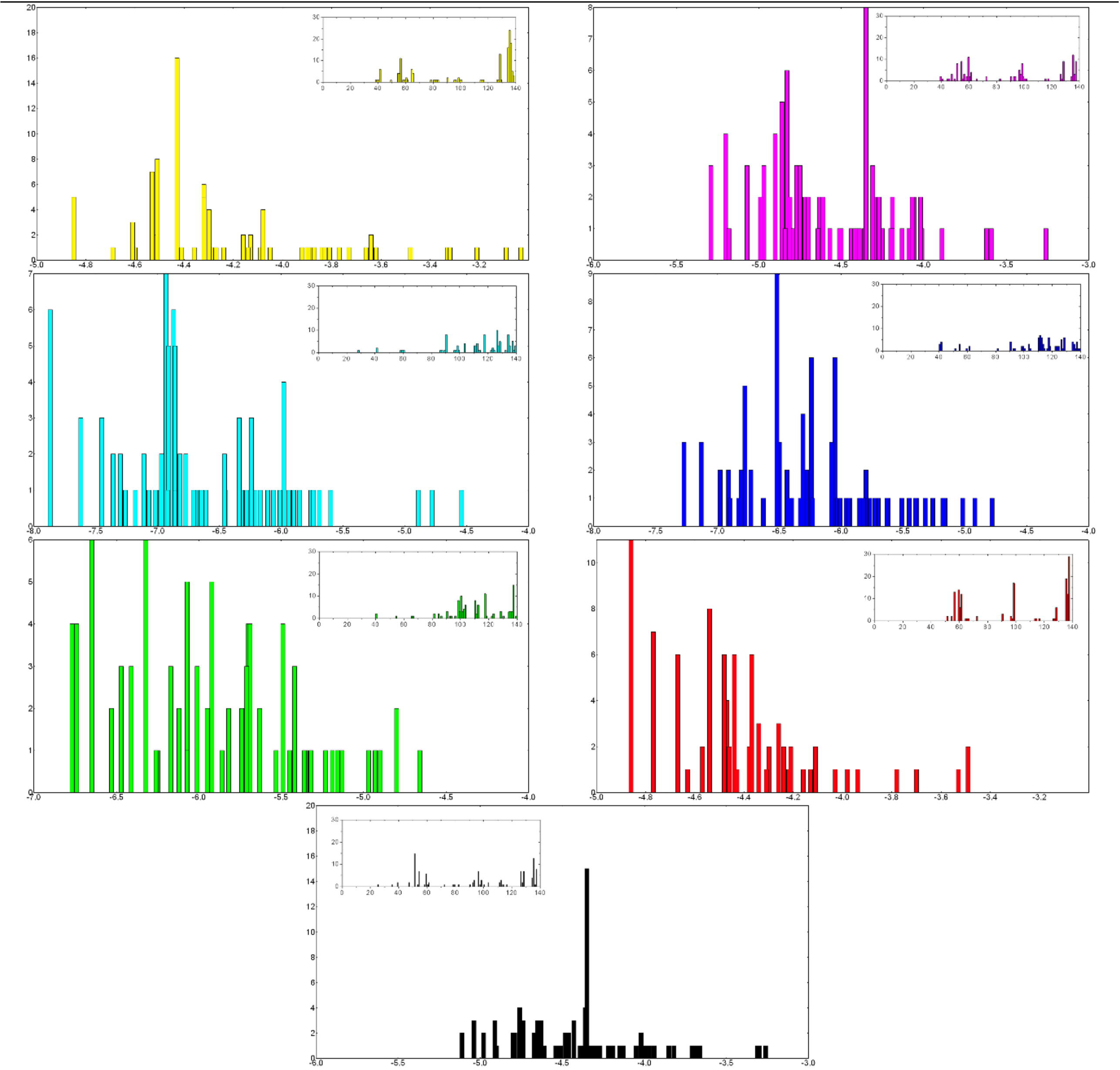

Figure S2. **Molecular docking**: Results for the 100 runs for each of the 6 AS representatives conformations with DCH (yellow), DHI (pink), DOP (light blue), DOP-H (blue), DQ (green), IQ (red) and LEUK (black) (54 complexes). Clustering of the doking results, as implemented in AUTODOCK, plotted as a function of the AUTODOCK scoring function (in Kcal/mol). Inset: The number of hits (defined in Tab. 2SI) between AS and the respective ligand.
