# Supplementary material for: Inhibition of α-Synuclein Fibrillization by Dopamine Is Mediated by Interactions with Five C-Terminal Residues and with E83 in the NAC Region
Source: PLoS One. 2008 Oct 14;3(10):e3394. doi: 10.1371/journal.pone.0003394 (PMC2566601; doi:10.1371/journal.pone.0003394)
Supplement: Figure S3 — MD simulations of the “stable” DOP/AS complexes. Structures and Cα contacts maps for the last MD snapshots. In the structures, residues 125–129 and E83 are colored in red and blue, respectively. In the contact maps, the x and y axis indicate the residues number in the AS sequence. The contact maps were calculated based on the Cα-Cα distance (a graph square is colored black at 0.0 A distance, to a linear gray scale between 0.0 and 10.0 A, and white when equal to or greater than 10.0 A). (0.81 MB PDF) [file pone.0003394.s003.pdf]

Cluster 1

DCH

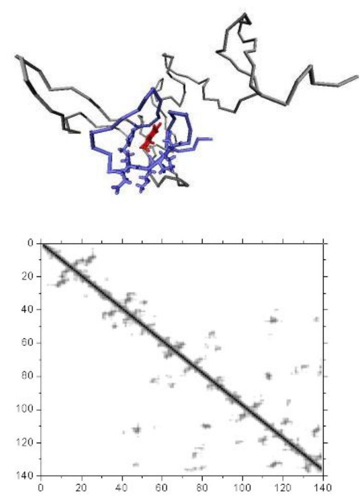

DHI

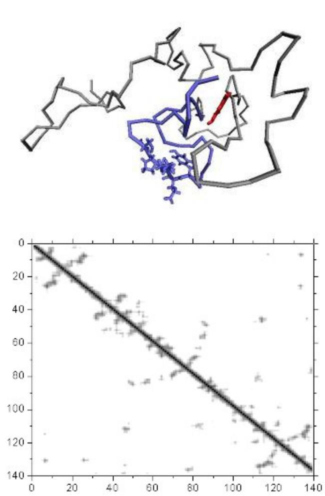

DOP

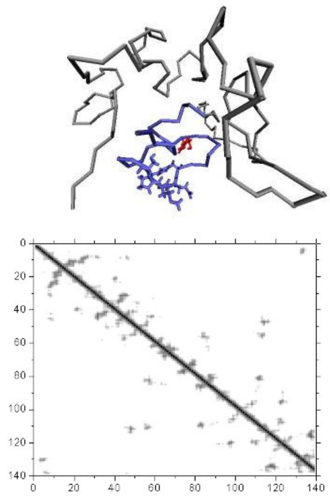

DOP-H

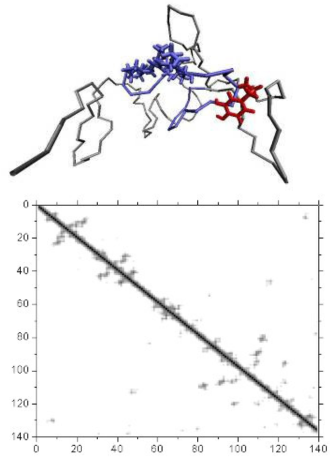

DQ

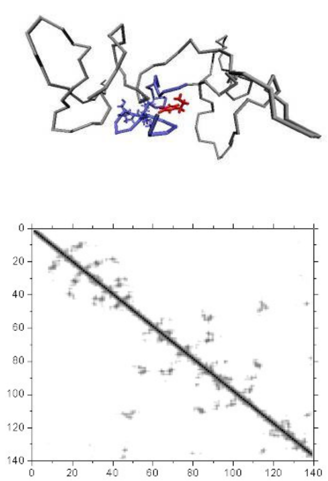

IQ

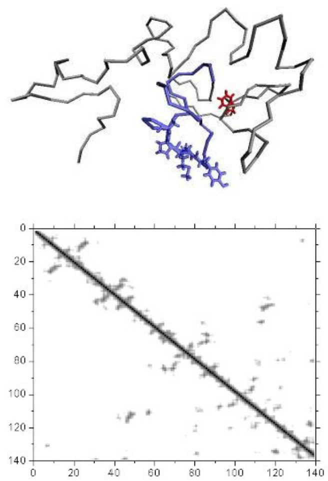

LEUK

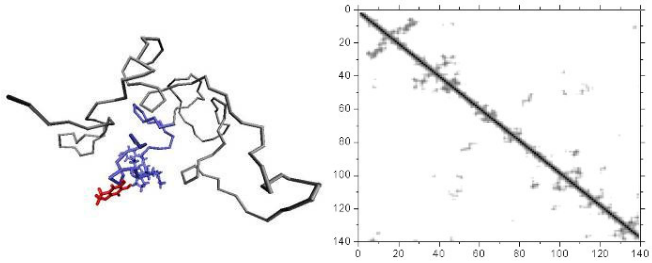

Cluster 2

DOP-H

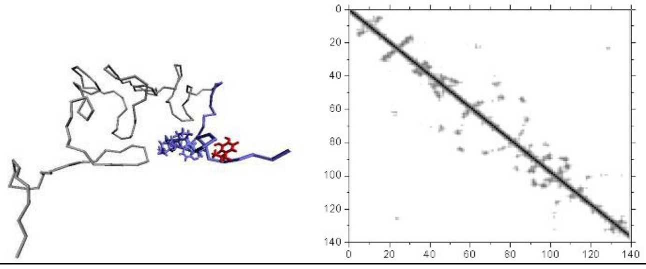

Cluster 3

DOP	DQ	IQ

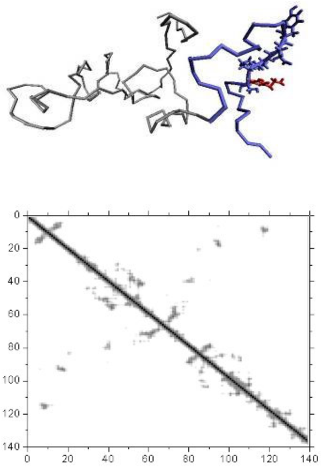

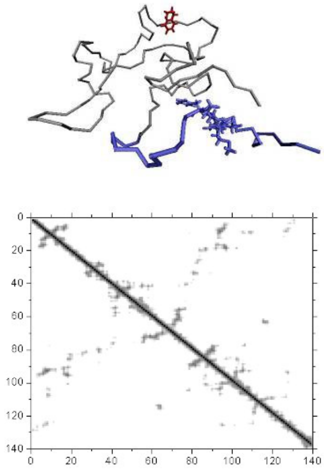

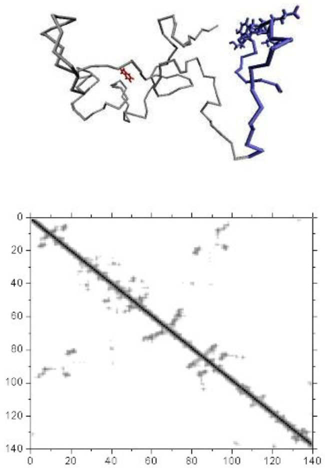

Cluster 4

DCH	DHI	DOP

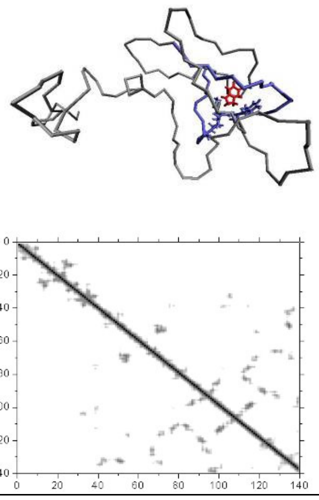

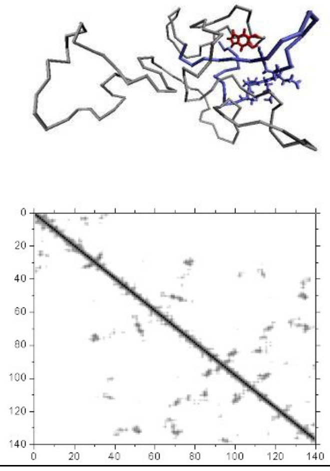

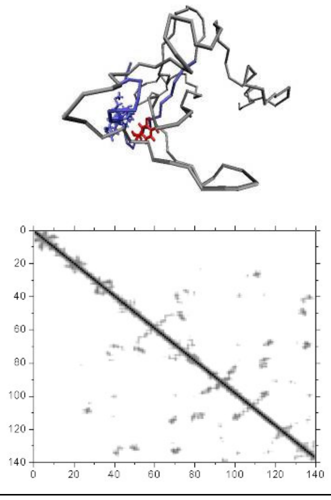

DOP-H

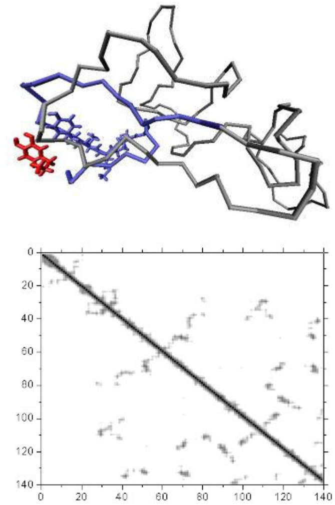

DQ

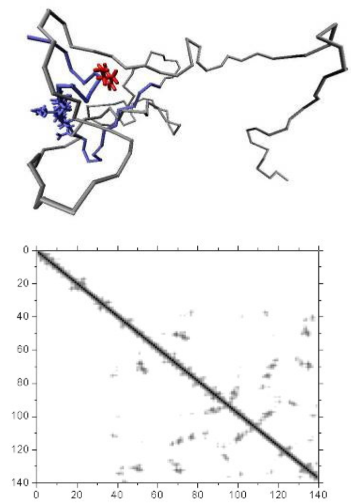

IQ

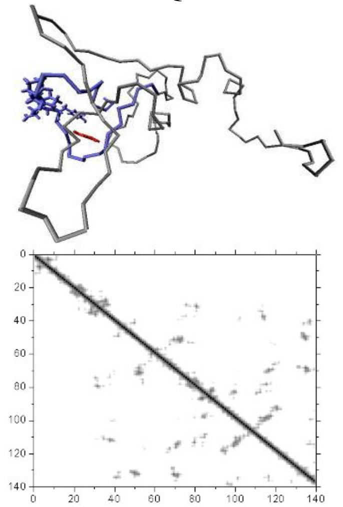

Cluster 5

DCH

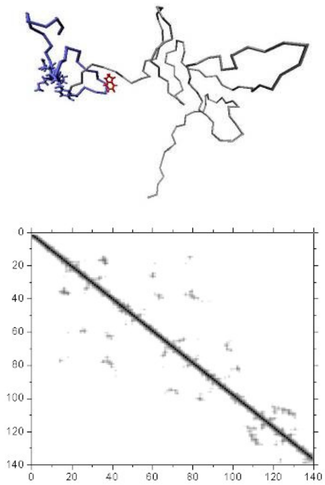

DOP-H

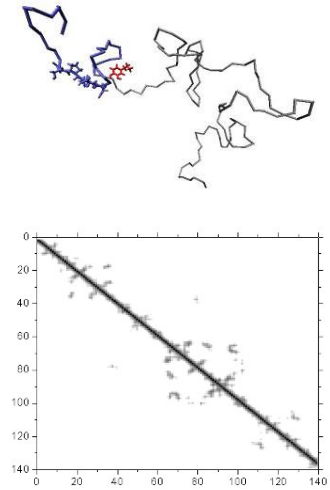

IQ

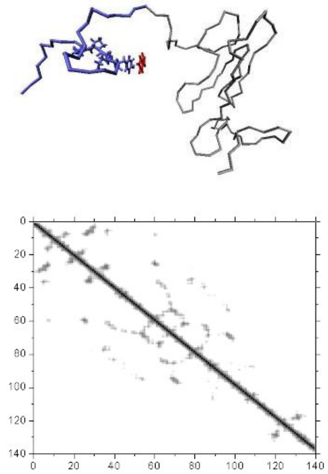

Cluster 6

DCH

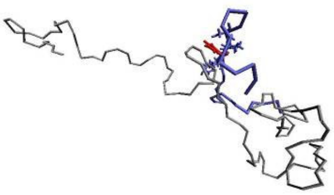

DHI

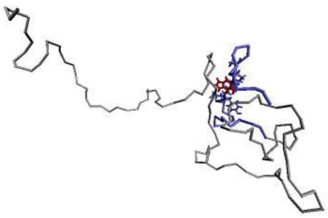

DOP

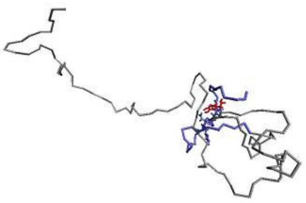

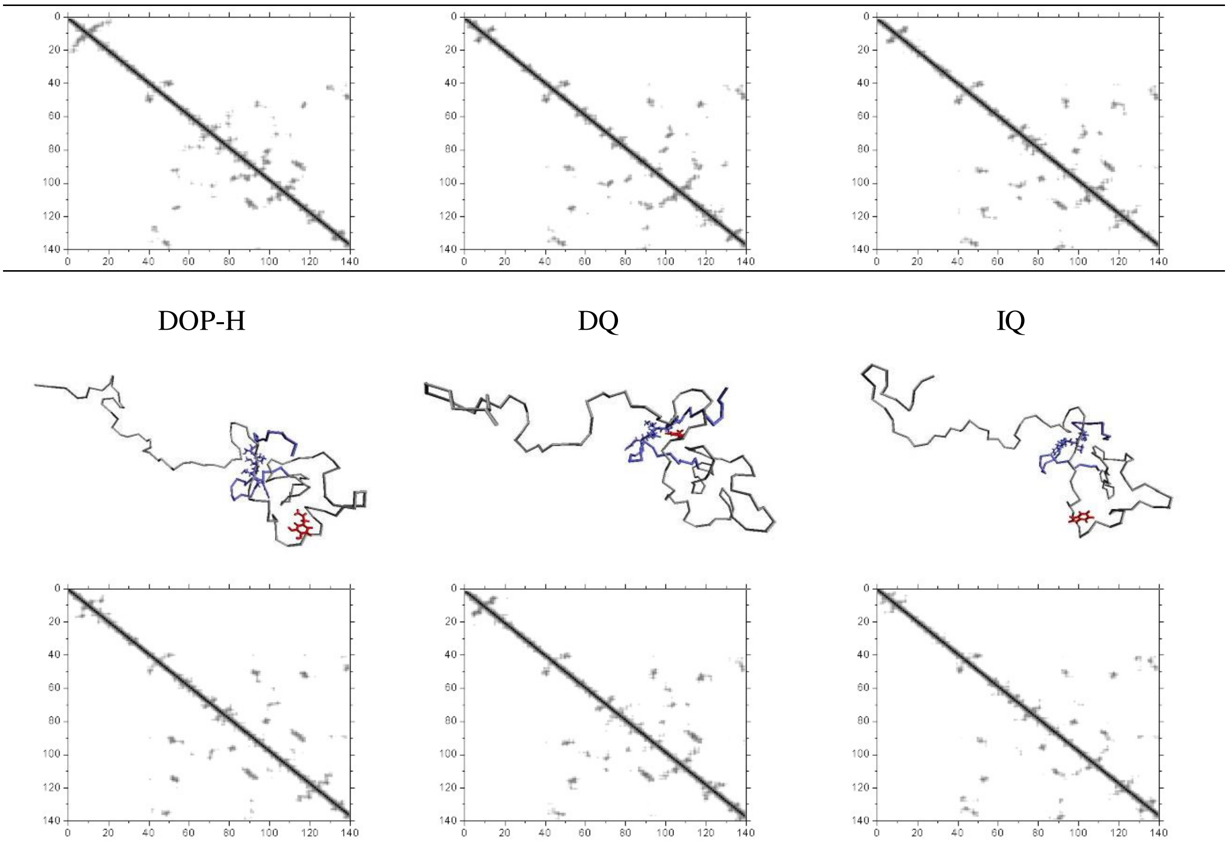

Figure S3. **MD simulations of the "stable" DOP/AS complexes.** Structures and Cα contacts maps for the last MD snapshots. In the structures, residues 125-129 and E83 are colored in red and blue, respectively. In the contact maps, the x and y axis indicate the residues number in the AS sequence. The contact maps were calculated based on the Cα-Cα distance (a graph square is colored black at 0.0 Å distance, to a linear gray scale between 0.0 and 10.0 Å, and white when equal to or greater than 10.0 Å).
