# Supplementary material for: Inhibition of α-Synuclein Fibrillization by Dopamine Is Mediated by Interactions with Five C-Terminal Residues and with E83 in the NAC Region
Source: PLoS One. 2008 Oct 14;3(10):e3394. doi: 10.1371/journal.pone.0003394 (PMC2566601; doi:10.1371/journal.pone.0003394)
Supplement: Figure S4 — MD simulations of the stable complexes. Ligand/protein interactions are represented using Ligplot program. (0.92 MB DOC) [file pone.0003394.s004.doc]

| Cluster 1 | | |
| --- | --- | --- |
| 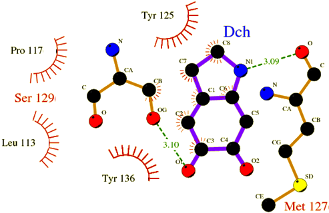 | 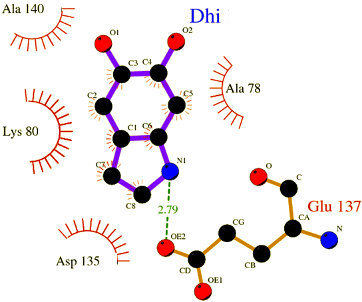 | 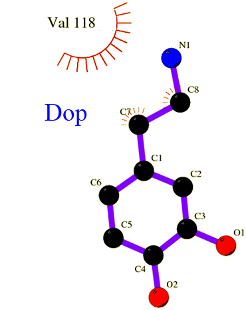 |
| 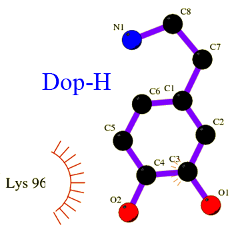 | 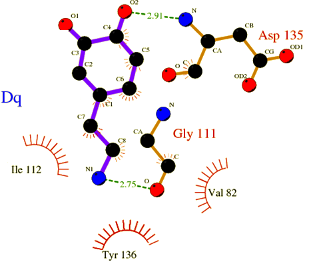 | 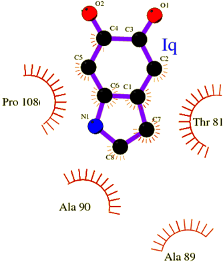 |
| 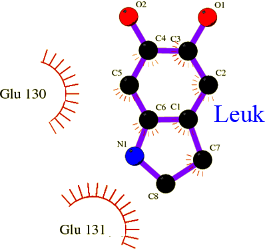 | | |
| Cluster2 | | |
| 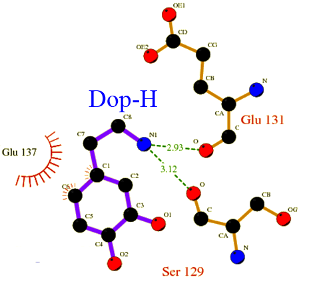 | | |
| Cluster 3 | | |
| 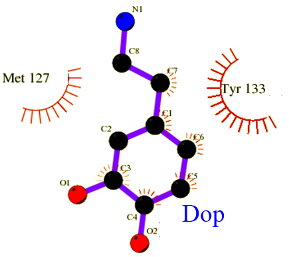 | 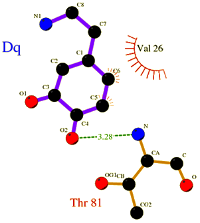 | 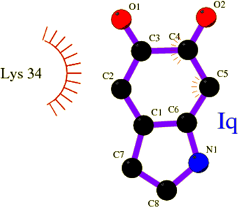 |
| Cluster 4 | | |
| 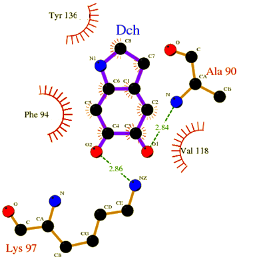 | 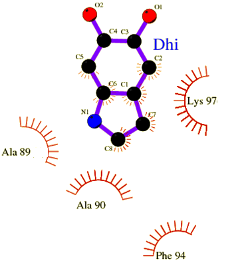 | 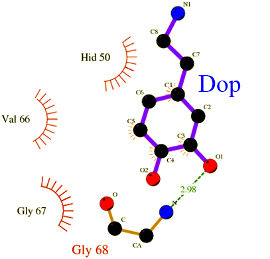 |
| 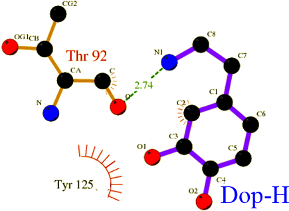 | 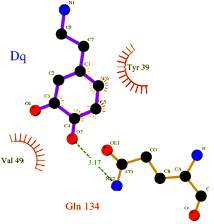 | 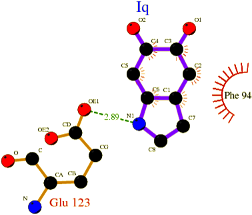 |
| Cluster 5 | | |
| 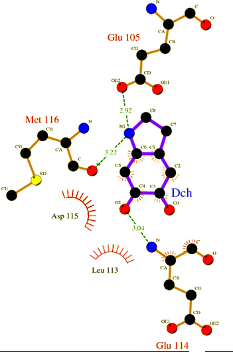 | 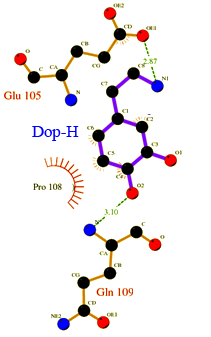 | 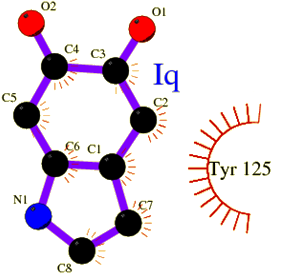 |
| Cluster 6 | | |
| 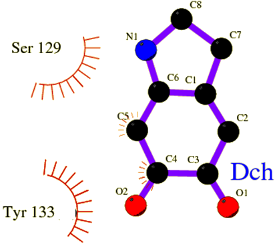 | 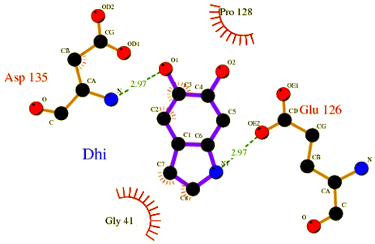 | 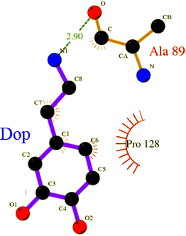 |
| 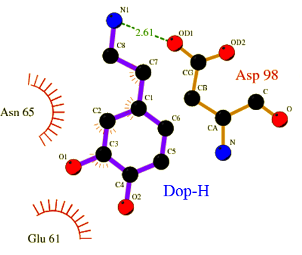 | 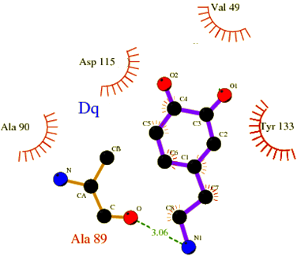 | 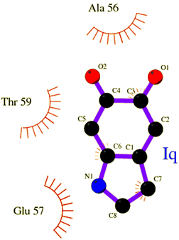 |
| 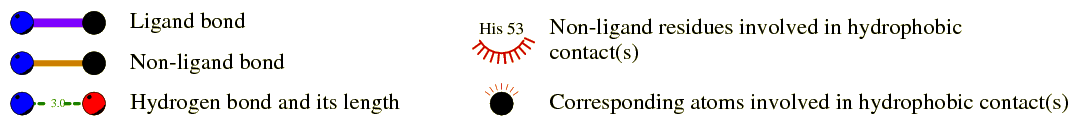 | | |

**Figure S4**. **MD simulations of the stable complexes.** Ligand/protein interactions are represented using Ligplot.
