# Supplementary material for: Inhibition of α-Synuclein Fibrillization by Dopamine Is Mediated by Interactions with Five C-Terminal Residues and with E83 in the NAC Region
Source: PLoS One. 2008 Oct 14;3(10):e3394. doi: 10.1371/journal.pone.0003394 (PMC2566601; doi:10.1371/journal.pone.0003394)
Supplement: Figure S5 — Structural fluctuations. Molecular dynamics of the NMR-derived conformations with the ligands. The Root mean square fluctuations (RMSF's, in A) are reported for the 26 stable complexes. (0.04 MB PDF) [file pone.0003394.s005.pdf]

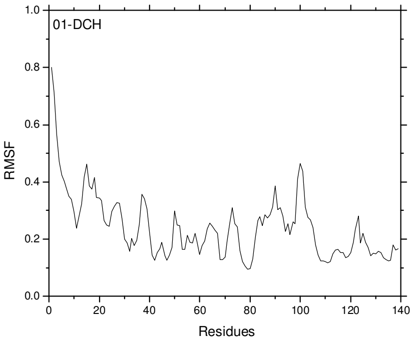
01-DCH
RMSF
Residues
0.0 0.2 0.4 0.6 0.8 1.0
0 20 40 60 80 100 120 140

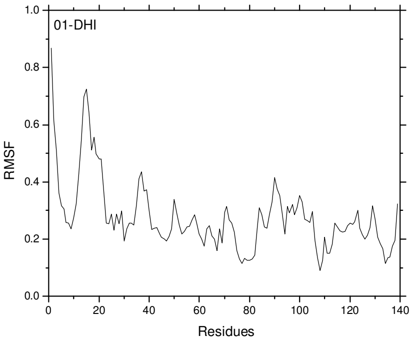
01-DHI
RMSF
Residues
0.0 0.2 0.4 0.6 0.8 1.0
0 20 40 60 80 100 120 140

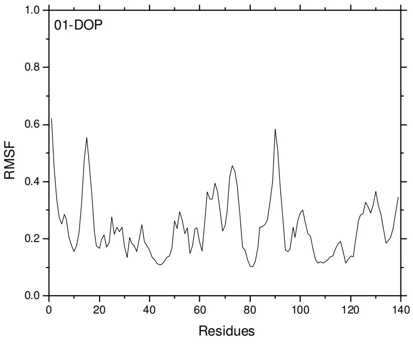
01-DOP
RMSF
Residues
0.0 0.2 0.4 0.6 0.8 1.0
0 20 40 60 80 100 120 140

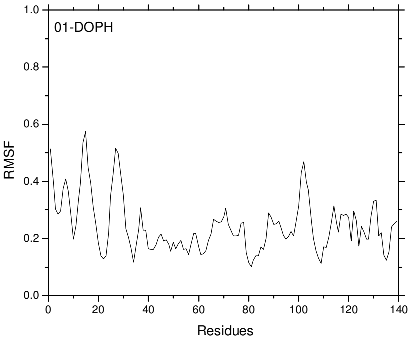
01-DOPH
RMSF
Residues
0.0 0.2 0.4 0.6 0.8 1.0
0 20 40 60 80 100 120 140

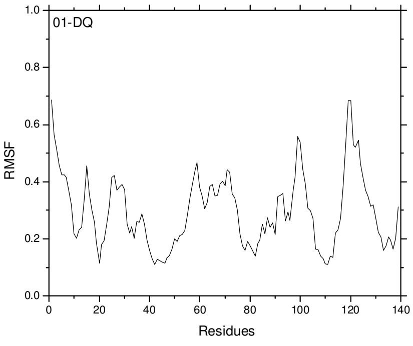
01-DQ
RMSF
Residues
0.0 0.2 0.4 0.6 0.8 1.0
0 20 40 60 80 100 120 140

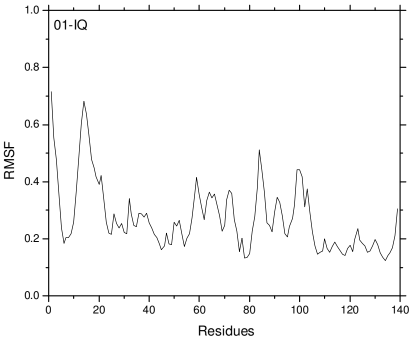
01-IQ
RMSF
Residues
0.0 0.2 0.4 0.6 0.8 1.0
0 20 40 60 80 100 120 140

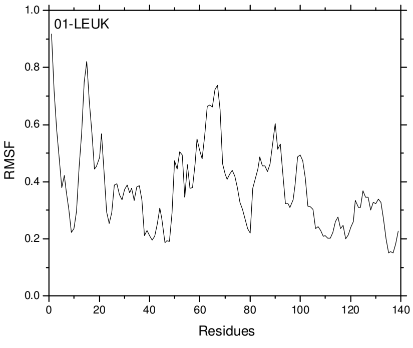
01-LEUK
RMSF
Residues
0.0 0.2 0.4 0.6 0.8 1.0
0 20 40 60 80 100 120 140

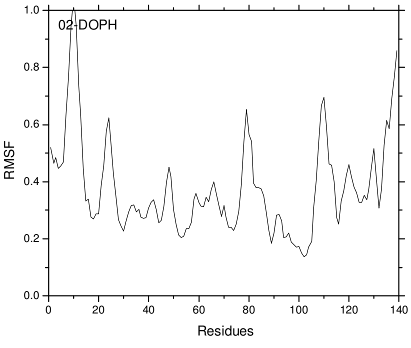
02-DOPH
RMSF
Residues
0.0 0.2 0.4 0.6 0.8 1.0
0 20 40 60 80 100 120 140

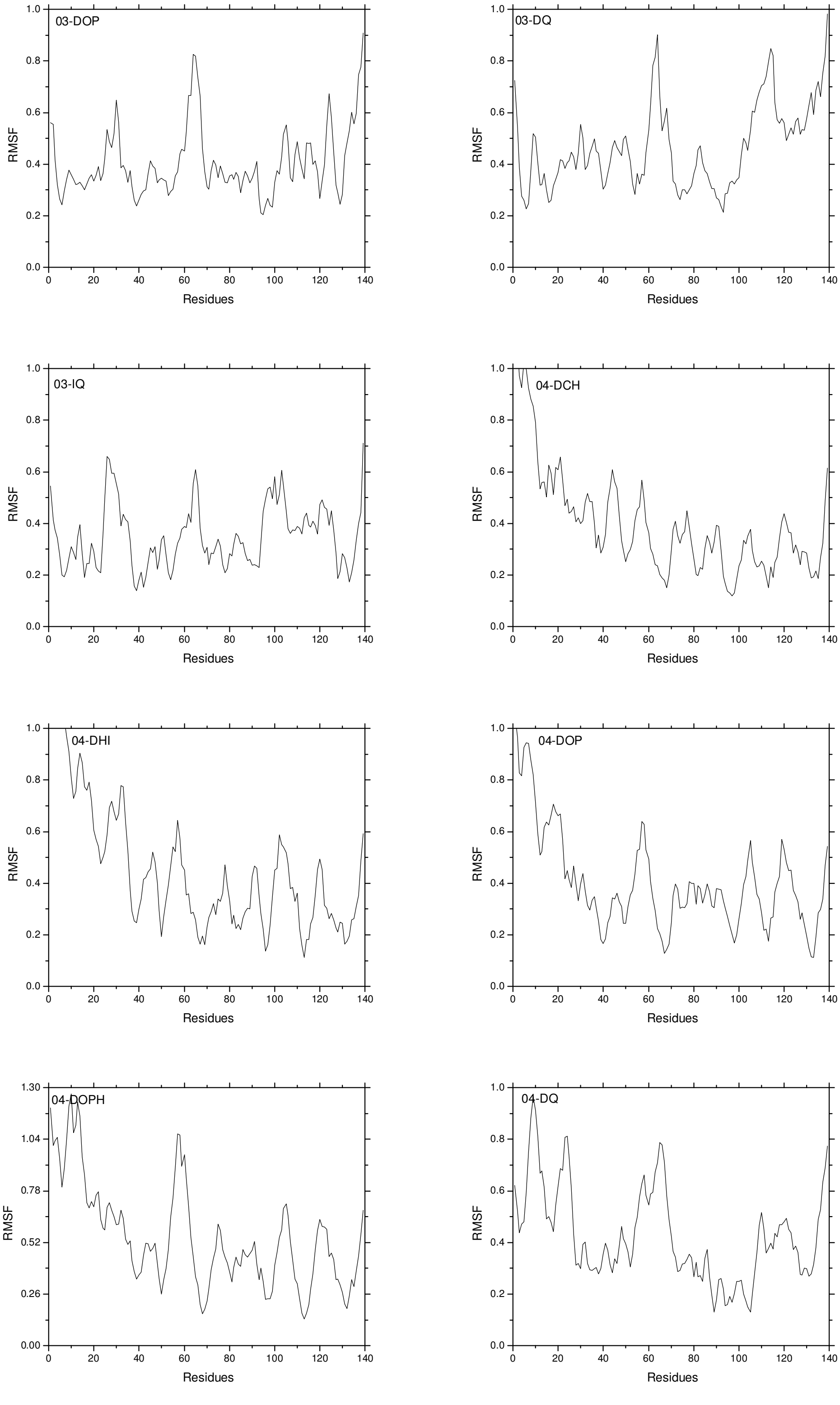
03-DOP
03-DQ
03-IQ
04-DCH
04-DHI
04-DOP
04-DOPH
04-DQ
RMSF
Residues

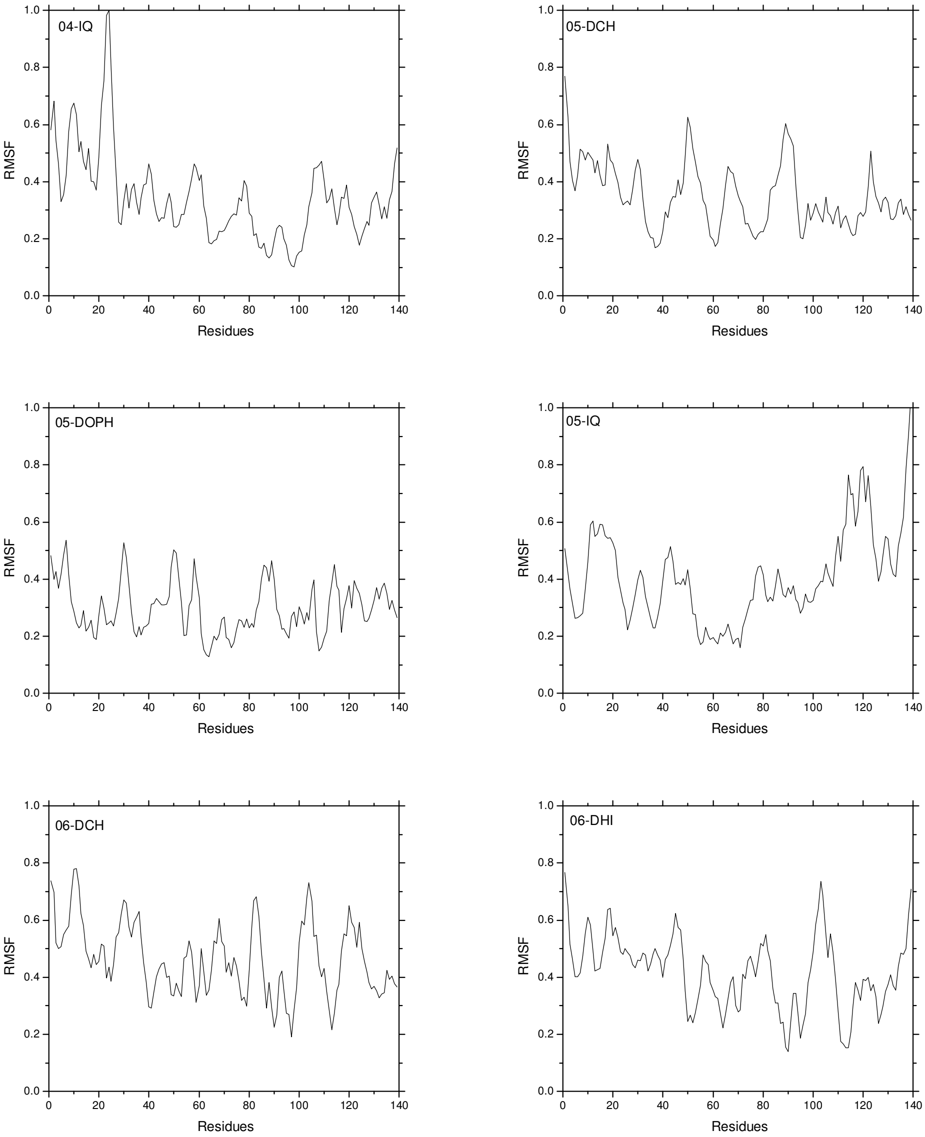
04-IQ
05-DCH
05-DOPH
05-IQ
06-DCH
06-DHI
RMSF
Residues
0.0
0.2
0.4
0.6
0.8
1.0
0
20
40
60
80
100
120
140

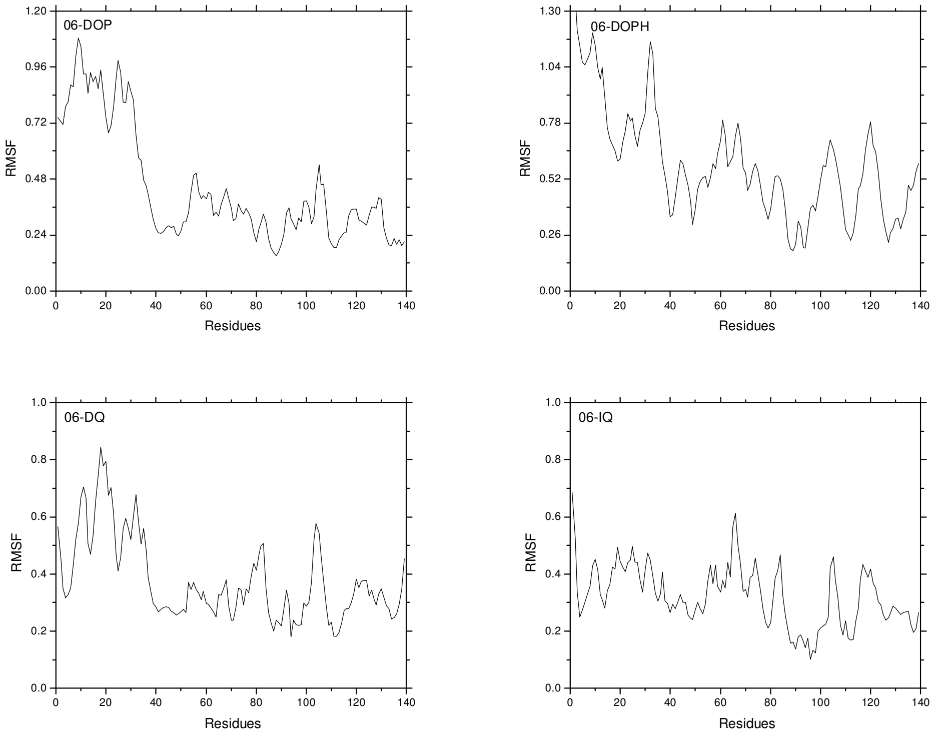

Figure S5. **Structural fluctuations.** Molecular dynamics of the NMR-derived conformations with the ligands. The Root mean square fluctuations (RMSF's, in Å) are reported for the 26 stable complexes.
