# Supplementary material for: Inhibition of α-Synuclein Fibrillization by Dopamine Is Mediated by Interactions with Five C-Terminal Residues and with E83 in the NAC Region
Source: PLoS One. 2008 Oct 14;3(10):e3394. doi: 10.1371/journal.pone.0003394 (PMC2566601; doi:10.1371/journal.pone.0003394)
Supplement: Figure S8 — MD simulations of the stable DOP-, DOP-H- and DCH-AS complexes. Final structures and contact maps for the last MD snapshots. Black to white scale as in Figure S3. Residues 125–129 and Glu 83 are colored in blue and the ligands are colored in red. (0.39 MB DOC) [file pone.0003394.s008.doc]

| Cluster 2 | | | | |
| --- | --- | --- | --- | --- |
| DOP-H  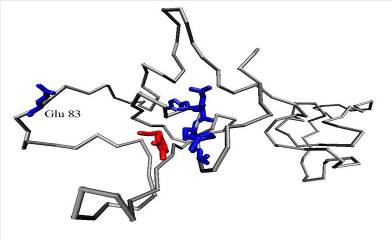  **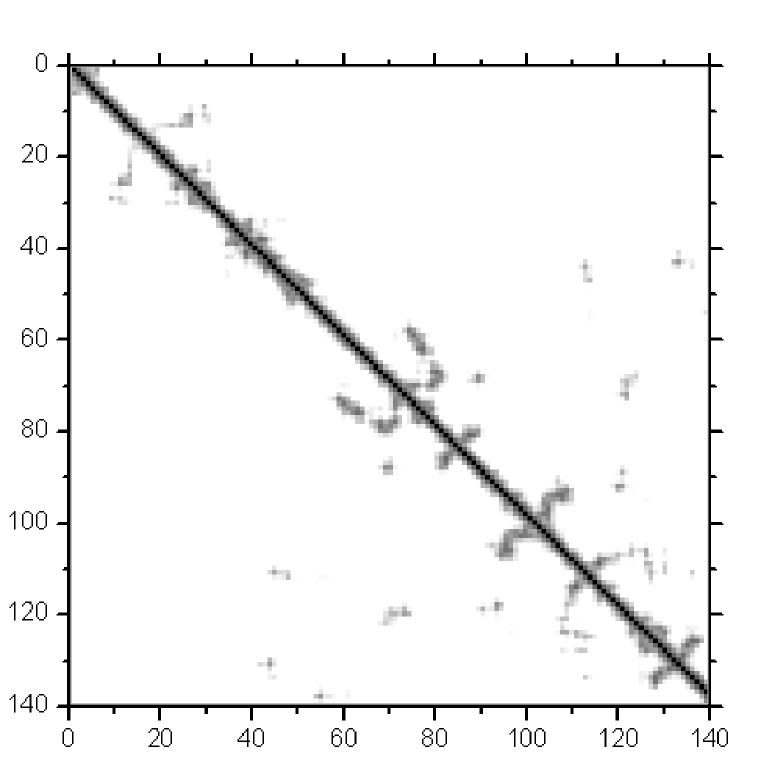** | | | DCH  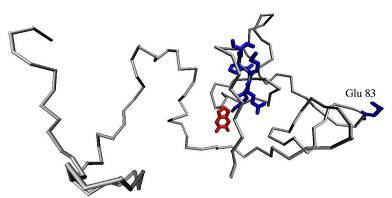  **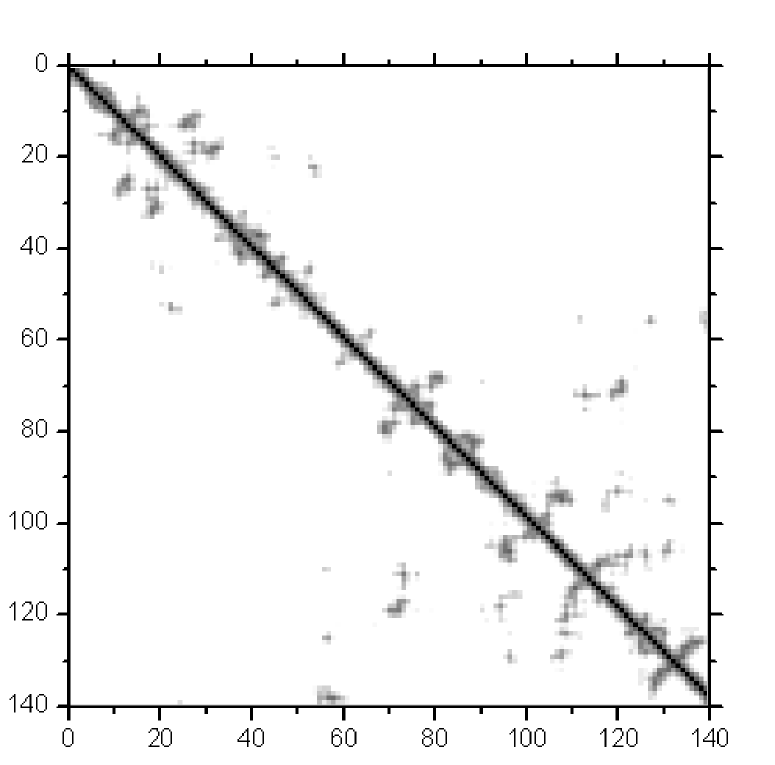** | |
| Cluster 3 | | | | |
| DOP  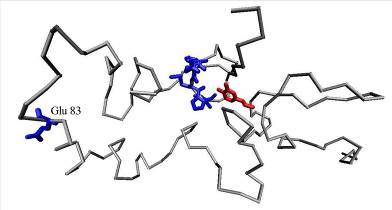  **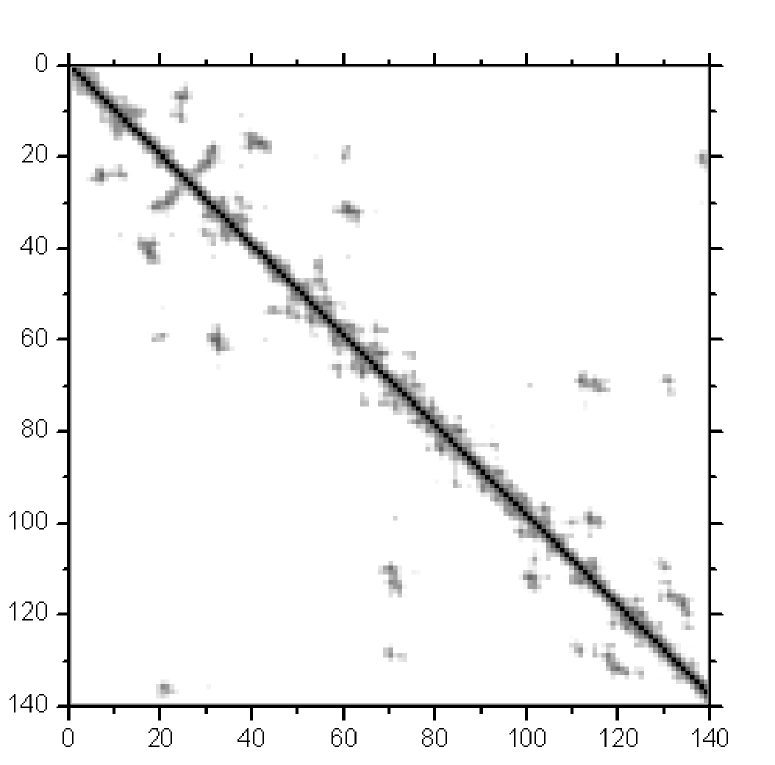** | | DCH  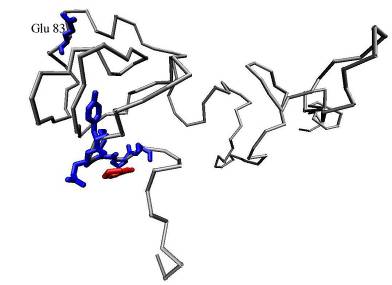  **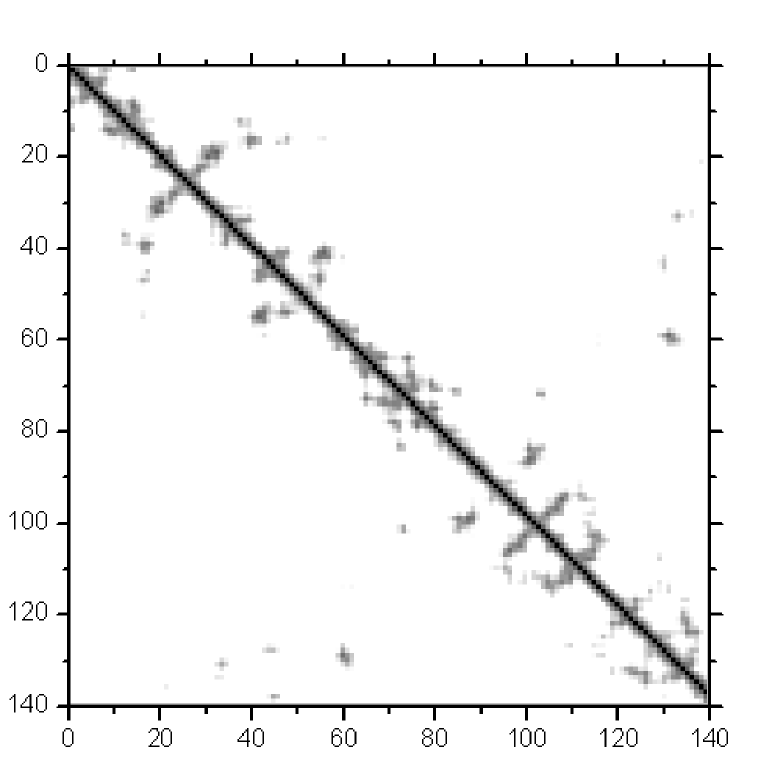** | | |
| Cluster 4 | | | | |
| DOP  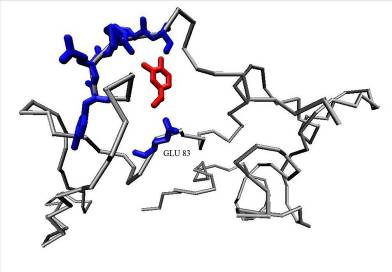  **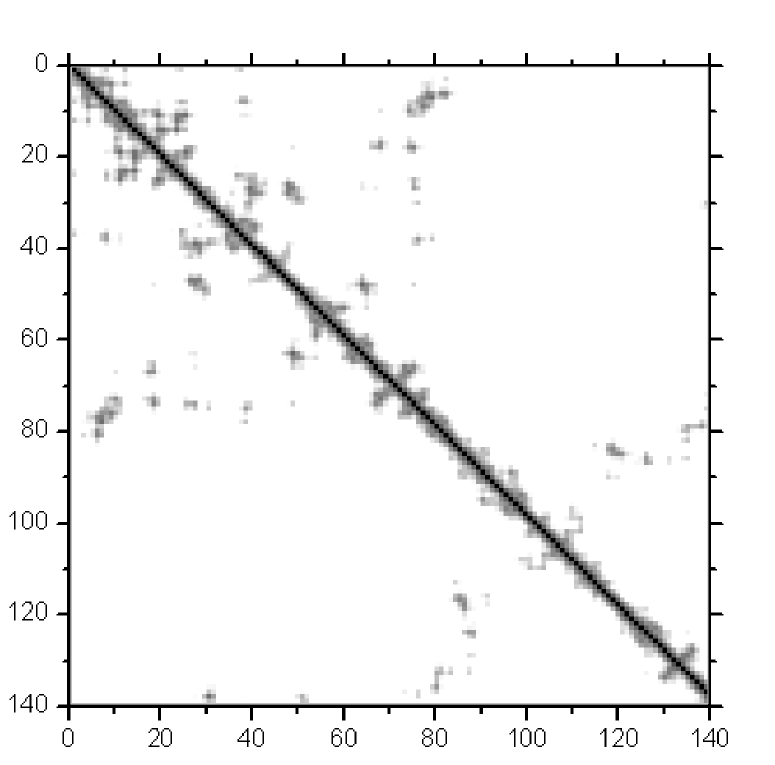** | DOP-H  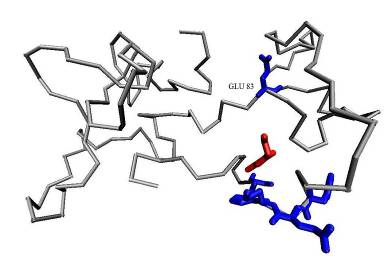  **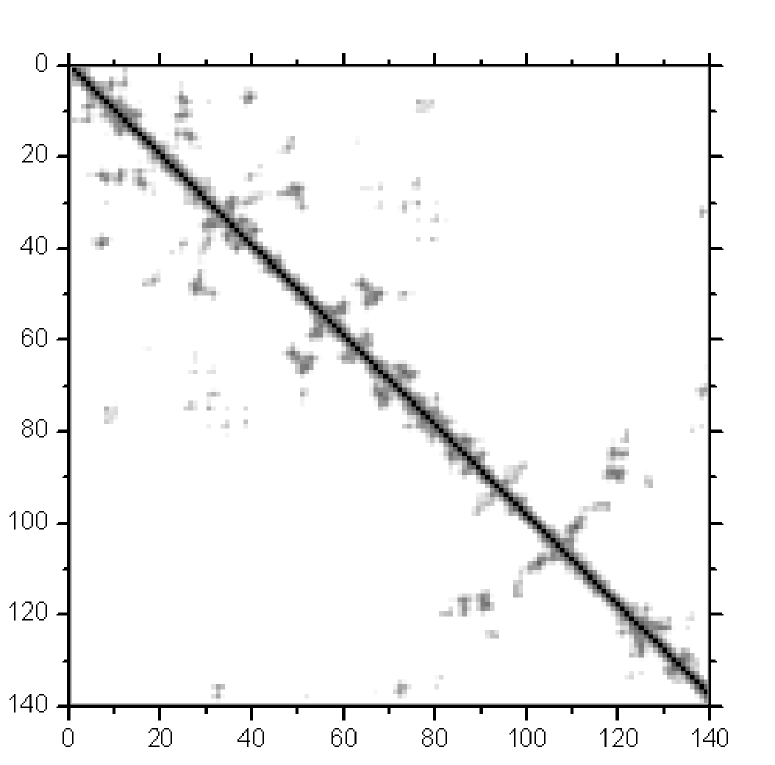** | | | DCH  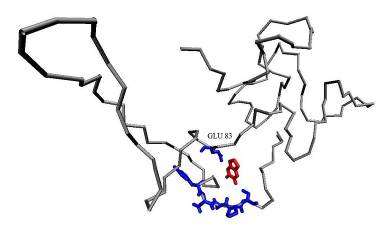  **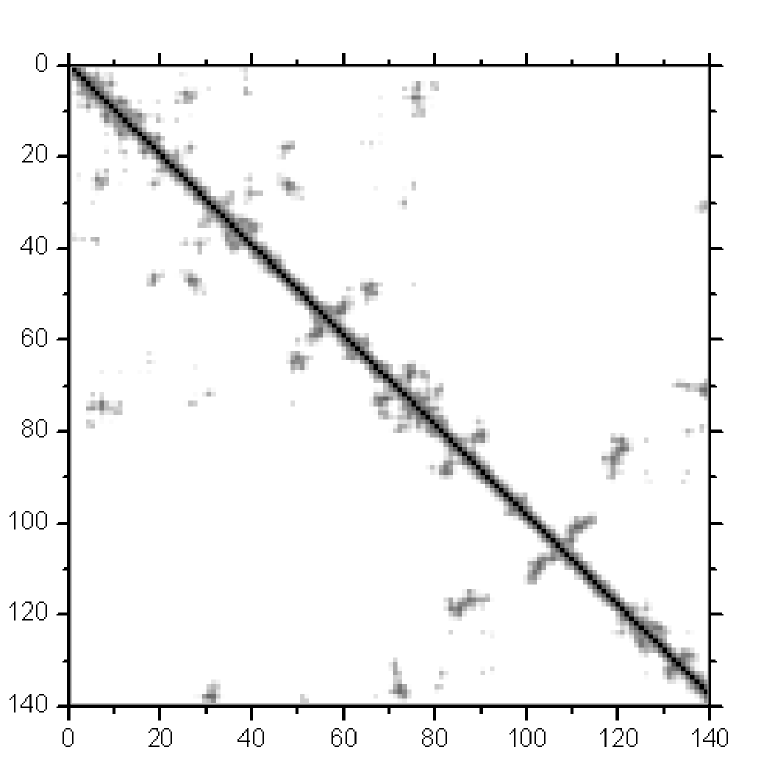** |
| Cluster 5 | | | | |
| DOP  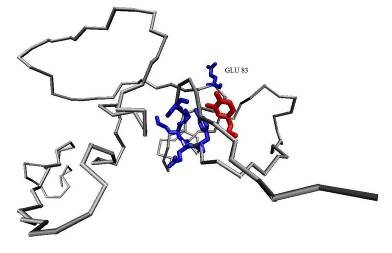  **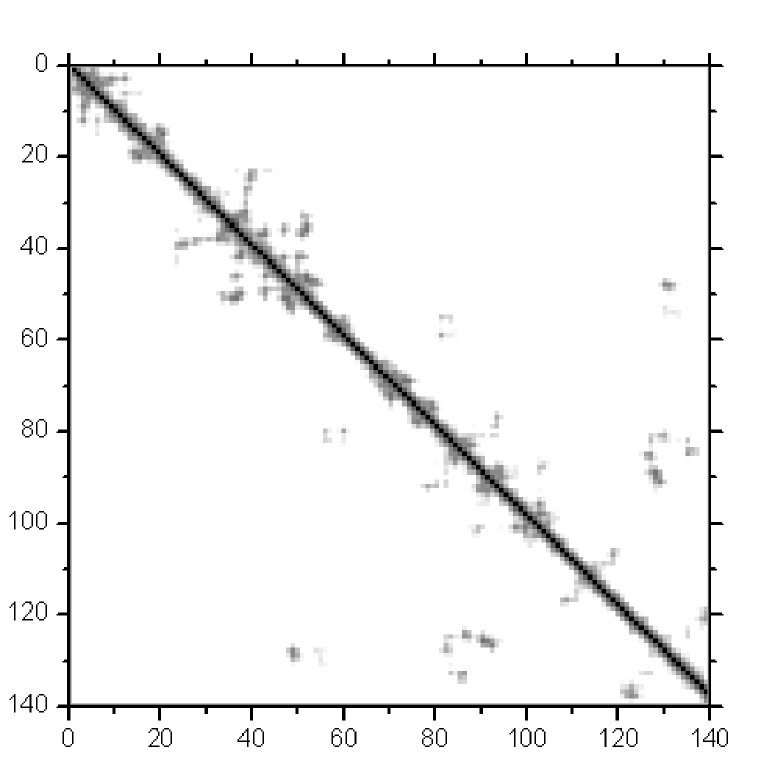** | | | DCH  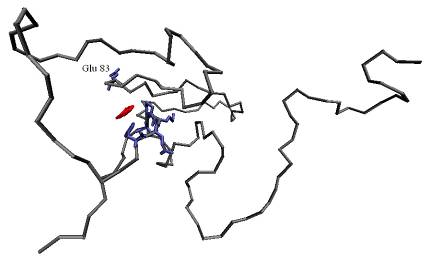  **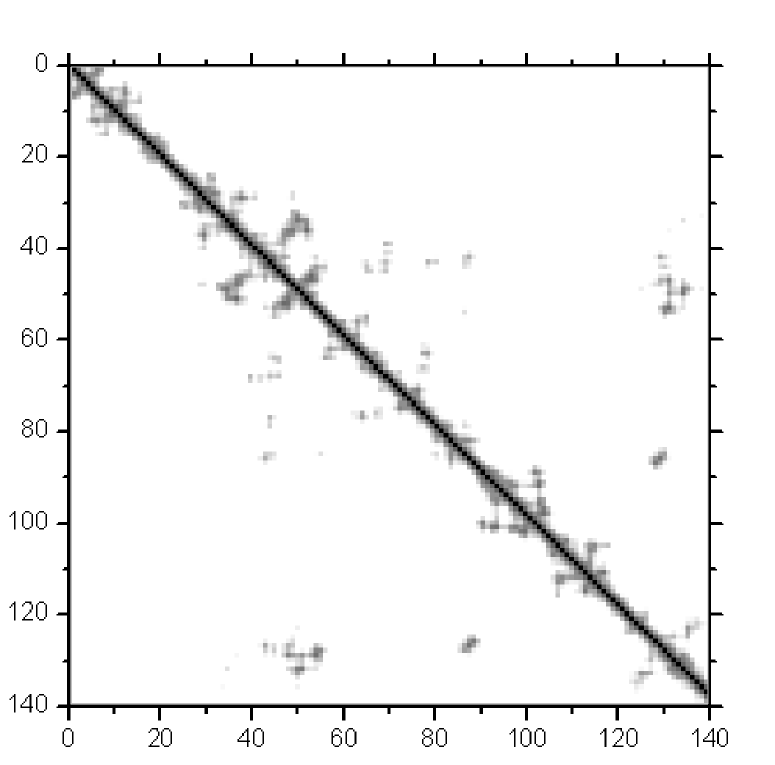** | |
| Cluster 6 | | | | |
| DOP-H  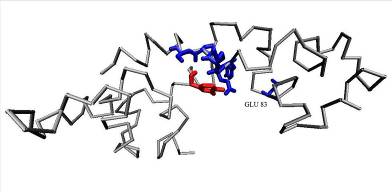  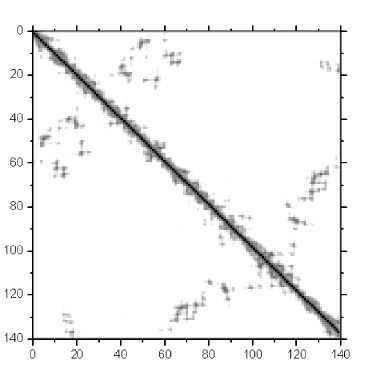 | | | DCH  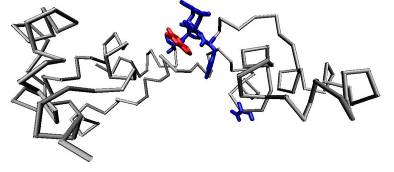  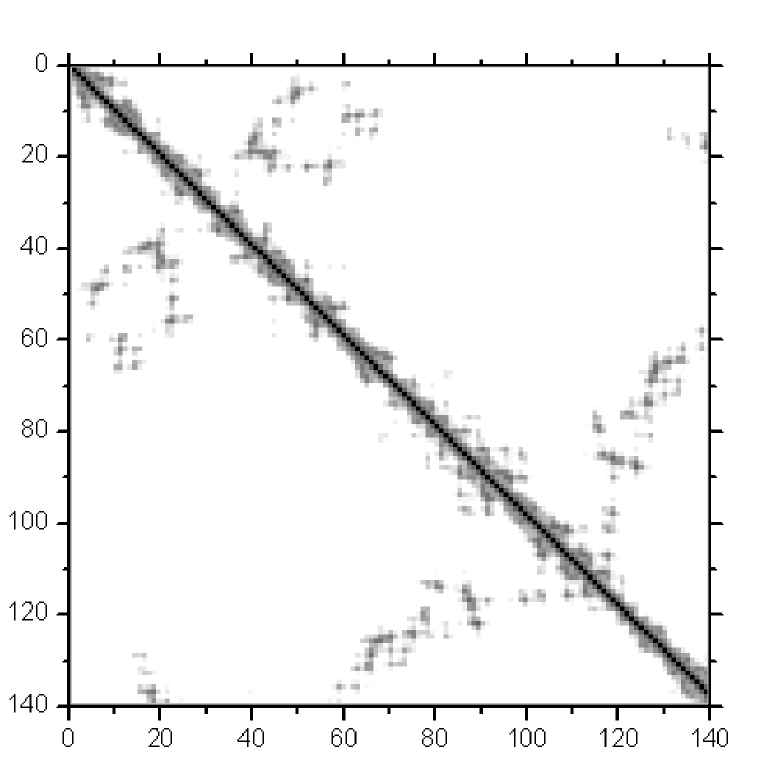 | |

**Figure S8**. **MD simulations of the stable DOP-, DOP-H- and DCH-AS complexes.** Final structures and contact maps for the last MD snapshots. Black to white scale as in Figure S3.

Residues 125-129 and Glu 83 are colored in blue and the ligands are colored in red.
