# Supplementary material for: Inhibition of α-Synuclein Fibrillization by Dopamine Is Mediated by Interactions with Five C-Terminal Residues and with E83 in the NAC Region
Source: PLoS One. 2008 Oct 14;3(10):e3394. doi: 10.1371/journal.pone.0003394 (PMC2566601; doi:10.1371/journal.pone.0003394)
Supplement: Figure S9 — MD simulations in the “stable” complexes from the second cluster analysis. The ligands and the residues involved in the interactions are shown in sticks. Hydrogen bonding and hydrophobic interactions are shown as dashed lines. Snapshots taken from the last frame of the MD simulations. (0.26 MB PDF) [file pone.0003394.s009.pdf]

Cluster 2

DOP-H

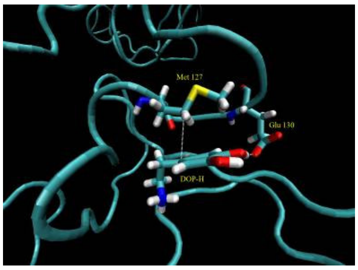

DCH

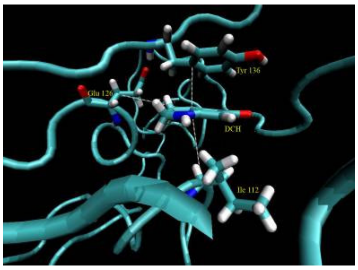

Cluster 3

DOP

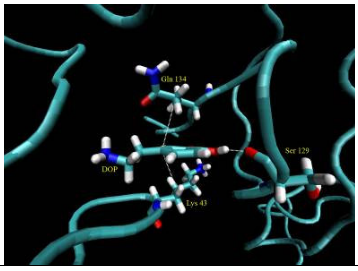

DCH

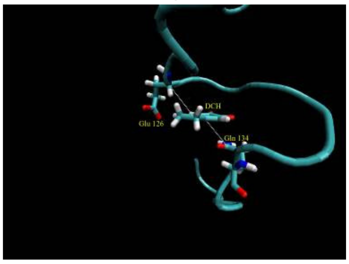

Cluster 4

DOP

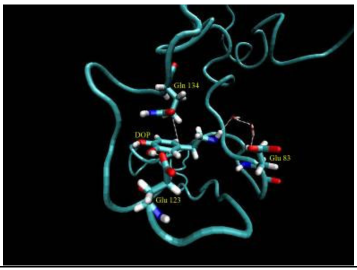

DOP-H

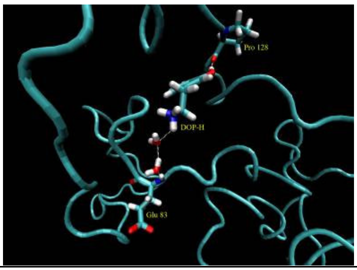

DCH

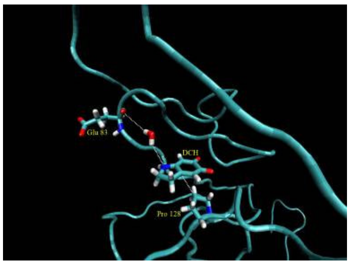

Cluster 5

DOP

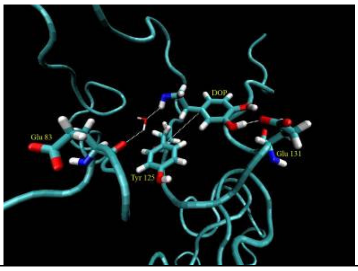

DCH

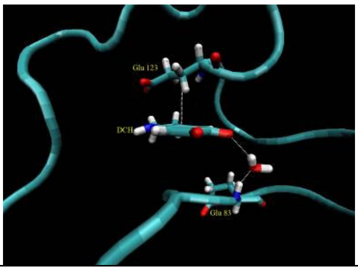

Cluster 6

DOP-H

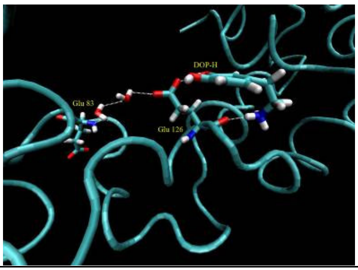

DCH

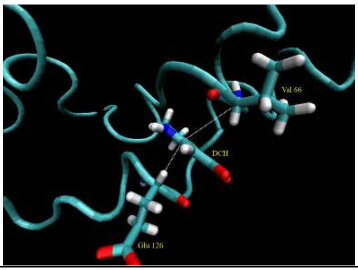

Figure S9. **MD simulations in the "stable" complexes from the second cluster analysis.** The ligands and the residues involved in the interactions are shown in sticks. Hydrogen bonding and hydrophobic interactions are shown as dashed lines. Snapshots taken from the last frame of the MD simulations.
