# Supplementary material for: Inhibition of α-Synuclein Fibrillization by Dopamine Is Mediated by Interactions with Five C-Terminal Residues and with E83 in the NAC Region
Source: PLoS One. 2008 Oct 14;3(10):e3394. doi: 10.1371/journal.pone.0003394 (PMC2566601; doi:10.1371/journal.pone.0003394)
Supplement: Table S1 — MD simulations. Atoms labeling and RESP atomic charges of the ligands in Figure 1. (0.12 MB DOC) [file pone.0003394.s012.doc]

**Table S1**. **MD simulations**. Atoms labeling and RESP atomic charges of the ligands in Fig. 1.

|  | DCH | DHI | DOP | DOP-H | DQ | IQ | LEUK |
| --- | --- | --- | --- | --- | --- | --- | --- |
| C1 | 0.185 | 0.070 | -0.072 | -0.033 | -0.029 | 0.228 | 0.093 |
| H1 | 0.186 | 0.179 | 0.469 | 0.477 | 0.158 | 0.194 | 0.177 |
| C2 | -0.446 | -0.328 | -0.327 | -0.293 | -0.281 | -0.397 | -0.378 |
| H2 | 0.212 | 0.186 | 0.461 | 0.471 | 0.189 | 0.202 | 0.220 |
| C3 | 0.627 | 0.226 | 0.329 | 0.300 | 0.512 | 0.534 | 0.197 |
| O1 | -0.498 | -0.566 | -0.626 | -0.594 | -0.485 | -0.482 | -0.663 |
| H4 | 0.028 | 0.208 | 0.178 | 0.196 | 0.071 | 0.191 | 0.018 |
| C4 | 0.494 | 0.284 | 0.268 | 0.281 | 0.588 | 0.550 | 0.314 |
| O2 | -0.515 | -0.557 | -0.658 | -0.625 | -0.493 | -0.501 | -0.610 |
| H5 | 0.034 | 0.318 | 0.162 | 0.179 | 0.071 | 0.322 | 0.027 |
| C5 | -0.496 | -0.385 | -0.331 | -0.301 | -0.291 | -0.449 | -0.353 |
| H3 | 0.028 | 0.162 | 0.217 | 0.205 | 0.169 | 0.197 | 0.018 |
| C6 | 0.040 | 0.037 | -0.175 | -0.207 | -0.180 | 0.083 | -0.020 |
| C7 | 0.054 | -0.221 | -0.031 | -0.064 | -0.046 | -0.350 | 0.031 |
| H6 | 0.034 | 0.426 | 0.059 | 0.073 | -0.008 |  | 0.027 |
| H7 | 0.302 | 0.424 | 0.059 | 0.073 | -0.008 |  | 0.294 |
| C8 | 0.058 | -0.252 | 0.296 | 0.034 | 0.342 | -0.053 | 0.050 |
| H8 |  |  | 0.004 | 0.098 | 0.395 |  | 0.470 |
| H9 |  |  | 0.004 | 0.098 | 0.395 |  | 0.467 |
| N1 | -0.325 | -0.209 | -1.047 | -0.278 | -1.070 | -0.269 | -0.378 |
| H10 |  |  | 0.381 | 0.303 |  |  |  |
| H11 |  |  | 0.381 | 0.303 |  |  |  |
| H12 |  |  |  | 0.303 |  |  |  |
| Net Charge | 0.0 | 0.0 | 0.0 | 1.0 | 0.0 | 0.0 | 0.0 |
