# Supplementary material for: Inhibition of α-Synuclein Fibrillization by Dopamine Is Mediated by Interactions with Five C-Terminal Residues and with E83 in the NAC Region
Source: PLoS One. 2008 Oct 14;3(10):e3394. doi: 10.1371/journal.pone.0003394 (PMC2566601; doi:10.1371/journal.pone.0003394)
Supplement: Table S2 — Molecular Docking: Top) Number of hits between α-synuclein (AS) and the seven ligands as obtained by 4,200 docking runs of Autodock. The hits are defined here when the distance between at least one AS's Cα atom and the ligands' center of mass is lower than 5 A. Bottom) Relative contribution for the binding of the C-terminal regions, calculated as percentages of the total number of ligand-protein contacts (0.11 MB DOC) [file pone.0003394.s013.doc]

**Table S2. Molecular Docking:** *Top*) Number of hits between α-synuclein (AS) and the seven ligands as obtained by 4,200 docking runs of Autodock. The hits are defined here when the distance between at least one AS’s Cα atom and the ligands’ center of mass is lower than 5 Å. *Bottom)* Relative contribution for the binding of the C-terminal regions, calculated as percentages of the total number of ligand-protein contacts.

| 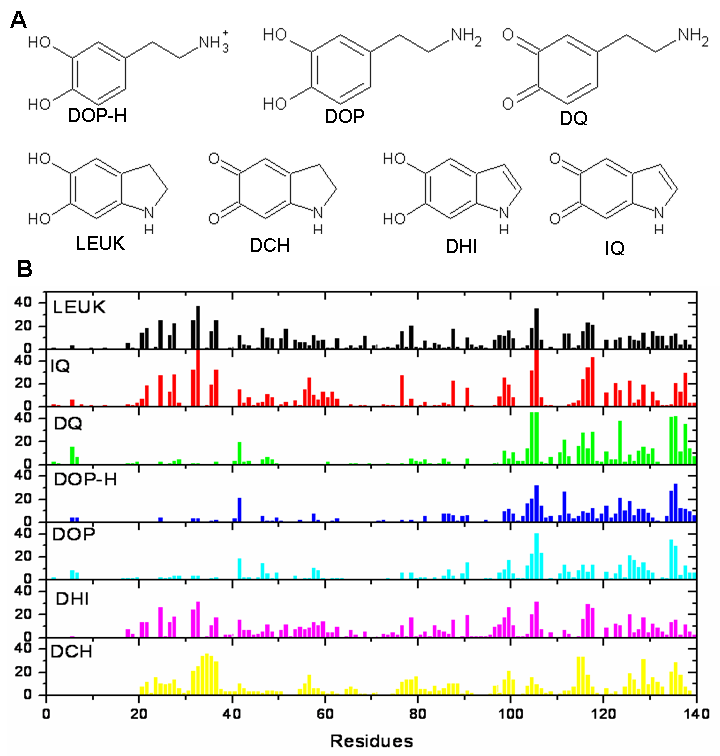 | | | | | | | |
| --- | --- | --- | --- | --- | --- | --- | --- |
| Region | DCH | DHI | DOP | DOP-H | DQ | IQ | LEUK |
| 100-140 | 40.7 % | 39.2 % | 62.2 % | 71.3 % | 74.7 % | 42.7 % | 38.3 % |
| 110-140 | 32.3 % | 27.3 % | 43.4 % | 51.9 % | 55.9 % | 30.0 % | 27.7 % |
| 120-140 | 21.1 % | 18.3 % | 32.3 % | 37.0 % | 38.2 % | 19.1 % | 17.6 % |
| 125-129 | 7.6 % | 5.4 % | 10.4 % | 9.5 % | 5.9 % | 5.1 % | 4.6 % |
