# Supplementary material for: Inhibition of α-Synuclein Fibrillization by Dopamine Is Mediated by Interactions with Five C-Terminal Residues and with E83 in the NAC Region
Source: PLoS One. 2008 Oct 14;3(10):e3394. doi: 10.1371/journal.pone.0003394 (PMC2566601; doi:10.1371/journal.pone.0003394)
Supplement: Table S3 — Stabilities. The stabilities of the local interactions between the ligand and AS for all MD simulations are reported here. (0.03 MB DOC) [file pone.0003394.s014.doc]

**Table S3.** **Stabilities**. The stabilities of the local interactions between the ligand and AS for all MD simulations are reported here.

|  | DCH | DHI | DOP | DOP-H | DQ | IQ | LEUK |
| --- | --- | --- | --- | --- | --- | --- | --- |
| Repr. 1 | Stable | Stable | Stable | Stable | Stable | Stable | Stable |
| Repr. 2 | Unstable | Unstable | Unstable | Stable | Unstable | Unstable | Unstable |
| Repr. 3 | Unstable | Unstable | Stable | Unstable | Stable | Stable | Unstable |
| Repr. 4 | Stable | Stable | Stable | Stable | Stable | Stable | Unstable |
| Repr. 5 | Stable | Unstable | Unstable | Stable | Unstable | Stable | Unstable |
| Repr. 6 | Stable | Stable | Stable | Stable | Stable | Stable | Unstable |
| MD-Derived | Stable | Stable | Stable | Stable | Stable | Unstable | Unstable |
