# Supplementary material for: Inhibition of α-Synuclein Fibrillization by Dopamine Is Mediated by Interactions with Five C-Terminal Residues and with E83 in the NAC Region
Source: PLoS One. 2008 Oct 14;3(10):e3394. doi: 10.1371/journal.pone.0003394 (PMC2566601; doi:10.1371/journal.pone.0003394)
Supplement: Table S4 — MD simulations. RMSD (A) and radius of gyration (A) of the so-called ‘stable’ adducts. (0.08 MB DOC) [file pone.0003394.s015.doc]

**Table S4**. **MD simulations.** RMSD (Å) and radius of gyration (Å) of the so-called ‘stable’ adducts.

|  | | RMSD | | Radius of Gyration | |
| --- | --- | --- | --- | --- | --- |
|  |  | Average | Std | Average | Std |
| C-1 | DCH | 5.6 | 0.4 | 17.5 | 0.5 |
|  | DHI | 7.2 | 0.5 | 17.1 | 0.4 |
|  | DOP | 5.6 | 0.5 | 16.7 | 0.2 |
|  | DOP-H | 6.1 | 0.4 | 16.5 | 0.3 |
|  | DQ | 7.0 | 0.6 | 18.4 | 0.2 |
|  | IQ | 5.8 | 0.2 | 18.0 | 0.4 |
|  | LEUK | 9.9 | 0.3 | 16.1 | 0.2 |
| C-2 | DOP-H | 10.0 | 0.5 | 20.4 | 0.4 |
| C-3 | DOP | 8.8 | 0.6 | 20.5 | 0.3 |
|  | DQ | 10.1 | 0.3 | 18.3 | 0.3 |
|  | IQ | 8.2 | 1.0 | 19.5 | 0.2 |
| C-4 | DCH | 10.2 | 1.5 | 19.9 | 0.5 |
|  | DHI | 10.4 | 0.4 | 17.6 | 0.2 |
|  | DOP | 7.9 | 0.7 | 20.1 | 0.9 |
|  | DOP-H | 10.8 | 1.1 | 20.1 | 0.6 |
|  | DQ | 9.1 | 0.8 | 22.5 | 0.7 |
|  | IQ | 7.5 | 0.4 | 23.0 | 0.8 |
| C-5 | DCH | 7.6 | 0.6 | 24.7 | 0.4 |
|  | DOP-H | 6.9 | 0.3 | 25.8 | 0.5 |
|  | IQ | 11.1 | 0.5 | 24.5 | 1.0 |
| C-6 | DCH | 13.1 | 0.6 | 27.1 | 0.7 |
|  | DHI | 6.2 | 0.5 | 27.4 | 0.4 |
|  | DOP | 8.0 | 1.0 | 25.2 | 1.5 |
|  | DOP-H | 12.1 | 0.8 | 25.6 | 1.8 |
|  | DQ | 8.7 | 0.5 | 24.8 | 0.7 |
|  | IQ | 7.2 | 1.2 | 26.1 | 0.4 |
| MD  Derived | DCH | 3.8 | 0.3 | 15.3 | 0.1 |
|  | DHI | 3.6 | 0.2 | 15.1 | 0.1 |
|  | DOP | 5.7 | 0.8 | 16.3 | 0.4 |
|  | DOP-H | 4.8 | 0.5 | 16.8 | 0.3 |
|  | DQ | 4.0 | 0.3 | 15.3 | 0.2 |
