# Supplementary material for: Inhibition of α-Synuclein Fibrillization by Dopamine Is Mediated by Interactions with Five C-Terminal Residues and with E83 in the NAC Region
Source: PLoS One. 2008 Oct 14;3(10):e3394. doi: 10.1371/journal.pone.0003394 (PMC2566601; doi:10.1371/journal.pone.0003394)
Supplement: Table S5 — MD simulations of dopamine and its derivatives in complex with AS (49 complexes). Distance between the center of mass of dopamine (and its derivatives reported in Figure 1) and that of residues E83, 110–140. The average values (Av.), along with their standard deviations (SD), are reported. (0.57 MB DOC) [file pone.0003394.s016.doc]

| **Representative from cluster 1** (distances in [Å] between the ligands and the C-terminal residues) | | | | | | | | | | | | | | |
| --- | --- | --- | --- | --- | --- | --- | --- | --- | --- | --- | --- | --- | --- | --- |
| Res. Num. | **DCH** | | **DHI** | | **DOP** | | **DOP-H** | | **DQ** | | **IQ** | | **LEUK** | |
|  | Av. | SD | Av. | SD | Av. | SD | Av. | SD | Av. | SD | Av. | SD | Av. | SD |
| **83** | 18.9 | 0.9 | 11.6 | 1.0 | 19.8 | 2.2 | 21.5 | 2.3 | 7.8 | 1.1 | 8.3 | 1.6 | 33.7 | 2.5 |
| **110** | 11.2 | 1.0 | 9.4 | 0.7 | 13.6 | 1.3 | 16.0 | 2.3 | 7.1 | 0.9 | 6.9 | 0.5 | 23.1 | 2.3 |
| **111** | 11.0 | 0.9 | 7.6 | 1.2 | 14.9 | 1.6 | 16.5 | 2.2 | 4.4 | 0.5 | 11.1 | 0.7 | 20.0 | 2.1 |
| **112** | 12.3 | 0.8 | 10.2 | 0.8 | 12.3 | 2.9 | 19.4 | 2.2 | 6.6 | 0.7 | 12.3 | 1.3 | 22.1 | 2.1 |
| **113** | 7.0 | 0.9 | 12.6 | 0.9 | 11.3 | 1.5 | 16.2 | 2.5 | 8.2 | 0.8 | 12.8 | 0.7 | 18.3 | 2.4 |
| **114** | 10.4 | 0.8 | 16.6 | 0.8 | 14.3 | 1.6 | 18.1 | 1.9 | 12.5 | 0.8 | 16.6 | 1.1 | 20.1 | 2.6 |
| **115** | 11.2 | 0.7 | 19.8 | 1.2 | 14.1 | 1.3 | 15.7 | 2.0 | 14.9 | 0.7 | 18.3 | 1.1 | 23.1 | 3.1 |
| **116** | 9.0 | 1.3 | 20.6 | 1.1 | 10.5 | 1.1 | 11.9 | 1.9 | 15.3 | 0.8 | 16.4 | 1.4 | 25.0 | 3.5 |
| **117** | 6.6 | 0.7 | 18.4 | 1.3 | 10.4 | 1.0 | 11.5 | 2.0 | 14.4 | 2.4 | 12.6 | 1.2 | 22.6 | 3.2 |
| **118** | 8.3 | 0.7 | 18.7 | 1.2 | 6.0 | 0.9 | 11.7 | 3.8 | 18.1 | 2.5 | 12.0 | 0.8 | 25.4 | 2.7 |
| **119** | 8.5 | 0.8 | 15.4 | 0.9 | 6.7 | 1.0 | 10.3 | 3.5 | 17.8 | 4.2 | 8.7 | 0.8 | 24.4 | 2.5 |
| **120** | 12.4 | 0.8 | 16.2 | 1.5 | 5.4 | 0.5 | 9.3 | 3.3 | 21.0 | 4.5 | 9.9 | 0.6 | 28.1 | 2.6 |
| **121** | 13.2 | 0.8 | 16.8 | 0.8 | 9.6 | 1.0 | 9.3 | 2.6 | 20.8 | 4.5 | 9.6 | 1.4 | 26.0 | 2.8 |
| **122** | 9.3 | 0.8 | 16.3 | 0.6 | 9.4 | 1.1 | 8.7 | 1.7 | 17.2 | 3.3 | 11.1 | 2.1 | 21.5 | 2.9 |
| **123** | 12.7 | 0.8 | 21.2 | 0.7 | 11.8 | 1.8 | 6.2 | 1.4 | 20.0 | 2.3 | 15.0 | 2.1 | 22.3 | 3.5 |
| **124** | 8.9 | 0.9 | 19.3 | 1.0 | 11.9 | 2.1 | 9.9 | 1.4 | 18.9 | 2.3 | 15.7 | 2.7 | 18.3 | 3.4 |
| **125** | 4.7 | 0.6 | 14.5 | 1.3 | 13.1 | 1.6 | 13.5 | 2.5 | 16.0 | 4.0 | 15.0 | 2.0 | 13.3 | 3.2 |
| **126** | 8.0 | 1.1 | 18.6 | 1.2 | 16.3 | 2.4 | 13.6 | 1.7 | 20.2 | 2.9 | 18.4 | 2.1 | 13.6 | 3.2 |
| **127** | 8.0 | 1.0 | 14.6 | 1.3 | 18.4 | 1.7 | 16.3 | 2.1 | 18.8 | 3.0 | 20.4 | 1.7 | 12.6 | 2.5 |
| **128** | 7.4 | 0.8 | 16.9 | 1.7 | 18.7 | 1.7 | 19.9 | 1.7 | 18.0 | 2.9 | 20.7 | 1.1 | 8.4 | 2.6 |
| **129** | 5.3 | 0.8 | 14.4 | 1.9 | 18.0 | 1.0 | 20.5 | 1.8 | 14.4 | 2.7 | 18.4 | 1.0 | 8.9 | 1.8 |
| **130** | 9.6 | 0.8 | 17.3 | 2.8 | 21.6 | 0.8 | 25.1 | 1.8 | 15.9 | 2.6 | 22.2 | 1.1 | 10.4 | 2.0 |
| **131** | 9.8 | 0.6 | 19.2 | 1.8 | 22.3 | 1.0 | 26.8 | 1.8 | 14.7 | 1.2 | 24.0 | 0.9 | 7.4 | 2.2 |
| **132** | 6.8 | 0.7 | 16.6 | 0.8 | 18.9 | 1.1 | 23.2 | 1.8 | 12.3 | 0.8 | 20.7 | 0.9 | 5.8 | 2.1 |
| **133** | 6.6 | 1.3 | 14.3 | 1.4 | 13.8 | 1.4 | 18.1 | 2.0 | 12.3 | 0.8 | 16.3 | 1.2 | 8.1 | 3.1 |
| **134** | 7.8 | 0.4 | 11.5 | 0.7 | 16.3 | 0.9 | 21.0 | 2.3 | 7.2 | 0.4 | 15.0 | 0.8 | 10.0 | 1.9 |
| **135** | 7.2 | 0.8 | 7.0 | 0.3 | 13.6 | 0.8 | 19.0 | 2.2 | 5.4 | 1.0 | 10.2 | 0.8 | 13.4 | 2.1 |
| **136** | 6.4 | 0.6 | 9.9 | 0.5 | 11.9 | 0.8 | 17.6 | 1.8 | 7.1 | 1.3 | 12.1 | 1.6 | 16.5 | 2.1 |
| **137** | 11.6 | 1.3 | 5.8 | 0.3 | 17.7 | 0.9 | 22.5 | 1.8 | 6.2 | 0.9 | 13.6 | 1.0 | 16.4 | 1.5 |
| **138** | 13.3 | 0.9 | 8.4 | 0.6 | 20.1 | 0.9 | 26.1 | 2.1 | 9.2 | 0.6 | 18.1 | 1.0 | 14.8 | 1.3 |
| **139** | 15.3 | 1.4 | 8.3 | 1.0 | 21.5 | 1.7 | 27.4 | 2.7 | 10.0 | 0.7 | 19.9 | 1.0 | 14.1 | 1.5 |
| **140** | 18.2 | 1.1 | 7.9 | 1.9 | 25.2 | 1.4 | 30.6 | 2.3 | 9.3 | 1.2 | 20.8 | 1.6 | 16.7 | 1.7 |
| **Representative from cluster 2** (distances in [Å] between the ligands and the C-terminal residues) | | | | | | | | | | | | | | |
| Res. Num. | **DCH** | | **DHI** | | **DOP** | | **DOP-H** | | **DQ** | | **IQ** | | **LEUK** | |
|  | Av. | SD | Av. | SD | Av. | SD | Av. | SD | Av. | SD | Av. | SD | Av. | SD |
| **83** | 34.1 | 4.8 | 18.2 | 1.7 | 28.7 | 6.9 | 37.4 | 3.8 | 29.2 | 5.2 | 18.3 | 2.1 | 11.2 | 1.7 |
| **110** | 56.9 | 21.7 | 27.6 | 5.0 | 39.2 | 7.2 | 29.0 | 6.2 | 36.6 | 7.1 | 33.2 | 2.0 | 31.0 | 2.7 |
| **111** | 57.8 | 21.9 | 27.2 | 5.4 | 37.3 | 7.3 | 26.3 | 7.0 | 34.6 | 7.9 | 35.0 | 2.7 | 32.0 | 2.5 |
| **112** | 55.0 | 22.3 | 23.4 | 5.9 | 33.2 | 7.3 | 26.1 | 6.6 | 31.3 | 7.4 | 32.6 | 3.8 | 29.1 | 2.9 |
| **113** | 55.2 | 24.4 | 21.2 | 4.8 | 33.5 | 7.2 | 22.7 | 5.4 | 30.6 | 8.8 | 31.3 | 3.6 | 25.6 | 2.2 |
| **114** | 58.9 | 23.7 | 25.7 | 4.4 | 37.0 | 7.3 | 22.9 | 5.6 | 33.9 | 10.6 | 36.1 | 3.7 | 28.9 | 2.0 |
| **115** | 59.8 | 25.5 | 25.1 | 3.8 | 33.2 | 7.4 | 18.6 | 5.5 | 32.2 | 11.2 | 34.7 | 3.2 | 27.6 | 1.5 |
| **116** | 58.0 | 27.8 | 21.2 | 3.6 | 30.0 | 7.1 | 20.3 | 4.3 | 31.2 | 9.7 | 31.4 | 3.9 | 22.9 | 1.5 |
| **117** | 60.2 | 28.5 | 21.7 | 3.4 | 28.9 | 7.1 | 17.7 | 3.6 | 30.3 | 10.6 | 30.0 | 2.9 | 24.2 | 1.7 |
| **118** | 61.2 | 29.3 | 24.0 | 2.6 | 32.1 | 7.1 | 20.2 | 3.4 | 34.0 | 10.1 | 31.6 | 2.6 | 23.2 | 1.7 |
| **119** | 61.6 | 31.9 | 21.1 | 3.5 | 30.2 | 6.8 | 20.7 | 2.8 | 35.0 | 9.3 | 28.1 | 3.0 | 24.1 | 2.3 |
| **120** | 63.9 | 31.1 | 24.1 | 3.9 | 30.7 | 5.4 | 24.1 | 2.8 | 38.2 | 9.1 | 28.4 | 2.8 | 23.6 | 2.8 |
| **121** | 63.1 | 32.9 | 23.1 | 3.1 | 29.1 | 4.8 | 23.9 | 2.9 | 36.8 | 8.5 | 29.1 | 2.8 | 23.0 | 2.1 |
| **122** | 63.9 | 34.3 | 20.6 | 3.8 | 28.8 | 4.6 | 21.6 | 2.8 | 36.0 | 7.7 | 25.9 | 3.5 | 23.1 | 2.6 |
| **123** | 60.8 | 35.1 | 20.9 | 2.0 | 24.6 | 4.6 | 17.4 | 2.9 | 33.0 | 8.7 | 24.2 | 2.3 | 20.7 | 2.4 |
| **124** | 59.0 | 34.6 | 18.0 | 1.7 | 22.3 | 4.6 | 18.1 | 2.7 | 30.0 | 8.6 | 22.3 | 3.7 | 17.3 | 2.5 |
| **125** | 56.7 | 33.9 | 20.0 | 1.9 | 20.4 | 5.8 | 13.9 | 2.6 | 29.8 | 10.1 | 18.9 | 2.4 | 19.1 | 2.1 |
| **126** | 54.3 | 35.5 | 18.1 | 3.5 | 17.5 | 4.9 | 18.0 | 2.5 | 26.8 | 10.4 | 18.0 | 4.8 | 15.0 | 2.8 |
| **127** | 52.8 | 36.1 | 19.8 | 3.3 | 16.8 | 5.5 | 15.1 | 2.8 | 27.6 | 12.7 | 17.6 | 4.2 | 15.7 | 3.0 |
| **128** | 50.5 | 34.6 | 17.4 | 2.9 | 14.2 | 6.5 | 13.0 | 2.7 | 25.9 | 13.5 | 17.1 | 2.2 | 16.6 | 2.6 |
| **129** | 51.9 | 33.2 | 17.9 | 2.3 | 17.4 | 7.1 | 5.4 | 0.7 | 25.4 | 13.4 | 19.3 | 2.1 | 20.2 | 2.8 |
| **130** | 50.8 | 34.7 | 19.4 | 2.4 | 16.1 | 6.4 | 10.9 | 3.0 | 24.6 | 14.3 | 21.7 | 3.0 | 22.8 | 3.2 |
| **131** | 50.7 | 33.1 | 23.3 | 2.4 | 17.8 | 6.4 | 10.3 | 4.4 | 22.4 | 13.6 | 20.4 | 3.4 | 21.1 | 3.2 |
| **132** | 50.9 | 30.8 | 23.0 | 2.9 | 19.6 | 7.3 | 9.4 | 3.6 | 22.7 | 12.5 | 16.9 | 2.9 | 18.3 | 3.1 |
| **133** | 50.8 | 30.7 | 20.4 | 2.4 | 22.0 | 7.9 | 12.3 | 2.9 | 22.3 | 10.4 | 14.3 | 2.3 | 17.8 | 3.3 |
| **134** | 52.2 | 27.1 | 23.5 | 2.3 | 23.9 | 7.3 | 8.6 | 1.9 | 23.2 | 10.2 | 18.4 | 2.3 | 23.6 | 2.9 |
| **135** | 50.9 | 25.5 | 26.2 | 3.3 | 27.7 | 7.8 | 6.8 | 1.8 | 23.1 | 8.5 | 16.6 | 2.6 | 25.9 | 3.4 |
| **136** | 52.1 | 23.5 | 27.4 | 3.6 | 28.6 | 7.3 | 8.8 | 1.5 | 26.1 | 6.4 | 21.5 | 2.4 | 26.7 | 4.0 |
| **137** | 50.4 | 22.3 | 30.2 | 3.7 | 33.8 | 7.8 | 5.8 | 1.2 | 26.1 | 7.5 | 21.6 | 3.1 | 30.8 | 4.8 |
| **138** | 51.6 | 20.0 | 32.7 | 3.7 | 36.4 | 7.7 | 7.7 | 1.8 | 28.4 | 8.8 | 24.3 | 2.8 | 32.5 | 5.7 |
| **139** | 51.8 | 20.9 | 33.3 | 3.6 | 39.7 | 7.1 | 11.5 | 2.5 | 27.0 | 10.0 | 27.6 | 3.4 | 35.6 | 5.1 |
| **140** | 51.2 | 18.9 | 34.6 | 3.9 | 41.8 | 7.8 | 12.8 | 2.7 | 29.2 | 10.4 | 25.8 | 3.4 | 38.3 | 5.7 |
| **Representative from cluster 3** (distances in [Å] between the ligands and the C-terminal residues) | | | | | | | | | | | | | | |
| Res. Num. | **DCH** | | **DHI** | | **DOP** | | **DOP-H** | | **DQ** | | **IQ** | | **LEUK** | |
|  | Av. | SD | Av. | SD | Av. | SD | Av. | SD | Av. | SD | Av. | SD | Av. | SD |
| **83** | 21.8 | 3.1 | 24.8 | 4.2 | 25.4 | 6.0 | 17.6 | 2.6 | 11.6 | 2.8 | 21.3 | 4.1 | 26.3 | 6.7 |
| **110** | 37.5 | 2.8 | 34.0 | 5.2 | 26.5 | 2.4 | 24.7 | 6.2 | 31.9 | 2.8 | 42.6 | 4.2 | 12.6 | 6.9 |
| **111** | 39.1 | 3.9 | 30.9 | 5.2 | 25.3 | 2.4 | 25.7 | 5.7 | 34.0 | 3.0 | 44.8 | 3.8 | 12.2 | 4.8 |
| **112** | 38.4 | 4.2 | 27.9 | 5.5 | 21.6 | 2.6 | 23.6 | 5.9 | 36.3 | 3.1 | 46.6 | 3.9 | 11.7 | 3.2 |
| **113** | 35.5 | 5.6 | 25.2 | 4.8 | 23.0 | 2.5 | 23.0 | 6.1 | 33.1 | 3.2 | 41.9 | 3.8 | 13.8 | 2.9 |
| **114** | 33.3 | 5.1 | 22.5 | 5.7 | 18.1 | 3.1 | 18.0 | 6.1 | 35.8 | 3.1 | 43.8 | 4.9 | 12.4 | 4.2 |
| **115** | 31.5 | 6.2 | 20.8 | 4.6 | 21.3 | 3.0 | 17.9 | 7.5 | 35.5 | 4.6 | 44.0 | 4.6 | 14.6 | 3.8 |
| **116** | 35.2 | 6.6 | 22.4 | 4.2 | 21.9 | 2.5 | 14.9 | 7.2 | 37.1 | 4.4 | 44.5 | 3.5 | 16.1 | 6.3 |
| **117** | 35.1 | 7.2 | 20.2 | 4.2 | 19.7 | 2.7 | 12.6 | 6.4 | 35.4 | 4.8 | 41.6 | 3.4 | 14.3 | 7.0 |
| **118** | 38.3 | 5.9 | 22.0 | 4.8 | 16.4 | 2.8 | 14.4 | 4.5 | 36.4 | 3.3 | 41.4 | 2.2 | 16.5 | 8.4 |
| **119** | 38.2 | 4.2 | 22.4 | 5.0 | 16.4 | 2.1 | 12.4 | 3.2 | 36.7 | 4.2 | 40.3 | 2.0 | 16.2 | 8.9 |
| **120** | 39.0 | 4.5 | 21.3 | 4.8 | 13.3 | 2.7 | 13.2 | 2.9 | 36.2 | 4.2 | 41.0 | 3.2 | 17.5 | 9.2 |
| **121** | 41.1 | 3.8 | 24.6 | 5.4 | 12.8 | 2.7 | 12.0 | 2.6 | 35.7 | 3.8 | 42.0 | 3.3 | 16.2 | 8.5 |
| **122** | 43.4 | 4.0 | 26.0 | 5.4 | 16.1 | 2.6 | 11.5 | 4.0 | 37.2 | 4.5 | 44.8 | 3.1 | 19.2 | 8.3 |
| **123** | 42.0 | 3.7 | 27.0 | 6.4 | 14.4 | 2.5 | 9.8 | 2.5 | 34.6 | 4.9 | 41.9 | 3.0 | 19.9 | 7.5 |
| **124** | 38.3 | 3.8 | 23.3 | 6.3 | 13.6 | 2.8 | 8.2 | 2.9 | 32.3 | 6.2 | 44.1 | 3.3 | 20.2 | 8.6 |
| **125** | 34.1 | 3.3 | 23.1 | 6.6 | 12.5 | 3.1 | 8.1 | 2.3 | 28.9 | 5.8 | 40.0 | 3.3 | 25.4 | 9.2 |
| **126** | 37.5 | 3.4 | 19.4 | 5.3 | 11.0 | 2.5 | 10.1 | 2.7 | 34.0 | 6.4 | 44.7 | 4.0 | 25.8 | 9.2 |
| **127** | 37.1 | 2.9 | 20.2 | 6.5 | 7.8 | 2.4 | 12.9 | 2.5 | 34.9 | 5.4 | 41.8 | 4.3 | 25.8 | 9.1 |
| **128** | 34.0 | 2.8 | 18.7 | 6.9 | 7.5 | 2.2 | 14.0 | 3.5 | 33.6 | 4.7 | 38.7 | 4.6 | 28.9 | 9.0 |
| **129** | 36.3 | 3.7 | 17.9 | 6.0 | 10.5 | 1.7 | 14.8 | 3.8 | 31.2 | 4.9 | 35.5 | 3.7 | 27.1 | 9.4 |
| **130** | 35.5 | 4.7 | 18.4 | 6.1 | 9.3 | 2.4 | 18.1 | 4.6 | 29.7 | 4.4 | 33.0 | 4.9 | 29.6 | 8.9 |
| **131** | 37.5 | 5.1 | 21.4 | 5.9 | 10.3 | 2.3 | 19.5 | 3.8 | 31.2 | 4.8 | 35.1 | 6.0 | 27.6 | 8.9 |
| **132** | 40.2 | 4.4 | 22.2 | 5.5 | 11.5 | 2.2 | 17.4 | 3.6 | 32.8 | 4.1 | 38.0 | 5.0 | 24.7 | 9.3 |
| **133** | 39.9 | 3.3 | 22.7 | 5.6 | 10.6 | 2.4 | 15.4 | 3.6 | 35.4 | 4.6 | 39.3 | 3.8 | 20.8 | 9.6 |
| **134** | 42.2 | 4.1 | 23.5 | 6.7 | 14.8 | 2.6 | 20.2 | 4.0 | 37.1 | 3.8 | 34.9 | 4.7 | 23.1 | 8.4 |
| **135** | 44.6 | 4.0 | 26.9 | 5.5 | 14.7 | 3.2 | 21.0 | 3.5 | 39.1 | 4.1 | 36.6 | 3.8 | 20.0 | 8.2 |
| **136** | 45.7 | 3.7 | 26.6 | 6.1 | 15.2 | 2.5 | 19.4 | 3.0 | 38.1 | 4.4 | 34.2 | 3.5 | 17.1 | 8.8 |
| **137** | 48.4 | 3.4 | 29.8 | 6.6 | 18.2 | 2.7 | 24.0 | 3.2 | 38.2 | 4.9 | 38.4 | 3.2 | 18.9 | 7.5 |
| **138** | 50.8 | 3.8 | 32.0 | 6.4 | 21.3 | 2.7 | 24.8 | 3.2 | 40.5 | 5.0 | 40.7 | 2.7 | 17.6 | 5.6 |
| **139** | 50.8 | 4.1 | 32.6 | 7.8 | 22.2 | 3.7 | 26.7 | 3.6 | 42.2 | 4.5 | 44.3 | 3.2 | 21.0 | 5.7 |
| **140** | 53.1 | 4.5 | 35.2 | 7.9 | 25.3 | 3.4 | 30.3 | 3.6 | 43.9 | 5.4 | 46.4 | 2.2 | 22.8 | 4.5 |
| **Representative from cluster 4** (distances in [Å] between the ligands and the C-terminal residues) | | | | | | | | | | | | | | |
| Res. Num. | **DCH** | | **DHI** | | **DOP** | | **DOP-H** | | **DQ** | | **IQ** | | **LEUK** | |
|  | Av. | SD | Av. | SD | Av. | SD | Av. | SD | Av. | SD | Av. | SD | Av. | SD |
| **83** | 15.4 | 0.9 | 11.8 | 1.5 | 24.0 | 1.6 | 29.3 | 1.6 | 23.3 | 1.2 | 17.5 | 1.0 | 12.1 | 1.2 |
| **110** | 17.8 | 1.6 | 15.3 | 1.4 | 22.2 | 1.0 | 30.4 | 1.3 | 18.5 | 0.7 | 19.4 | 1.1 | 12.2 | 3.1 |
| **111** | 15.5 | 0.8 | 15.3 | 0.9 | 19.2 | 0.8 | 27.5 | 1.4 | 14.5 | 0.8 | 18.6 | 1.3 | 14.6 | 3.7 |
| **112** | 12.9 | 1.3 | 13.3 | 0.8 | 14.7 | 0.8 | 24.6 | 1.3 | 10.3 | 0.7 | 15.5 | 1.4 | 15.7 | 3.8 |
| **113** | 11.0 | 0.7 | 10.2 | 1.0 | 14.4 | 1.1 | 21.8 | 1.3 | 14.3 | 0.8 | 12.7 | 1.2 | 10.7 | 4.7 |
| **114** | 6.3 | 0.9 | 6.6 | 0.9 | 10.0 | 0.9 | 17.4 | 1.3 | 12.4 | 0.6 | 8.5 | 1.5 | 12.9 | 4.1 |
| **115** | 9.4 | 0.9 | 7.7 | 0.8 | 9.7 | 2.1 | 18.5 | 1.2 | 15.4 | 0.9 | 8.4 | 1.1 | 12.0 | 5.1 |
| **116** | 5.8 | 0.8 | 5.3 | 1.3 | 9.1 | 1.3 | 15.5 | 1.3 | 16.8 | 0.7 | 5.5 | 0.7 | 13.2 | 3.1 |
| **117** | 8.4 | 0.8 | 8.8 | 1.5 | 10.1 | 2.0 | 17.5 | 1.2 | 19.3 | 1.0 | 8.8 | 1.3 | 11.8 | 2.2 |
| **118** | 5.7 | 0.6 | 8.6 | 2.3 | 14.4 | 1.9 | 12.9 | 1.2 | 23.8 | 0.9 | 9.4 | 2.0 | 9.4 | 1.2 |
| **119** | 9.9 | 0.9 | 11.1 | 1.2 | 16.9 | 1.4 | 14.1 | 0.9 | 26.1 | 0.9 | 9.3 | 3.0 | 12.6 | 1.7 |
| **120** | 10.0 | 1.9 | 13.7 | 1.5 | 19.6 | 1.8 | 13.9 | 1.6 | 28.9 | 1.0 | 12.6 | 2.8 | 14.3 | 2.4 |
| **121** | 13.6 | 1.1 | 14.4 | 0.8 | 21.3 | 1.8 | 13.0 | 1.2 | 31.5 | 0.9 | 11.8 | 2.8 | 17.3 | 2.0 |
| **122** | 13.2 | 1.3 | 12.4 | 0.7 | 18.0 | 2.6 | 10.7 | 1.1 | 29.7 | 1.1 | 11.7 | 2.6 | 16.1 | 1.9 |
| **123** | 9.9 | 1.2 | 7.9 | 0.2 | 15.4 | 1.9 | 6.7 | 0.7 | 26.0 | 1.0 | 7.4 | 2.2 | 19.2 | 1.0 |
| **124** | 10.8 | 0.7 | 11.3 | 0.8 | 16.0 | 1.3 | 8.2 | 1.0 | 25.0 | 1.2 | 10.5 | 1.4 | 19.2 | 1.8 |
| **125** | 6.4 | 0.7 | 8.0 | 0.6 | 14.1 | 1.7 | 5.5 | 0.7 | 22.2 | 1.1 | 8.1 | 1.7 | 22.4 | 1.9 |
| **126** | 11.0 | 0.9 | 13.5 | 1.1 | 12.5 | 1.1 | 8.7 | 1.3 | 19.5 | 1.2 | 11.4 | 0.9 | 22.7 | 2.8 |
| **127** | 10.0 | 1.5 | 14.2 | 1.2 | 13.1 | 0.9 | 11.1 | 1.7 | 18.8 | 1.6 | 13.5 | 1.4 | 26.3 | 2.7 |
| **128** | 9.5 | 0.8 | 12.6 | 1.4 | 8.6 | 0.8 | 13.1 | 1.6 | 14.5 | 1.2 | 10.1 | 1.6 | 23.7 | 3.0 |
| **129** | 14.1 | 0.7 | 13.8 | 1.3 | 8.3 | 1.0 | 16.5 | 1.5 | 11.7 | 1.1 | 12.1 | 1.7 | 24.8 | 3.8 |
| **130** | 15.1 | 0.9 | 17.4 | 1.4 | 11.0 | 0.6 | 20.4 | 1.9 | 9.4 | 1.2 | 16.0 | 1.8 | 26.3 | 4.4 |
| **131** | 16.4 | 0.9 | 16.7 | 1.1 | 9.0 | 0.5 | 20.9 | 1.6 | 5.6 | 1.0 | 13.9 | 1.7 | 23.1 | 4.7 |
| **132** | 13.1 | 0.9 | 12.9 | 1.0 | 6.2 | 0.4 | 19.1 | 1.4 | 7.5 | 0.8 | 10.6 | 1.6 | 20.4 | 4.3 |
| **133** | 10.0 | 0.7 | 9.5 | 1.4 | 6.3 | 0.4 | 15.9 | 1.3 | 10.9 | 0.9 | 7.5 | 1.6 | 18.7 | 3.1 |
| **134** | 9.6 | 1.1 | 11.3 | 1.3 | 11.9 | 0.6 | 20.5 | 1.4 | 8.4 | 0.7 | 12.4 | 1.6 | 18.6 | 3.6 |
| **135** | 11.1 | 0.7 | 10.9 | 1.5 | 13.3 | 0.5 | 17.1 | 1.4 | 13.6 | 0.8 | 11.0 | 1.6 | 18.5 | 3.0 |
| **136** | 6.3 | 0.5 | 9.6 | 1.5 | 13.4 | 1.3 | 11.6 | 1.3 | 15.5 | 1.0 | 7.2 | 1.5 | 19.6 | 3.0 |
| **137** | 11.2 | 0.8 | 12.3 | 1.1 | 16.4 | 1.0 | 11.6 | 1.1 | 15.8 | 1.3 | 9.8 | 1.6 | 23.7 | 3.1 |
| **138** | 12.7 | 1.5 | 12.6 | 1.6 | 17.2 | 1.7 | 7.5 | 1.1 | 17.0 | 1.5 | 10.3 | 1.9 | 25.2 | 3.4 |
| **139** | 16.3 | 0.9 | 16.9 | 1.5 | 21.3 | 1.5 | 8.4 | 1.4 | 13.6 | 1.8 | 14.8 | 1.9 | 28.0 | 2.9 |
| **140** | 18.4 | 1.8 | 18.8 | 1.8 | 22.9 | 1.5 | 6.7 | 1.3 | 17.4 | 2.3 | 16.9 | 1.8 | 30.3 | 3.9 |
| **Representative from cluster** **5** (distances in [Å] between the ligands and the C-terminal residues) | | | | | | | | | | | | | | |
| Res. Num. | **DCH** | | **DHI** | | **DOP** | | **DOP-H** | | **DQ** | | **IQ** | | **LEUK** | |
|  | Av. | SD | Av. | SD | Av. | SD | Av. | SD | Av. | SD | Av. | SD | Av. | SD |
| **83** | 16.5 | 2.7 | 35.0 | 8.6 | 12.0 | 2.3 | 26.6 | 2.6 | 50.6 | 7.0 | 23.2 | 3.4 | 21.7 | 3.4 |
| **110** | 14.9 | 0.7 | 22.6 | 11.2 | 29.8 | 1.9 | 11.6 | 0.7 | 24.7 | 12.7 | 11.7 | 2.1 | 13.2 | 1.5 |
| **111** | 11.9 | 0.6 | 19.7 | 10.9 | 29.2 | 1.9 | 10.3 | 0.6 | 24.8 | 13.3 | 12.3 | 2.3 | 14.0 | 2.0 |
| **112** | 8.8 | 0.7 | 16.7 | 10.9 | 25.3 | 1.9 | 6.6 | 0.6 | 23.8 | 15.0 | 9.9 | 2.4 | 11.2 | 2.1 |
| **113** | 8.3 | 0.6 | 20.2 | 11.8 | 26.8 | 2.7 | 9.2 | 0.9 | 24.3 | 14.2 | 13.0 | 3.9 | 14.3 | 3.5 |
| **114** | 6.4 | 0.5 | 17.5 | 11.8 | 22.9 | 3.5 | 7.1 | 1.2 | 21.3 | 13.7 | 11.3 | 5.1 | 12.0 | 4.5 |
| **115** | 4.8 | 0.4 | 20.1 | 10.9 | 23.6 | 3.6 | 9.1 | 1.9 | 23.4 | 15.3 | 15.1 | 4.7 | 14.1 | 4.4 |
| **116** | 7.5 | 0.4 | 21.7 | 12.0 | 24.0 | 2.6 | 8.3 | 1.2 | 24.8 | 15.6 | 13.3 | 3.4 | 12.7 | 3.4 |
| **117** | 6.6 | 0.5 | 24.6 | 12.8 | 20.4 | 2.4 | 8.2 | 1.5 | 25.2 | 15.7 | 15.0 | 3.7 | 11.0 | 3.5 |
| **118** | 8.6 | 0.5 | 25.4 | 13.0 | 19.0 | 2.1 | 6.0 | 0.6 | 22.6 | 15.6 | 12.1 | 2.7 | 7.5 | 2.5 |
| **119** | 12.8 | 0.5 | 29.2 | 13.2 | 18.3 | 2.6 | 10.2 | 1.1 | 26.0 | 14.9 | 14.4 | 2.0 | 8.8 | 2.0 |
| **120** | 14.3 | 0.8 | 31.8 | 12.7 | 15.7 | 2.7 | 11.9 | 1.1 | 26.7 | 15.0 | 16.4 | 2.2 | 7.3 | 3.0 |
| **121** | 17.1 | 0.6 | 33.3 | 13.2 | 18.9 | 4.0 | 13.9 | 1.2 | 27.9 | 14.3 | 16.6 | 2.6 | 10.9 | 2.7 |
| **122** | 13.8 | 0.6 | 29.2 | 13.3 | 16.7 | 4.1 | 9.9 | 1.5 | 24.0 | 14.0 | 12.6 | 2.7 | 11.2 | 2.1 |
| **123** | 18.1 | 0.9 | 32.5 | 12.5 | 15.9 | 6.2 | 13.4 | 1.8 | 25.1 | 12.8 | 13.7 | 4.8 | 12.8 | 2.1 |
| **124** | 17.1 | 1.8 | 30.9 | 13.2 | 13.2 | 4.4 | 9.9 | 2.2 | 22.4 | 12.6 | 6.3 | 3.0 | 12.0 | 2.4 |
| **125** | 15.8 | 1.0 | 29.0 | 12.8 | 15.3 | 4.2 | 10.5 | 0.9 | 22.6 | 12.5 | 6.2 | 1.9 | 15.7 | 2.1 |
| **126** | 17.4 | 0.7 | 30.3 | 13.3 | 18.2 | 5.2 | 12.5 | 1.2 | 25.7 | 13.2 | 7.6 | 2.5 | 14.7 | 1.5 |
| **127** | 19.1 | 1.4 | 28.0 | 13.1 | 20.3 | 3.9 | 12.5 | 0.9 | 28.6 | 13.6 | 12.6 | 2.6 | 16.1 | 2.4 |
| **128** | 16.4 | 1.4 | 30.3 | 14.6 | 21.6 | 4.7 | 13.8 | 0.8 | 29.6 | 12.9 | 14.3 | 2.6 | 13.2 | 2.8 |
| **129** | 20.4 | 1.5 | 32.3 | 13.3 | 24.8 | 5.6 | 18.5 | 0.7 | 32.4 | 12.8 | 17.2 | 2.4 | 15.2 | 2.9 |
| **130** | 21.3 | 2.2 | 34.3 | 14.0 | 28.5 | 5.4 | 20.1 | 2.6 | 34.7 | 12.6 | 19.8 | 2.7 | 15.1 | 4.1 |
| **131** | 24.0 | 1.9 | 35.7 | 12.4 | 30.1 | 6.4 | 22.9 | 2.4 | 38.0 | 12.2 | 23.1 | 2.5 | 18.1 | 4.4 |
| **132** | 21.1 | 1.4 | 34.2 | 11.1 | 26.4 | 6.3 | 21.8 | 1.7 | 38.8 | 12.8 | 22.5 | 2.3 | 19.6 | 3.8 |
| **133** | 25.1 | 1.7 | 32.9 | 8.9 | 30.0 | 6.5 | 25.6 | 2.5 | 41.3 | 11.8 | 25.2 | 3.1 | 23.3 | 2.7 |
| **134** | 25.3 | 1.1 | 32.5 | 10.3 | 26.7 | 6.5 | 23.5 | 2.1 | 37.8 | 11.7 | 25.9 | 3.1 | 23.7 | 4.1 |
| **135** | 29.0 | 0.9 | 31.5 | 8.6 | 30.3 | 6.1 | 27.6 | 2.0 | 37.7 | 11.5 | 28.4 | 3.7 | 26.7 | 4.1 |
| **136** | 27.5 | 1.7 | 31.2 | 9.3 | 26.5 | 6.1 | 23.3 | 2.3 | 33.8 | 11.5 | 29.8 | 4.0 | 27.2 | 5.5 |
| **137** | 23.3 | 1.1 | 31.8 | 8.0 | 25.2 | 5.9 | 21.6 | 2.3 | 34.2 | 11.8 | 31.6 | 3.3 | 29.4 | 4.7 |
| **138** | 23.5 | 2.1 | 33.1 | 6.0 | 27.4 | 6.2 | 24.4 | 2.6 | 34.8 | 11.0 | 31.9 | 4.2 | 30.7 | 4.2 |
| **139** | 20.5 | 2.7 | 34.1 | 6.4 | 28.0 | 6.1 | 22.2 | 3.5 | 39.3 | 11.2 | 35.7 | 4.2 | 33.3 | 5.4 |
| **140** | 20.3 | 2.2 | 33.8 | 8.3 | 28.6 | 5.0 | 21.2 | 2.6 | 39.7 | 11.4 | 36.6 | 5.6 | 35.0 | 5.3 |
| **Representative from cluster 6** (distances in [Å] between the ligands and the C-terminal residues) | | | | | | | | | | | | | | |
| Res. Num. | **DCH** | | **DHI** | | **DOP** | | **DOP-H** | | **DQ** | | **IQ** | | **LEUK** | |
|  | Av. | SD | Av. | SD | Av. | SD | Av. | SD | Av. | SD | Av. | SD | Av. | SD |
| **83** | 30.3 | 5.8 | 11.9 | 1.4 | 19.0 | 1.5 | 13.1 | 0.6 | 21.3 | 3.4 | 28.5 | 1.8 | 20.1 | 10.2 |
| **110** | 27.6 | 5.3 | 20.9 | 2.4 | 15.3 | 0.7 | 20.4 | 2.9 | 18.7 | 2.4 | 16.3 | 1.1 | 26.3 | 13.8 |
| **111** | 23.8 | 5.6 | 17.2 | 2.3 | 12.4 | 0.7 | 20.7 | 1.3 | 14.8 | 2.2 | 19.5 | 1.0 | 25.1 | 13.0 |
| **112** | 23.0 | 4.9 | 14.8 | 3.3 | 11.6 | 0.5 | 18.3 | 1.3 | 13.8 | 2.1 | 15.8 | 1.0 | 23.0 | 12.9 |
| **113** | 18.0 | 4.3 | 12.2 | 1.1 | 6.0 | 0.7 | 20.8 | 1.4 | 11.7 | 2.9 | 19.7 | 1.3 | 19.8 | 13.4 |
| **114** | 19.2 | 3.5 | 14.0 | 1.4 | 10.2 | 0.5 | 14.9 | 1.5 | 11.5 | 2.1 | 17.8 | 1.6 | 18.2 | 13.0 |
| **115** | 15.9 | 2.3 | 10.4 | 0.7 | 7.2 | 0.8 | 19.4 | 1.5 | 8.6 | 2.4 | 21.4 | 1.9 | 19.3 | 11.5 |
| **116** | 18.3 | 1.3 | 14.2 | 1.1 | 11.7 | 0.9 | 20.7 | 1.6 | 12.9 | 2.2 | 19.2 | 2.5 | 17.8 | 12.9 |
| **117** | 14.7 | 1.4 | 12.7 | 1.1 | 10.6 | 1.3 | 21.3 | 1.4 | 11.8 | 2.0 | 23.9 | 2.4 | 19.0 | 10.5 |
| **118** | 16.1 | 2.9 | 16.8 | 1.5 | 13.9 | 2.0 | 26.0 | 1.3 | 13.2 | 2.1 | 25.2 | 3.1 | 18.7 | 10.8 |
| **119** | 19.8 | 1.8 | 18.2 | 1.1 | 16.0 | 1.7 | 25.6 | 2.2 | 15.7 | 2.5 | 22.5 | 3.0 | 17.9 | 12.8 |
| **120** | 21.7 | 1.1 | 18.1 | 1.8 | 16.4 | 1.4 | 23.6 | 2.9 | 16.8 | 2.5 | 19.3 | 2.5 | 17.4 | 14.3 |
| **121** | 22.8 | 1.2 | 19.5 | 1.6 | 19.3 | 1.0 | 25.6 | 3.2 | 20.5 | 2.3 | 23.3 | 2.1 | 19.9 | 13.2 |
| **122** | 18.1 | 1.8 | 15.9 | 1.0 | 15.7 | 1.1 | 24.3 | 1.9 | 18.8 | 3.0 | 25.4 | 2.3 | 20.0 | 10.8 |
| **123** | 18.8 | 1.7 | 16.1 | 1.4 | 18.1 | 1.1 | 22.5 | 2.4 | 19.6 | 2.1 | 27.3 | 1.5 | 22.7 | 10.0 |
| **124** | 15.0 | 1.5 | 12.5 | 1.0 | 14.5 | 1.2 | 23.6 | 1.9 | 15.8 | 2.1 | 25.6 | 1.3 | 22.0 | 9.5 |
| **125** | 15.0 | 2.1 | 11.1 | 1.0 | 12.8 | 1.1 | 19.7 | 2.3 | 15.6 | 1.7 | 29.8 | 1.3 | 24.1 | 7.8 |
| **126** | 11.4 | 1.8 | 6.2 | 0.8 | 8.0 | 1.5 | 23.5 | 1.5 | 10.0 | 1.7 | 28.7 | 1.8 | 24.0 | 8.2 |
| **127** | 11.2 | 1.3 | 6.7 | 0.4 | 11.4 | 2.5 | 25.4 | 2.5 | 11.5 | 1.5 | 27.9 | 1.4 | 26.5 | 8.7 |
| **128** | 7.3 | 1.3 | 4.8 | 0.6 | 9.4 | 3.1 | 25.1 | 1.8 | 8.5 | 1.1 | 30.3 | 1.2 | 27.9 | 8.6 |
| **129** | 6.3 | 1.2 | 8.2 | 0.4 | 10.1 | 1.9 | 29.2 | 1.8 | 6.3 | 1.4 | 33.1 | 1.2 | 30.8 | 7.4 |
| **130** | 6.7 | 1.1 | 11.5 | 1.0 | 12.7 | 1.8 | 29.7 | 1.7 | 9.0 | 1.4 | 36.9 | 1.2 | 34.3 | 6.8 |
| **131** | 10.4 | 1.0 | 13.7 | 0.8 | 13.1 | 0.8 | 34.3 | 1.6 | 10.9 | 1.6 | 40.4 | 1.2 | 35.4 | 6.4 |
| **132** | 8.9 | 1.4 | 10.8 | 0.5 | 9.6 | 1.0 | 32.7 | 2.2 | 8.4 | 2.0 | 40.1 | 1.6 | 33.1 | 7.1 |
| **133** | 5.7 | 1.4 | 6.6 | 0.5 | 5.6 | 1.0 | 27.2 | 2.4 | 6.1 | 1.3 | 35.1 | 1.8 | 29.1 | 7.9 |
| **134** | 7.6 | 0.7 | 6.9 | 0.5 | 7.0 | 0.8 | 30.2 | 2.5 | 10.4 | 1.3 | 37.5 | 1.4 | 31.7 | 8.0 |
| **135** | 6.0 | 0.6 | 5.5 | 0.4 | 6.3 | 0.4 | 26.1 | 2.4 | 8.4 | 1.4 | 34.5 | 2.1 | 29.8 | 8.4 |
| **136** | 11.1 | 0.7 | 10.5 | 0.5 | 11.9 | 0.8 | 30.1 | 2.5 | 6.9 | 2.3 | 37.1 | 2.0 | 31.4 | 9.4 |
| **137** | 10.3 | 2.0 | 7.1 | 1.0 | 7.5 | 0.7 | 25.8 | 2.1 | 9.2 | 1.7 | 31.0 | 1.8 | 32.8 | 9.4 |
| **138** | 12.5 | 1.8 | 10.6 | 0.8 | 11.7 | 0.7 | 27.9 | 2.2 | 12.6 | 1.5 | 30.8 | 1.4 | 32.5 | 9.0 |
| **139** | 14.0 | 2.7 | 9.8 | 1.6 | 12.6 | 0.6 | 24.6 | 2.5 | 13.4 | 1.8 | 29.0 | 1.5 | 32.4 | 10.1 |
| **140** | 12.8 | 3.7 | 11.6 | 2.4 | 12.7 | 1.0 | 22.2 | 4.1 | 16.6 | 1.7 | 32.7 | 1.6 | 34.3 | 9.9 |
| **MD-Derived** (distances in [Å] between the ligands and the C-terminal residues) | | | | | | | | | | | | | | |
| Res. Num. | **DCH** | | **DHI** | | **DOP** | | **DOP-H** | | **DQ** | | **IQ** | | **LEUK** | |
|  | Av. | SD | Av. | SD | Av. | SD | Av. | SD | Av. | SD | Av. | SD | Av. | SD |
| **83** | 27.4 | 2.9 | 12.1 | 1.5 | 28.7 | 5.8 | 26.7 | 2.4 | 17.6 | 1.1 | 24.7 | 3.3 | 19.9 | 2.6 |
| **110** | 14.2 | 3.05 | 24.0 | 2.8 | 31.4 | 1.8 | 6.2 | 1.4 | 16.5 | 1.3 | 19.3 | 10.1 | 15.3 | 2.6 |
| **111** | 12.4 | 3.42 | 25.0 | 2.2 | 30.9 | 2.1 | 4.9 | 1.8 | 12.6 | 1.5 | 18.4 | 11.9 | 16.1 | 2.6 |
| **112** | 8.9 | 3.39 | 22.5 | 1.9 | 27.0 | 2.2 | 9.3 | 1.9 | 12.3 | 1.0 | 16.9 | 14.1 | 18.8 | 2.6 |
| **113** | 10.0 | 2.08 | 19.7 | 2.4 | 25.7 | 1.8 | 10.0 | 1.5 | 14.3 | 1.1 | 18.5 | 13.1 | 19.6 | 2.5 |
| **114** | 13.6 | 1.26 | 24.9 | 2.6 | 31.1 | 1.9 | 5.9 | 0.8 | 13.8 | 1.3 | 21.8 | 12.9 | 21.2 | 2.8 |
| **115** | 11.8 | 1.49 | 26.8 | 2.3 | 31.8 | 2.4 | 10.0 | 1.7 | 10.0 | 1.5 | 21.1 | 14.9 | 23.6 | 2.6 |
| **116** | 8.6 | 1.62 | 24.1 | 2.6 | 29.0 | 2.7 | 12.4 | 1.6 | 6.9 | 1.3 | 19.1 | 15.4 | 23.1 | 2.5 |
| **117** | 10.3 | 3.58 | 24.9 | 2.6 | 28.2 | 2.3 | 15.5 | 1.5 | 7.4 | 0.9 | 22.3 | 15.8 | 27.1 | 2.6 |
| **118** | 10.4 | 2.36 | 21.1 | 2.3 | 24.8 | 2.1 | 14.6 | 1.7 | 5.1 | 0.8 | 22.3 | 13.9 | 25.2 | 2.7 |
| **119** | 11.8 | 4.29 | 20.7 | 1.7 | 22.3 | 2.1 | 19.4 | 1.6 | 8.6 | 0.8 | 24.6 | 14.9 | 29.3 | 2.6 |
| **120** | 14.0 | 3.44 | 18.1 | 1.9 | 20.6 | 2.1 | 22.2 | 1.7 | 6.5 | 1.3 | 25.8 | 13.7 | 30.2 | 2.6 |
| **121** | 12.8 | 4.27 | 19.1 | 1.8 | 18.2 | 2.4 | 23.0 | 1.8 | 6.5 | 1.1 | 25.4 | 15.5 | 31.5 | 2.5 |
| **122** | 9.0 | 2.92 | 19.1 | 1.5 | 19.9 | 2.4 | 18.4 | 1.8 | 10.7 | 1.5 | 21.1 | 15.6 | 26.7 | 2.6 |
| **123** | 9.6 | 1.63 | 16.7 | 1.7 | 16.1 | 2.6 | 21.8 | 1.8 | 10.9 | 1.3 | 20.7 | 15.7 | 27.4 | 2.4 |
| **124** | 7.4 | 1.76 | 17.6 | 1.4 | 20.2 | 2.3 | 17.2 | 1.7 | 10.6 | 0.7 | 17.9 | 15.1 | 23.1 | 2.5 |
| **125** | 10.6 | 0.95 | 16.5 | 2.2 | 21.6 | 1.8 | 16.3 | 1.5 | 11.7 | 0.9 | 20.7 | 12.8 | 23.1 | 2.6 |
| **126** | 12.4 | 1.28 | 13.7 | 1.8 | 17.7 | 1.8 | 20.8 | 1.6 | 14.3 | 1.3 | 22.6 | 12.8 | 26.2 | 2.5 |
| **127** | 13.1 | 1.32 | 12.0 | 1.7 | 13.4 | 1.8 | 24.1 | 1.6 | 16.5 | 1.5 | 22.4 | 13.2 | 27.5 | 2.4 |
| **128** | 16.7 | 1.75 | 11.0 | 2.4 | 10.8 | 1.7 | 27.9 | 1.6 | 20.1 | 1.2 | 26.4 | 12.7 | 31.0 | 2.4 |
| **129** | 19.7 | 1.34 | 6.8 | 2.6 | 11.1 | 2.1 | 29.9 | 1.7 | 23.3 | 1.5 | 26.8 | 11.2 | 29.4 | 2.7 |
| **130** | 21.3 | 1.58 | 10.0 | 3.8 | 7.7 | 1.9 | 32.9 | 1.6 | 20.6 | 1.9 | 28.9 | 11.1 | 33.0 | 2.2 |
| **131** | 17.2 | 1.57 | 12.1 | 3.3 | 8.9 | 2.4 | 29.0 | 1.7 | 18.5 | 1.4 | 26.1 | 12.6 | 32.0 | 2.6 |
| **132** | 15.9 | 1.86 | 10.1 | 2.3 | 11.9 | 1.9 | 26.8 | 1.6 | 22.1 | 1.2 | 22.8 | 11.7 | 27.6 | 2.6 |
| **133** | 20.7 | 2.59 | 7.4 | 1.8 | 11.9 | 2.5 | 29.1 | 1.1 | 22.0 | 1.7 | 24.0 | 9.7 | 25.5 | 2.4 |
| **134** | 19.1 | 2.11 | 13.1 | 3.0 | 10.6 | 3.4 | 30.8 | 1.9 | 18.7 | 1.5 | 24.8 | 11.7 | 29.3 | 2.5 |
| **135** | 14.2 | 2.94 | 14.2 | 1.9 | 14.7 | 2.7 | 25.5 | 1.8 | 21.1 | 1.3 | 21.0 | 12.6 | 26.1 | 2.6 |
| **136** | 16.1 | 4.21 | 11.6 | 1.8 | 17.6 | 2.4 | 24.2 | 1.9 | 25.2 | 1.3 | 20.0 | 9.5 | 20.6 | 2.6 |
| **137** | 20.2 | 3.87 | 9.3 | 1.9 | 16.5 | 2.7 | 29.9 | 2.0 | 26.6 | 1.3 | 23.3 | 7.6 | 24.5 | 2.4 |
| **138** | 20.8 | 4.01 | 8.4 | 2.2 | 18.2 | 2.7 | 28.7 | 2.0 | 30.9 | 1.3 | 22.2 | 6.3 | 21.1 | 2.3 |
| **139** | 24.9 | 4.73 | 11.1 | 1.5 | 22.4 | 2.8 | 32.8 | 1.8 | 32.5 | 1.3 | 24.2 | 4.3 | 21.8 | 2.0 |
| **140** | 25.5 | 4.15 | 9.8 | 3.1 | 21.2 | 2.9 | 31.9 | 2.2 | 17.6 | 1.1 | 24.1 | 3.7 | 22.1 | 2.3 |

**Table S5**. **MD** **simulations of dopamine and its derivatives in complex with AS (49 complexes).** Distance between the center of mass of dopamine (and its derivatives reported in Figure 1) and that of residues E83, 110-140. The average values (Av.), along with their standard deviations (SD), are reported.
