# Supplementary material for: Inhibition of α-Synuclein Fibrillization by Dopamine Is Mediated by Interactions with Five C-Terminal Residues and with E83 in the NAC Region
Source: PLoS One. 2008 Oct 14;3(10):e3394. doi: 10.1371/journal.pone.0003394 (PMC2566601; doi:10.1371/journal.pone.0003394)
Supplement: Table S6 — Structural and electrostatic similarity across the ligands reported Figure 1. The Tanimoto coefficients characterizing the shape (Ts) and the electrostatic potential (Te) of the ligands reported in Figure 1 are presented. DOP-H is not shown because it is charged, unlike all of the other ligands. (0.04 MB DOC) [file pone.0003394.s017.doc]

**Table S6**. **Structural and electrostatic similarity across the ligands reported Figure 1.** The Tanimoto coefficients characterizing the shape (Ts) and the electrostatic potential (Te) of the ligands reported in Figure 1 are presented. DOP-H is not shown because it is charged, unlike all of the other ligands.

|  | DCH | DHI | DOP | DQ | IQ | LEUK |  |
| --- | --- | --- | --- | --- | --- | --- | --- |
| DCH | 1  1 | 0.99  0.38 | 0.85  0.38 | 0.88  0.82 | 0.99  0.96 | 0.99  0.46 | 🡨 Ts |
|  |  |  |  |  |  |  | 🡨 Te |
| DHI |  | 1  1 | 0.85  0.54 | 0.86  0.37 | 0.99  0.38 | 0.99  0.91 | 🡨 Ts |
|  |  |  |  |  |  |  | 🡨 Te |
| DOP |  |  | 1  1 | 0.99  0.15 | 0.85  0.02 | 0.86  0.51 | 🡨 Ts |
|  |  |  |  |  |  |  | 🡨 Te |
| DQ |  |  |  | 1  1 | 0.86  0.72 | 0.86  0.43 | 🡨 Ts |
|  |  |  |  |  |  |  | 🡨 Te |
| IQ |  |  |  |  | 1  1 | 0.99  0.51 | 🡨 Ts |
|  |  |  |  |  |  |  | 🡨 Te |
| LEUK |  |  |  |  |  | 1  1 | 🡨 Ts |
|  |  |  |  |  |  |  | 🡨 Te |
