# Supplementary material for: Inhibition of α-Synuclein Fibrillization by Dopamine Is Mediated by Interactions with Five C-Terminal Residues and with E83 in the NAC Region
Source: PLoS One. 2008 Oct 14;3(10):e3394. doi: 10.1371/journal.pone.0003394 (PMC2566601; doi:10.1371/journal.pone.0003394)
Supplement: Table S7 — MD simulations of NMR-derived conformations from the cluster analysis of Kelley et al. Hydrogen bonds and hydrophobic contacts for 11 out of the 18 analyzed complexes forming interactions with the protein. Several of then shown interactions with the C-terminal region, including the 125YEMPS129 region. The distance (D) in the hydrogen bond column was measured between the heavy atoms. The hydrophobic contacts were measured as the distance (D) between the center of mass of the ligand and the specific amino acid. (0.04 MB DOC) [file pone.0003394.s018.doc]

**Table S7.** **MD simulations of NMR-derived conformations from the cluster analysis of Kelley et al**. Hydrogen bonds and hydrophobic contacts for 11 out of the 18 analyzed complexes forming interactions with the protein. Several of then shown interactions with the C-terminal region, including the ^125^YEMPS^129^ region. The distance (D) in the hydrogen bond column was measured between the heavy atoms. The hydrophobic contacts were measured as the distance (D) between the center of mass of the ligand and the specific amino acid.

|  |  | Hydrogen Bonds | Hydrophobic contacts |
| --- | --- | --- | --- |
| Repr.  cluster  1  (5 %) | - | - | - |
| Repr.  cluster  2  (5 %) | DOPH | Glu130(OE2)-DOP-H(O1), (D: 2.9±0.5 Å) | Met127 (D: 4.4±0.4 Å) |
|  | DCH | - | Ile112 (D: 6.7±1.1 Å)  Glu126 (D: 7.7±0.5 Å)  Tyr136 (D: 4.3±0.5 Å) |
| Repr.  cluster  3  (4 %) | DOP | Ser129(O)-DOP(O1), (D: 2.7±0.1 Å) | Gln134 (D: 4.4±0.4 Å)  Lys43 (D: 5.2±0.5 Å) |
|  | DCH | - | Glu126 (D: 5.9±1.5 Å)  Gln134 (D: 6.6±1.3 Å) |
| Repr.  cluster  4  (4 %) | DOP | Glu123(OE2)-DOP(O1), (D: 2.6±0.3 Å) | Gln134 (D: 4.7±0.7 Å) |
|  | DOPH | Pro128(O)-DOP-H(O2), (D: 2.8±0.2 Å) | - |
|  | DCH | - | Glu123 (D: 4.3±0.4 Å) |
| Repr.  cluster  5  (4 %) | DOP | Glu131(OE2)-DOP(O2), (D: 3.0±0.9 Å) | Tyr125 (D: 7.3±1.8 Å) |
|  | DCH | - | Thr81 (D: 5.7±0.6 Å)  Pro128 (D: 4.4±0.6 Å) |
| Repr.  cluster  6  (3 %) | DOP-H | Glu126(O)-DOP-H(N1), (D: 2.8±0.1 Å)  Glu126(OE2)-DOP-H(O2), (D: 2.7±0.4 Å) | - |
|  | DCH | - | - Val66 (D: 5.2±1.1 Å)  - Glu126 (D: 4.0±0. 5 Å) |
