# Supplementary material for: Inhibition of α-Synuclein Fibrillization by Dopamine Is Mediated by Interactions with Five C-Terminal Residues and with E83 in the NAC Region
Source: PLoS One. 2008 Oct 14;3(10):e3394. doi: 10.1371/journal.pone.0003394 (PMC2566601; doi:10.1371/journal.pone.0003394)
Supplement: Table S8 — MD simulations of the dopamine forms/AS adducts. The average values (Av.) with their standard deviations (SD) of the distance between the center of mass of E83 along with the C-Terminal residues (from 110 to 140) and dopamine, along the trajectory, are reported here. (0.25 MB DOC) [file pone.0003394.s019.doc]

| **Representative from cluster 1**  (distances in Å between the ligands and the C-terminal residues) | | | | | | | **Representative from cluster 2**  (distances in Å between the ligands and the C-terminal residues) | | | | | | |
| --- | --- | --- | --- | --- | --- | --- | --- | --- | --- | --- | --- | --- | --- |
| **Res. Num.** | **DCH** | | **DOP** | | **DOP-H** | | **Res.**  **Num** | **DCH** | | **DOP** | | **DOP-H** | |
|  | Av. | SD | Av. | SD | Av. | SD |  | Av. | SD | Av. | SD | Av. | SD |
| **83** | 39.9 | 2.6 | 27.0 | 1.9 | 37.0 | 4.3 | **83** | 34.1 | 1.8 | 29.4 | 2.5 | 23.4 | 1.8 |
| **110** | 12.6 | 2.2 | 10.0 | 2.6 | 14.1 | 3.2 | **110** | 12.6 | 0.9 | 6.5 | 1.1 | 13.4 | 0.9 |
| **111** | 13.3 | 2.3 | 13.8 | 2.6 | 11.7 | 3.6 | **111** | 8.3 | 1.0 | 9.7 | 0.8 | 14.2 | 0.6 |
| **112** | 11.2 | 2.6 | 15.4 | 2.3 | 8.2 | 4.1 | **112** | 6.7 | 1.1 | 10.2 | 0.8 | 17.8 | 0.7 |
| **113** | 11.8 | 2.7 | 10.7 | 2.1 | 9.9 | 4.6 | **113** | 10.1 | 1.5 | 6.3 | 1.1 | 18.0 | 0.9 |
| **114** | 14.7 | 2.6 | 12.6 | 1.1 | 13.2 | 2.8 | **114** | 13.1 | 1.1 | 7.0 | 0.9 | 16.3 | 0.5 |
| **115** | 18.5 | 2.7 | 12.0 | 1.1 | 15.9 | 3.8 | **115** | 16.9 | 1.0 | 5.9 | 1.0 | 16.3 | 0.6 |
| **116** | 19.0 | 3.1 | 11.9 | 0.9 | 16.9 | 3.1 | **116** | 19.3 | 1.0 | 9.8 | 1.1 | 15.7 | 0.7 |
| **117** | 22.5 | 3.0 | 10.8 | 1.3 | 17.8 | 3.5 | **117** | 19.5 | 1.4 | 11.6 | 1.1 | 16.7 | 0.7 |
| **118** | 20.6 | 3.3 | 6.9 | 1.6 | 16.0 | 3.4 | **118** | 17.4 | 0.9 | 11.9 | 1.7 | 12.2 | 0.7 |
| **119** | 24.6 | 3.2 | 6.9 | 2.6 | 17.5 | 3.8 | **119** | 17.8 | 0.9 | 14.9 | 2.2 | 13.5 | 0.7 |
| **120** | 27.9 | 3.2 | 10.6 | 2.6 | 20.8 | 4.1 | **120** | 14.0 | 1.1 | 13.4 | 2.8 | 13.9 | 0.8 |
| **121** | 28.5 | 2.9 | 9.0 | 2.6 | 18.1 | 4.4 | **121** | 12.9 | 0.8 | 15.7 | 3.7 | 8.9 | 0.8 |
| **122** | 26.9 | 2.6 | 7.8 | 2.2 | 18.3 | 4.7 | **122** | 13.8 | 1.2 | 19.4 | 3.7 | 9.5 | 0.9 |
| **123** | 27.0 | 3.6 | 6.9 | 1.8 | 17.5 | 4.7 | **123** | 12.6 | 0.7 | 18.8 | 4.4 | 6.2 | 0.7 |
| **124** | 28.4 | 2.5 | 10.1 | 1.6 | 21.5 | 4.7 | **124** | 10.8 | 0.5 | 13.7 | 4.5 | 6.4 | 0.5 |
| **125** | 32.7 | 2.4 | 15.1 | 1.7 | 23.1 | 4.9 | **125** | 7.2 | 0.6 | 12.4 | 4.3 | 10.8 | 0.5 |
| **126** | 32.0 | 2.9 | 14.0 | 1.8 | 25.3 | 5.3 | **126** | 7.7 | 0.5 | 18.5 | 4.6 | 9.8 | 0.5 |
| **127** | 36.3 | 2.9 | 17.6 | 1.9 | 25.7 | 5.4 | **127** | 12.8 | 0.6 | 20.4 | 4.9 | 4.4 | 0.4 |
| **128** | 37.5 | 3.4 | 21.1 | 1.8 | 28.1 | 4.7 | **128** | 11.6 | 1.1 | 15.7 | 5.3 | 7.8 | 0.4 |
| **129** | 35.2 | 3.9 | 22.1 | 1.5 | 31.3 | 5.3 | **129** | 15.9 | 0.8 | 16.8 | 5.3 | 7.1 | 0.6 |
| **130** | 37.6 | 3.6 | 24.3 | 1.6 | 33.5 | 4.9 | **130** | 17.1 | 1.7 | 17.0 | 6.2 | 4.2 | 0.4 |
| **131** | 39.7 | 2.8 | 28.2 | 1.5 | 37.5 | 5.0 | **131** | 17.8 | 1.1 | 20.8 | 6.3 | 10.4 | 0.6 |
| **132** | 37.9 | 3.1 | 26.8 | 1.6 | 37.0 | 5.9 | **132** | 14.6 | 0.8 | 20.3 | 5.7 | 9.1 | 0.5 |
| **133** | 35.0 | 3.4 | 24.2 | 1.7 | 34.8 | 6.6 | **133** | 12.7 | 1.0 | 21.9 | 5.6 | 8.6 | 0.5 |
| **134** | 33.6 | 3.4 | 20.9 | 1.5 | 32.8 | 5.2 | **134** | 8.7 | 0.8 | 16.7 | 5.4 | 11.6 | 0.4 |
| **135** | 35.9 | 2.8 | 23.7 | 1.6 | 37.2 | 5.1 | **135** | 8.6 | 0.6 | 21.2 | 4.9 | 14.8 | 1.0 |
| **136** | 35.2 | 3.3 | 19.9 | 1.7 | 34.1 | 4.9 | **136** | 4.3 | 0.5 | 20.6 | 5.6 | 15.6 | 0.8 |
| **137** | 40.4 | 3.5 | 26.4 | 1.7 | 39.4 | 4.8 | **137** | 8.2 | 0.7 | 23.6 | 4.8 | 19.5 | 0.6 |
| **138** | 42.9 | 3.0 | 28.3 | 1.8 | 41.7 | 4.7 | **138** | 6.6 | 1.0 | 21.6 | 4.7 | 18.1 | 1.1 |
| **139** | 44.9 | 3.4 | 31.4 | 1.7 | 44.9 | 4.7 | **139** | 10.7 | 1.3 | 23.6 | 4.2 | 22.1 | 1.2 |
| **140** | 43.8 | 3.8 | 32.6 | 1.7 | 44.1 | 4.6 | **140** | 11.7 | 1.7 | 23.0 | 3.7 | 24.4 | 0.9 |
| **Representative from cluster 3**  (distances in Å between the ligands and the C-terminal residues) | | | | | | | **Representative from cluster 4**  (distances in Å between the ligands and the C-terminal residues) | | | | | | |
| **Res. Num.** | **DCH** | | **DOP** | | **DOP-H** | | **Res.**  **Num** | **DCH** | | **DOP** | | **DOP-H** | |
|  | Av. | SD | Av. | SD | Av. | SD |  | Av. | SD | Av. | SD | Av. | SD |
| **83** | 28.2 | 3.1 | 31.7 | 3.4 | 32.9 | 3.4 | **83** | 8.2 | 0.8 | 6.0 | 1.2 | 9.2 | 0.9 |
| **110** | 14.0 | 1.7 | 20.2 | 2.7 | 13.7 | 1.6 | **110** | 30.6 | 1.0 | 30.2 | 2.0 | 26.9 | 4.0 |
| **111** | 15.1 | 1.6 | 18.3 | 2.2 | 17.7 | 1.6 | **111** | 27.8 | 1.3 | 27.0 | 1.9 | 26.0 | 2.5 |
| **112** | 16.4 | 1.4 | 17.6 | 2.1 | 18.5 | 1.7 | **112** | 28.4 | 1.1 | 25.7 | 2.1 | 24.3 | 3.1 |
| **113** | 12.8 | 1.5 | 14.6 | 1.3 | 14.0 | 1.5 | **113** | 24.2 | 1.4 | 21.1 | 2.2 | 21.0 | 2.2 |
| **114** | 17.3 | 1.2 | 16.2 | 1.2 | 18.5 | 1.6 | **114** | 20.9 | 1.1 | 21.4 | 1.3 | 24.3 | 1.1 |
| **115** | 13.5 | 1.4 | 12.7 | 1.2 | 14.6 | 1.7 | **115** | 17.6 | 0.9 | 18.2 | 1.5 | 19.9 | 1.1 |
| **116** | 17.6 | 2.0 | 16.6 | 1.6 | 14.4 | 1.5 | **116** | 16.0 | 0.8 | 15.0 | 1.4 | 17.5 | 1.9 |
| **117** | 14.8 | 2.7 | 13.2 | 1.3 | 10.3 | 1.6 | **117** | 11.5 | 0.8 | 11.4 | 1.4 | 14.4 | 0.9 |
| **118** | 18.4 | 2.1 | 16.8 | 1.8 | 14.4 | 1.5 | **118** | 11.1 | 1.0 | 11.1 | 0.8 | 12.7 | 0.6 |
| **119** | 16.8 | 2.0 | 15.3 | 1.5 | 13.7 | 1.3 | **119** | 6.9 | 1.0 | 7.1 | 0.6 | 7.7 | 0.6 |
| **120** | 16.7 | 2.5 | 15.4 | 2.0 | 12.4 | 1.6 | **120** | 8.9 | 1.1 | 9.5 | 0.6 | 10.5 | 0.6 |
| **121** | 14.2 | 1.7 | 12.7 | 1.3 | 14.3 | 1.5 | **121** | 6.3 | 0.8 | 7.0 | 1.5 | 6.6 | 0.9 |
| **122** | 10.7 | 1.3 | 11.6 | 1.3 | 12.0 | 1.5 | **122** | 9.5 | 0.5 | 9.3 | 0.5 | 9.4 | 1.0 |
| **123** | 11.8 | 1.1 | 11.5 | 1.6 | 8.9 | 1.1 | **123** | 4.3 | 0.4 | 4.6 | 0.6 | 6.2 | 1.4 |
| **124** | 7.1 | 1.2 | 7.2 | 1.6 | 8.9 | 2.0 | **124** | 5.6 | 0.7 | 5.4 | 1.2 | 4.6 | 0.6 |
| **125** | 9.3 | 0.9 | 9.8 | 1.6 | 8.6 | 1.6 | **125** | 9.6 | 0.7 | 9.1 | 0.9 | 9.7 | 1.6 |
| **126** | 5.9 | 1.5 | 10.2 | 1.5 | 5.4 | 1.7 | **126** | 8..6 | 0.8 | 10.6 | 1.0 | 10.0 | 0.9 |
| **127** | 6.8 | 0.9 | 8.7 | 1.2 | 6.6 | 0.6 | **127** | 6.2 | 0.7 | 9.3 | 1.7 | 4.2 | 1.6 |
| **128** | 6.4 | 1.0 | 6.4 | 1.3 | 9.2 | 1.4 | **128** | 4.4 | 0.6 | 10.2 | 1.7 | 2.9 | 0.7 |
| **129** | 5.8 | 0.9 | 4.9 | 0.8 | 9.7 | 1.6 | **129** | 7.8 | 0.6 | 8.7 | 2.0 | 4.9 | 0.9 |
| **130** | 8.4 | 1.9 | 5.9 | 0.7 | 9.3 | 1.8 | **130** | 10.4 | 0.7 | 12.4 | 1.1 | 9.2 | 0.6 |
| **131** | 9.7 | 2.2 | 8.2 | 0.8 | 9.7 | 1.8 | **131** | 14.9 | 0.7 | 13.2 | 1.3 | 13.8 | 0.7 |
| **132** | 9.0 | 1.1 | 9.1 | 0.8 | 9.1 | 1.2 | **132** | 11.3 | 0.8 | 9.1 | 1.5 | 9.6 | 0.8 |
| **133** | 6.3 | 1.4 | 7.1 | 0.8 | 6.3 | 1.2 | **133** | 7.6 | 0.6 | 5.8 | 0.9 | 5.9 | 0.6 |
| **134** | 6.6 | 1.3 | 4.3 | 0.4 | 5.4 | 1.1 | **134** | 6.9 | 0.6 | 4.7 | 0.7 | 6.1 | 0.6 |
| **135** | 7.0 | 1.8 | 5.5 | 1.0 | 6.9 | 1.3 | **135** | 12.5 | 0.7 | 10.2 | 0.9 | 11.0 | 0.7 |
| **136** | 10.4 | 1.3 | 7.8 | 1.5 | 9.2 | 1.5 | **136** | 10.7 | 0.7 | 9.7 | 0.9 | 10.9 | 0.8 |
| **137** | 10.8 | 2.8 | 9.9 | 1.1 | 11.0 | 1.6 | **137** | 16.3 | 0.9 | 14.3 | 1.4 | 15.8 | 0.6 |
| **138** | 12.4 | 4.2 | 9.4 | 2.8 | 12.0 | 2.0 | **138** | 19.1 | 0.8 | 17.2 | 0.9 | 18.8 | 0.8 |
| **139** | 11.7 | 5.6 | 12.5 | 3.3 | 9.9 | 3.1 | **139** | 21.4 | 0.8 | 19.9 | 1.4 | 19.5 | 1.3 |
| **140** | 12.3 | 4.3 | 12.4 | 2.6 | 14.0 | 2.7 | **140** | 25.4 | 0.8 | 18.9 | 2.0 | 17.0 | 1.9 |
| **Representative from cluster 5**  (distances in Å between the ligands and the C-terminal residues) | | | | | | | **Representative from cluster 6**  (distances in Å between the ligands and the C-terminal residues) | | | | | | |
| **Res. Num.** | **DCH** | | **DOP** | | **DOP-H** | | **Res.**  **Num** | **DCH** | | **DOP** | | **DOP-H** | |
|  | Av. | SD | Av. | SD | Av. | SD |  | Av. | SD | Av. | SD | Av. | SD |
| **83** | 8.3 | 0.9 | 10.1 | 1.9 | 21.6 | 2.7 | **83** | 23.5 | 0.8 | 16.8 | 2.9 | 19.5 | 1.9 |
| **110** | 20.8 | 2.9 | 23.3 | 3.9 | 10.0 | 2.8 | **110** | 34.8 | 0.7 | 19.8 | 5.4 | 32.8 | 1.2 |
| **111** | 22.1 | 2.4 | 21.3 | 3.1 | 9.4 | 3.9 | **111** | 29.7 | 0.6 | 15.8 | 4.8 | 28.0 | 1.3 |
| **112** | 19.7 | 2.0 | 24.7 | 2.7 | 13.1 | 3.3 | **112** | 28.3 | 0.9 | 15.2 | 4.4 | 26.8 | 1.1 |
| **113** | 24.0 | 2.5 | 24.0 | 3.0 | 11.2 | 2.4 | **113** | 24.2 | 0.6 | 11.4 | 4.6 | 24.0 | 2.4 |
| **114** | 23.3 | 2.5 | 21.7 | 3.5 | 9.7 | 2.4 | **114** | 26.9 | 0.8 | 12.8 | 4.8 | 25.3 | 1.8 |
| **115** | 19.6 | 2.1 | 17.6 | 3.3 | 8.2 | 1.7 | **115** | 27.8 | 0.6 | 15.9 | 3.9 | 25.4 | 1.2 |
| **116** | 14.9 | 2.2 | 18.2 | 3.0 | 6.0 | 0.9 | **116** | 26.5 | 0.7 | 16.7 | 3.4 | 24.4 | 0.7 |
| **117** | 15.5 | 1.6 | 14.3 | 2.9 | 5.9 | 0.9 | **117** | 24.5 | 0.6 | 18.4 | 2.7 | 23.1 | 0.8 |
| **118** | 13.1 | 2.0 | 16.1 | 2.7 | 9.5 | 1.7 | **118** | 22.7 | 0.7 | 16.8 | 3.2 | 21.1 | 0.9 |
| **119** | 12.8 | 2.2 | 13.6 | 2.6 | 12.9 | 1.3 | **119** | 18.7 | 0.5 | 13.0 | 3.0 | 17.8 | 0.6 |
| **120** | 13.4 | 1.8 | 14.2 | 2.4 | 15.2 | 1.7 | **120** | 19.3 | 0.7 | 12.0 | 4.2 | 17.6 | 0.8 |
| **121** | 11.5 | 2.3 | 10.7 | 2.3 | 18.2 | 1.8 | **121** | 15.3 | 0.5 | 12.4 | 4.7 | 13.1 | 0.7 |
| **122** | 15.0 | 2.6 | 12.8 | 2.4 | 22.9 | 1.9 | **122** | 14.6 | 0.4 | 15.1 | 3.8 | 12.5 | 1.3 |
| **123** | 12.6 | 2.1 | 10.0 | 1.5 | 23.9 | 1.8 | **123** | 10.5 | 0.4 | 15.7 | 4.1 | 8.8 | 2.0 |
| **124** | 11.6 | 2.0 | 9.2 | 1.5 | 22.0 | 2.1 | **124** | 11.3 | 0.5 | 12.0 | 4.0 | 9.3 | 0.4 |
| **125** | 6.3 | 1.5 | 7.3 | 1.8 | 23.1 | 2.7 | **125** | 10.8 | 0.6 | 13.0 | 2.5 | 9.8 | 0.4 |
| **126** | 10.3 | 1.5 | 11.7 | 1.6 | 24.1 | 3.0 | **126** | 4.0 | 0.5 | 16.5 | 3.4 | 3.9 | 0.2 |
| **127** | 10.1 | 0.7 | 12.4 | 1.7 | 27.6 | 3.3 | **127** | 6.5 | 0.5 | 16.5 | 4.6 | 4.9 | 0.3 |
| **128** | 4.4 | 0.6 | 11.4 | 1.8 | 28.3 | 4.1 | **128** | 7.9 | 0.4 | 15.4 | 3.9 | 6.8 | 0.2 |
| **129** | 8.8 | 1.3 | 9.6 | 1.1 | 29.4 | 3.0 | **129** | 7.1 | 0.3 | 19.8 | 4.1 | 7.1 | 0.3 |
| **130** | 9.9 | 1.8 | 7.3 | 0.9 | 32.2 | 2.7 | **130** | 11.3 | 0.4 | 19.1 | 4.2 | 11.2 | 0.5 |
| **131** | 13.0 | 1.6 | 5.0 | 0.4 | 31.3 | 2.2 | **131** | 11.7 | 0.5 | 17.2 | 4.0 | 11.7 | 0.4 |
| **132** | 11.6 | 1.3 | 8.0 | 0.8 | 28.2 | 2.2 | **132** | 11.3 | 0.4 | 21.6 | 4.1 | 11.3 | 0.6 |
| **133** | 8.9 | 1.2 | 5.6 | 1.0 | 25.8 | 2.2 | **133** | 11.4 | 0.5 | 24.5 | 4.3 | 11.5 | 1.0 |
| **134** | 13.4 | 1.6 | 6.4 | 1.3 | 24.5 | 1.6 | **134** | 16.2 | 0.5 | 22.3 | 3.2 | 15.7 | 1.0 |
| **135** | 11.8 | 1.4 | 9.2 | 2.4 | 20.2 | 1.4 | **135** | 17.0 | 0.4 | 23.8 | 4.1 | 16.2 | 0.8 |
| **136** | 17.4 | 1.5 | 12.7 | 1.7 | 24.3 | 2.2 | **136** | 14.1 | 0.4 | 24.2 | 4.6 | 14.0 | 0.8 |
| **137** | 17.7 | 1.8 | 13.9 | 2.2 | 20.7 | 1.8 | **137** | 11.6 | 0.6 | 26.0 | 4.1 | 11.1 | 1.0 |
| **138** | 21.3 | 1.7 | 17.0 | 1.9 | 22.4 | 3.4 | **138** | 16.0 | 0.6 | 27.6 | 4.0 | 15.5 | 1.1 |
| **139** | 23.1 | 2.2 | 16.5 | 2.7 | 22.8 | 2.0 | **139** | 17.2 | 0.5 | 31.5 | 4.0 | 17.1 | 1.0 |
| **140** | 26.6 | 2.0 | 19.7 | 2.3 | 25.1 | 1.8 | **140** | 18.9 | 0.8 | 32.4 | 4.1 | 17.9 | 1.1 |

**Table S8**. **MD simulations of the dopamine forms/AS adducts.** The average values (Av.) with their standard deviations (SD) of the distance between the center of mass of E83 along with the C-Terminal residues (from 110 to 140) and dopamine, along the trajectory, are reported here.
