# Supplementary material for: Inhibition of α-Synuclein Fibrillization by Dopamine Is Mediated by Interactions with Five C-Terminal Residues and with E83 in the NAC Region
Source: PLoS One. 2008 Oct 14;3(10):e3394. doi: 10.1371/journal.pone.0003394 (PMC2566601; doi:10.1371/journal.pone.0003394)
Supplement: Cluster Analysis S1 — Cluster Analysis of Micheletti et al. (0.04 MB DOC) [file pone.0003394.s020.doc]

**Cluster analysis of Michletti et al [1]**

Number of conformations 3062 (140 residues) - Cutoff 19.000000

Representative conf: 1552 with score 1202 (removed 0)

Representative conf: 1958 with score 473 (removed 1202)

Representative conf: 1445 with score 207 (removed 1675)

Representative conf: 746 with score 178 (removed 1882)

Representative conf: 1258 with score 129 (removed 2060)

Representative conf: 1484 with score 102 (removed 2189)

Representative conf: 2793 with score 94 (removed 2291)

Representative conf: 2515 with score 93 (removed 2385)

Representative conf: 2828 with score 54 (removed 2478)

Representative conf: 197 with score 45 (removed 2532)

Representative conf: 358 with score 39 (removed 2577)

Representative conf: 67 with score 30 (removed 2616)

Representative conf: 2574 with score 30 (removed 2646)

Representative conf: 310 with score 28 (removed 2676)

Representative conf: 1621 with score 27 (removed 2704)

Representative conf: 513 with score 23 (removed 2731)

Representative conf: 2609 with score 21 (removed 2754)

Representative conf: 941 with score 18 (removed 2775)

Representative conf: 263 with score 17 (removed 2793)

Representative conf: 2643 with score 16 (removed 2810)

Representative conf: 2635 with score 14 (removed 2826)

Representative conf: 1324 with score 13 (removed 2840)

Representative conf: 715 with score 12 (removed 2853)

Representative conf: 2486 with score 12 (removed 2865)

Representative conf: 319 with score 10 (removed 2877)

Representative conf: 1789 with score 10 (removed 2887)

Representative conf: 53 with score 9 (removed 2897)

Representative conf: 2859 with score 9 (removed 2906)

Representative conf: 191 with score 8 (removed 2915)

Representative conf: 3 with score 7 (removed 2923)

Representative conf: 509 with score 7 (removed 2930)

Representative conf: 629 with score 7 (removed 2937)

Representative conf: 226 with score 6 (removed 2944)

Representative conf: 822 with score 6 (removed 2950)

Representative conf: 1541 with score 6 (removed 2956)

Representative conf: 3047 with score 6 (removed 2962)

Representative conf: 526 with score 5 (removed 2968)

Representative conf: 1231 with score 5 (removed 2973)

Representative conf: 1837 with score 5 (removed 2978)

Representative conf: 643 with score 4 (removed 2983)

Representative conf: 727 with score 4 (removed 2987)

Representative conf: 1222 with score 4 (removed 2991)

Representative conf: 329 with score 3 (removed 2995)

Representative conf: 552 with score 3 (removed 2998)

Representative conf: 813 with score 3 (removed 3001)

Representative conf: 1255 with score 3 (removed 3004)

Representative conf: 1259 with score 3 (removed 3007)

Representative conf: 1923 with score 3 (removed 3010)

Representative conf: 2020 with score 3 (removed 3013)

Representative conf: 2281 with score 3 (removed 3016)

Representative conf: 527 with score 2 (removed 3019)

Representative conf: 591 with score 2 (removed 3021)

Representative conf: 781 with score 2 (removed 3023)

Representative conf: 1460 with score 2 (removed 3025)

Representative conf: 1635 with score 2 (removed 3027)

Representative conf: 1643 with score 2 (removed 3029)

Representative conf: 245 with score 1 (removed 3031)

Representative conf: 317 with score 1 (removed 3032)

Representative conf: 365 with score 1 (removed 3033)

Representative conf: 381 with score 1 (removed 3034)

Representative conf: 568 with score 1 (removed 3035)

Representative conf: 588 with score 1 (removed 3036)

Representative conf: 601 with score 1 (removed 3037)

Representative conf: 803 with score 1 (removed 3038)

Representative conf: 807 with score 1 (removed 3039)

Representative conf: 842 with score 1 (removed 3040)

Representative conf: 999 with score 1 (removed 3041)

Representative conf: 1184 with score 1 (removed 3042)

Representative conf: 1232 with score 1 (removed 3043)

Representative conf: 1333 with score 1 (removed 3044)

Representative conf: 1392 with score 1 (removed 3045)

Representative conf: 1538 with score 1 (removed 3046)

Representative conf: 1609 with score 1 (removed 3047)

Representative conf: 1742 with score 1 (removed 3048)

Representative conf: 1797 with score 1 (removed 3049)

Representative conf: 1897 with score 1 (removed 3050)

Representative conf: 2018 with score 1 (removed 3051)

Representative conf: 2076 with score 1 (removed 3052)

Representative conf: 2078 with score 1 (removed 3053)

Representative conf: 2154 with score 1 (removed 3054)

Representative conf: 2263 with score 1 (removed 3055)

Representative conf: 2268 with score 1 (removed 3056)

Representative conf: 2451 with score 1 (removed 3057)

Representative conf: 2452 with score 1 (removed 3058)

Representative conf: 2466 with score 1 (removed 3059)

Representative conf: 2638 with score 1 (removed 3060)

Representative conf: 2717 with score 1 (removed 3061

Total representatives: 87 Total number of conf. 3062

References

1. Micheletti C., Seno F., Maritan A. (2000) Recurrent oligomers in proteins - an optimal scheme reconciling accurate and concise backbone representations in automated folding and design studies. Proteins: Str Func and Genet 40: 662-674.
