# Supplementary material for: Inhibition of α-Synuclein Fibrillization by Dopamine Is Mediated by Interactions with Five C-Terminal Residues and with E83 in the NAC Region
Source: PLoS One. 2008 Oct 14;3(10):e3394. doi: 10.1371/journal.pone.0003394 (PMC2566601; doi:10.1371/journal.pone.0003394)
Supplement: Cluster Analysis S2 — Cluster analysis of Kelley et al. (0.04 MB DOC) [file pone.0003394.s021.doc]

**Cluster analysis of Kelley et al [1]**

**Cluster**

Total number of conformations: 3062

Number of conformations taken randomly for the cluster procedure: 1000

Total of outliers[[1]](#footnote-2) after the cluster procedure: 160

Cluster 1

• Representative structure: 3552.pdb (688)

• Spread: 18.119

• Members:

11 16 60 159 198 230 254 264 287 296 317 327 388 427 461 485 489 504 554 598 663 668 671 672 688 689 707 734 735 742 744 763 764 791 931 958 964 973 991 996

• Number of Members: 40

Cluster2

• Representative structure: 3212.pdb (542)

• Spread: 18.645

• Members:

2 9 21 117 137 171 274 291 295 314 380 385 404 429 436 439 464 473 500 538 542 563 584 585 636 650 669 687 719 731 749 766 779 801 813 822 868 895 966 976

• Number of Members: 40

Cluster3

• Representative structure: 0934.pdb (437)

• Spread: 18.298

• Members:

35 99 138 146 148 154 202 207 233 239 278 279 283 321 329 357 367 374 398 437 516 518 524 534 536 548 622 714 785 821 861 875 916

• Number of Members: 33

Cluster4

• Representative structure: 1058.pdb (93)

• Spread: 18.695

• Members:

5 62 72 77 93 108 134 135 143 167 196 209 271 304 336 369 448 506 545 567 583 621 639 703 780 808 839 880 882 890 925 953

• Number of Members: 32

Cluster5

• Representative structure: 2627.pdb (702)

• Spread: 17.974

• Members:

1 17 48 182 229 236 255 269 270 286 308 341 402 415 416 444 507 547 558 571 578 596 702 721 748 826 913 955 994

• Number of Members: 29

Cluster6

• Representative structure: 1307.pdb (477)

• Spread: 18.420

• Members:

50 98 122 151 152 224 240 277 300 324 339 373 377 403 477 481 552 600 608 647 699 705 728 751 836 892 981 998

• Number of Members: 28

Cluster 7

• Representative structure: 3294.pdb (977)

• Spread: 18.416

• Members:

7 105 157 168 215 227 293 297 319 335 358 365 433 442 455 510 513 515 555 645 690 896 914 915 977

• Number of Members: 25

Cluster 8

• Representative structure: 0715.pdb (96)

• Spread: 18.302

• Members:

22 82 92 94 96 133 211 222 247 259 280 332 413 422 459 468 527 613 617 712 715 782 831 898

• Number of Members: 24

Cluster 9

• Representative structure: 0386.pdb (311)

• Spread: 18.079

• Members:

31 66 115 181 199 238 242 246 257 311 337 351 352 409 486 537 682 684 778 920

• Number of Members: 20

Cluster 10

• Representative structure: 3248.pdb (188)

• Spread: 18.779

• Members:

144 179 188 190 232 261 383 390 469 470 638 642 653 657 706 758 775 840 848 873

• Number of Members: 20

Cluster 11

• Representative structure: 8000.pdb (1001)

• Spread: 18.520

• Members:

38 64 85 111 129 139 176 187 318 350 451 491 517 523 828 838 919 933 1001

• Number of members: 19

Cluster12

• Representative structure: 2778.pdb (303)

• Spread: 18.622

• Members:

18 40 71 120 210 231 243 303 342 379 419 503 562 564 635 757 781 866 891

• Number of members: 19

Cluster13

• Representative structure: 2041.pdb (76)

• Spread: 18.472

• Members:

55 76 131 156 251 349 393 406 505 522 539 592 616 695 717 800 853 936

• Number of members: 18

Cluster14

• Representative structure: 3143.pdb (83)

• Spread: 17.817

• Members:

24 37 44 83 112 125 161 166 180 372 535 604 628 691 790 907 978

• Number of members: 18

Cluster15

• Representative structure: 0848.pdb (356)

• Spread: 18.029

• Members:

59 70 170 356 362 368 450 480 530 566 586 667 730 737 874 912 999

• Number of members: 17

Cluster16

• Representative structure: 1264.pdb (560)

• Spread: 17.616

• Members:

34 36 43 78 228 263 299 412 508 560 643 649 652 865 889 900

• Number of members: 16

Cluster17

• Representative structure: 0858.pdb (532)

• Spread: 18.042

• Members:

25 218 268 294 334 452 532 573 606 685 761 792 803 818 835 952

• Number of members: 16

Cluster18

• Representative structure: 1362.pdb (961)

• Spread: 18.242

• Members:

88 220 253 281 330 353 389 400 411 430 646 793 816 841 897 961

• Number of members: 16

References

1. Kelley L.A., Gardner S.P., Sutcliffe M.J. (1996) An automated approach for clustering an ensemble of NMR-derived protein structures into conformationally related subfamilies. Protein Eng 9: 1063-1065.

1. [↑](#footnote-ref-2)
